# Alzheimer's disease: a comprehensive review of epidemiology, risk factors, symptoms diagnosis, management, caregiving, advanced treatments and associated challenges

**DOI:** 10.3389/fmed.2024.1474043

**Published:** 2024-12-16

**Authors:** Saeid Safiri, Amir Ghaffari Jolfayi, Asra Fazlollahi, Soroush Morsali, Aila Sarkesh, Amin Daei Sorkhabi, Behnam Golabi, Reza Aletaha, Kimia Motlagh Asghari, Sana Hamidi, Seyed Ehsan Mousavi, Sepehr Jamalkhani, Nahid Karamzad, Ali Shamekh, Reza Mohammadinasab, Mark J. M. Sullman, Fikrettin Şahin, Ali-Asghar Kolahi

**Affiliations:** ^1^Neurosciences Research Center, Aging Research Institute, Tabriz University of Medical Sciences, Tabriz, Iran; ^2^Social Determinants of Health Research Center, Department of Community Medicine, Faculty of Medicine, Tabriz University of Medical Sciences, Tabriz, Iran; ^3^Cardiovascular Research Center, Rajaie Cardiovascular, Medical, and Research Center, Iran University of Medical Sciences, Tehran, Iran; ^4^Student Research Committee, Tabriz University of Medical Sciences, Tabriz, Iran; ^5^Tabriz USERN Office, Universal Scientific Education and Research Network (USERN), Tabriz, Iran; ^6^Research Center for Integrative Medicine in Aging, Aging Research Institute, Tabriz University of Medical Sciences, Tabriz, Iran; ^7^Department of Persian Medicine, School of Traditional, Medicine, Tabriz University of Medical Sciences, Tabriz, Iran; ^8^Nutrition Research Center, Tabriz University of Medical Sciences, Tabriz, Iran; ^9^Department of History of Medicine, School of Traditional Medicine, Tabriz University of Medical Sciences, Tabriz, Iran; ^10^Department of Life and Health Sciences, University of Nicosia, Nicosia, Cyprus; ^11^Department of Social Sciences, University of Nicosia, Nicosia, Cyprus; ^12^Department of Genetics and Bioengineering, Faculty of Engineering, Yeditepe University, Istanbul, Türkiye; ^13^Social Determinants of Health Research Center, Shahid Beheshti University of Medical Sciences, Tehran, Iran

**Keywords:** Alzheimer's disease, risk factors, diagnosis, management, caregivers

## Abstract

**Background:**

Alzheimer's disease (AD) is a chronic, progressive neurodegenerative disorder characterized by cognitive decline, memory loss, and impaired reasoning. It is the leading cause of dementia in older adults, marked by the pathological accumulation of amyloid-beta plaques and neurofibrillary tangles. These pathological changes lead to widespread neuronal damage, significantly impacting daily functioning and quality of life.

**Objective:**

This comprehensive review aims to explore various aspects of Alzheimer's disease, including its epidemiology, risk factors, clinical presentation, diagnostic advancements, management strategies, caregiving challenges, and emerging therapeutic interventions.

**Methods:**

A systematic literature review was conducted across multiple electronic databases, including PubMed, MEDLINE, Cochrane Library, and Scopus, from their inception to May 2024. The search strategy incorporated a combination of keywords and Medical Subject Headings (MeSH) terms such as “Alzheimer's disease,” “epidemiology,” “risk factors,” “symptoms,” “diagnosis,” “management,” “caregiving,” “treatment,” and “novel therapies.” Boolean operators (AND, OR) were used to refine the search, ensuring a comprehensive analysis of the existing literature on Alzheimer's disease.

**Results:**

AD is significantly influenced by genetic predispositions, such as the apolipoprotein E (APOE) ε4 allele, along with modifiable environmental factors like diet, physical activity, and cognitive engagement. Diagnostic approaches have evolved with advances in neuroimaging techniques (MRI, PET), and biomarker analysis, allowing for earlier detection and intervention. The National Institute on Aging and the Alzheimer's Association have updated diagnostic criteria to include biomarker data, enhancing early diagnosis.

**Conclusion:**

The management of AD includes pharmacological treatments, such as cholinesterase inhibitors and NMDA receptor antagonists, which provide symptomatic relief but do not slow disease progression. Emerging therapies, including amyloid-beta and tau-targeting treatments, gene therapy, and immunotherapy, offer potential for disease modification. The critical role of caregivers is underscored, as they face considerable emotional, physical, and financial burdens. Support programs, communication strategies, and educational interventions are essential for improving caregiving outcomes. While significant advancements have been made in understanding and managing AD, ongoing research is necessary to identify new therapeutic targets and enhance diagnostic and treatment strategies. A holistic approach, integrating clinical, genetic, and environmental factors, is essential for addressing the multifaceted challenges of Alzheimer's disease and improving outcomes for both patients and caregivers.

## 1 Introduction

Alzheimer's disease (AD) is a progressive neurodegenerative disorder that causes memory loss and other cognitive impairments, such as deterioration in language, learning, memory, visual-spatial abilities, reasoning, and behavior (1). The decline in cognitive abilities can become severe enough to interfere with daily activities. AD is the most prevalent form of dementia, contributing to at least two-thirds of dementia cases among individuals aged 65 and older (2, 3). The pathological hallmarks of AD are neurofibrillary tangles (NFTs), which are formed by hyperphosphorylated tau protein within neurons, and extracellular plaques composed of accumulated β-amyloid (Aβ) peptide (3, 4).

In 1906, Alois Alzheimer, a German doctor, published his now-famous case study. He carefully detailed the symptoms of a 51-year-old woman named Auguste Deter, who was in his care at the state asylum in Frankfurt, Germany (5). The neuropathologic analysis of Alzheimer's patients revealed widespread brain degeneration and specific changes in cortical cellular bundles. He presented his research titled “On the peculiar disease process of the cerebral cortex” (6). Since then, progress has been made in our understanding of the disease that bears his name, along with its neuropsychological effects (7). In 1984, Dr. George Glenner and Dr. Cain Wong identified amyloid protein as the primary constituent of extracellular plaques (8). In 1986, researchers discovered that the abnormal hyperphosphorylation of tau protein results in the neurofibrillary tangles characteristic of Alzheimer's. Tau protein is a type of protein that maintains microtubules and is released during neurodegeneration (9, 10). In 1993, Tacrine (Cognex) became the first drug authorized by the FDA to address the cognitive symptoms of Alzheimer's, such as thinking and memory (11). It is essential to note that the clinical diagnostic criteria for AD were updated in 1984, 2011, 2018, and 2024 to reflect the growing availability of biomarkers and improved ability to identify preclinical disease episodes (12–15). In the most recent update in 2024, AD as described as beginning as an asymptomatic biological process with AD neuropathologic changes (ADNPC), progressing to clinical symptoms as the neuropathologic burden increases. Early-changing Core 1 biomarkers, such as amyloid PET, cerebrospinal fluid, and plasma biomarkers, reflect ADNPC and are sufficient for diagnosis and clinical decision-making. Later-changing Core 2 biomarkers provide prognostic insights and increase confidence that AD is contributing to symptoms, with an integrated staging scheme accounting for factors like copathologies and cognitive reserve (16).

In the coming decades, Alzheimer's care will likely remain a significant public health concern (17). Due to this ongoing and future concern, increasing knowledge and research about AD could be effective through various approaches, such as identifying and managing risk factors, and updating methods for early diagnosis and appropriate treatment. Gaining further insight into the aging process and alterations in brain function, along with evaluating strategies to halt disease progression, could lead to improved approaches to this challenging disease. In this study, we aim to present a comprehensive review of AD, examining its epidemiology, genetics, underlying environmental factors, symptoms, various diagnostic techniques, treatment, challenges, and concerns.

## 2 Methods

### 2.1 Study design

This comprehensive review synthesizes existing literature on Alzheimer's disease (AD), covering its epidemiology, risk factors, symptoms, diagnosis, management, caregiving, and treatments.

### 2.2 Literature search strategy

A systematic search was performed across multiple electronic databases, including PubMed, MEDLINE, Cochrane Library, and Scopus, from their inception to May 2024. The search strategy combined keywords and Medical Subject Headings (MeSH) terms related to Alzheimer's disease, such as “Alzheimer's disease,” “epidemiology,” “risk factors,” ‘symptoms,” “diagnosis,” “management,” “caregiving,” “treatment,” and “novel therapies.” Boolean operators (AND, OR) were used to refine the search and ensure comprehensive coverage of relevant articles.

### 2.3 Inclusion and exclusion criteria

Studies were included if they were peer-reviewed articles, reviews, or meta-analyses focusing on Alzheimer's disease, published in English, and addressed any of the following aspects of AD: epidemiology, risk factors, symptoms, diagnosis, management, caregiving, current treatments, and novel treatments. Exclusion criteria were non-peer-reviewed articles, editorials, commentaries, and conference abstracts, studies not related to Alzheimer's disease, and articles not available in English.

### 2.4 Data extraction and synthesis

Full texts of potentially relevant studies were obtained and assessed for eligibility based on the inclusion and exclusion criteria. Data extracted from each study included epidemiological data (prevalence and incidence rates, demographic factors), risk factors (genetic, environmental, and lifestyle factors), symptoms and diagnosis (clinical features, diagnostic criteria, biomarkers, imaging techniques), management and caregiving (treatment approaches, caregiving challenges) and treatment data (efficacy and safety of current treatments, novel therapeutic approaches, clinical trial outcomes). A narrative synthesis was conducted to summarize findings across studies.

### 2.5 Ethical considerations

As this study involves a review of existing literature, ethical approval was not required. However, ethical standards were maintained by ensuring proper citation and acknowledgment of all sources.

## 3 Global epidemiology and trends of Alzheimer's disease and other dementias

**Figure 1:**
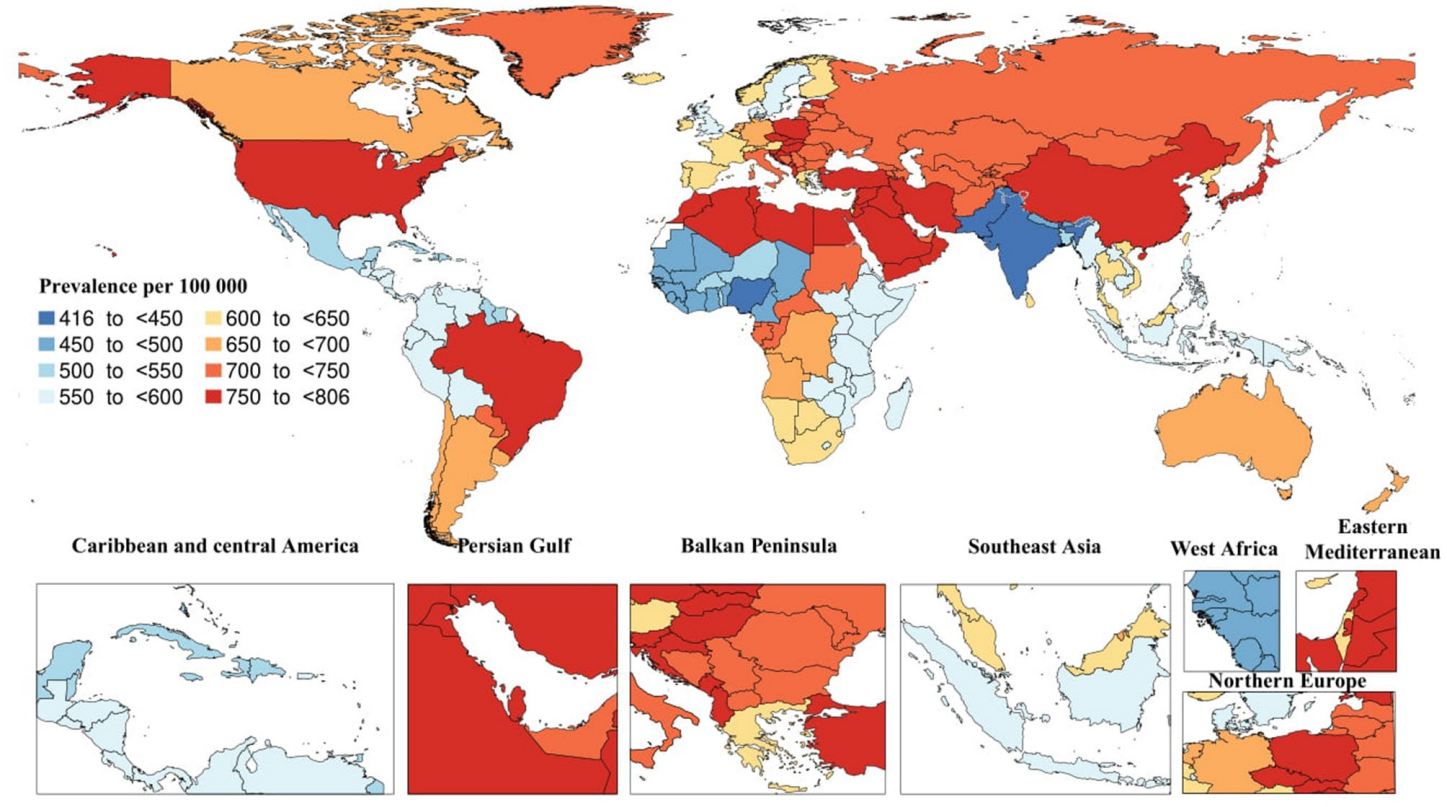
Age-standardized prevalence of Alzheimer's disease per 100,000 population in 2019 by country. (Generated from data available from http://ghdx.healthdata.org/gbd-results-tool).

In 2019, there was and estimated total of 51.6 million [95% uncertainty interval (UI): 44.3–59] prevalent cases of AD and other dementias. The age-standardized prevalence rate due to AD and other dementias was estimated to be 682.5 (95% UI: 585.2–782.7) per 100,000 individuals, a 5.7% increase since 1990. In the same year, the regional age-standardized prevalence rate due to AD and other dementias ranged from 428.4 to 781.4. East Asia [781.4 (95% UI: 660–904.1)] and North Africa and Middle East [777.6 (95% UI: 660.8–896)] had the highest regional age-standardized prevalence rates, while South Asia [428.4 (95% UI: 365–494)], Western Sub-Saharan Africa [469.8 (95% UI: 402.1–539.4)] had the lowest rates. Moreover, East Asia [28.3% (95% UI: 25–31.4)] and High-income Asia Pacific [19% (95% UI: 16.9–21.5)] had the most significant increases in regional age-standardized prevalence rate since 1990, while the only region with a decrease was Western Sub-Saharan Africa [−2.4% (95% UI: −3.8 to −1.1)]. In 2019, the highest national age-standardized prevalence rates were seen in Turkey [805.1 (95% UI: 686.6–926.2)] and Bahrain [801.1 (95% UI: 684.2–925.8)], while the lowest rates were found in India [416.4 (95% UI: 354.9–479.8)] and Nigeria [441.4 (95% UI: 379.6–503.6)]. Furthermore, China [29.2% (95% UI: 26–32.5)] and Japan [22.3% (95% UI: 20–24.9)] had the largest increases in national age-standardized prevalence rates since 1990, while Spain [−13% (95% UI: −18.2 to −7.2)] and Luxembourg [−10.7% (95% UI: −16.1 to −5.7)] had the largest decreases (Figure 1).

**Figure 2:**
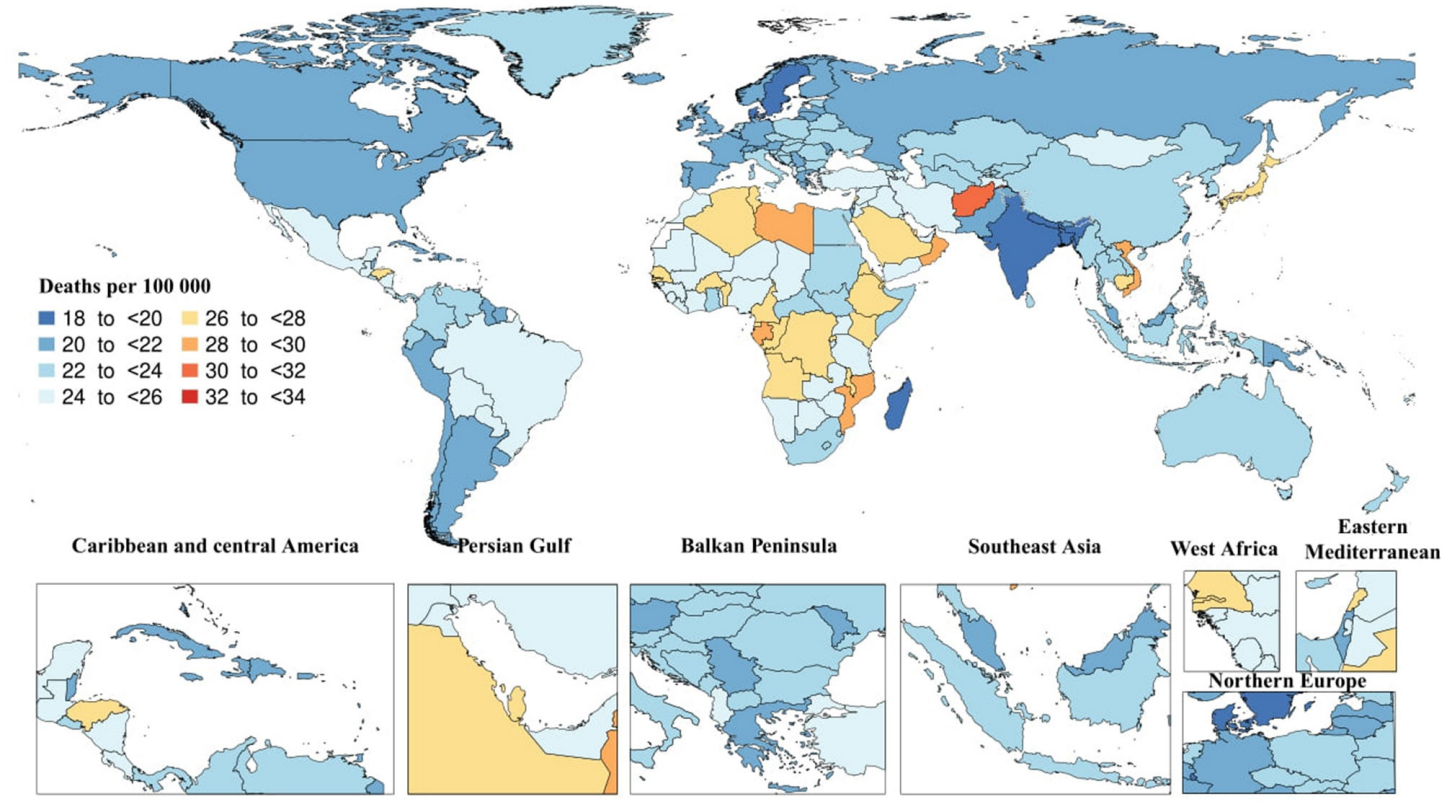
Age-standardized death rate of Alzheimer's disease per 100,000 population in 2019 by country. (Generated from data available from http://ghdx.healthdata.org/gbd-results-tool).

In 2019, there were 1.6 million (95% UI: 0.4–4.2) death cases due to AD and other dementias. The age-standardized death rate was 22.9 (95% UI: 5.8–59.2), which did not substantially change since 1990. In the same year, the regional age-standardized death rate due to AD and other dementias varied from 19.2 to 27. High-income Asia Pacific [27 (95% UI: 7.5–65.4)] and Central Sub-Saharan Africa [26.6 (95% UI: 6.7–71.4)] had the highest regional age-standardized death rates, while South Asia [19.2 (95% UI: 4.6–52.1)] and Caribbean [20.8 (95% UI: 5.3–51.9)] had the lowest rates. Moreover, High-income Asia Pacific [23.5% (95% UI: 11.9–38.6)] and Eastern Sub-Saharan Africa [14.4% (95% UI: 4.5–27.9)] had the highest increases in regional age-standardized death rates since 1990, while there were no decreases in age-standardized death rates. In 2019, the highest national age-standardized death rates were seen in Kiribati [33.3 (95% UI: 8–90.7)] and Afghanistan [30.8 (95% UI: 7.5–82.3)], while the lowest rates were found in Bangladesh [18.2 (95% UI: 4.5–49.3)] and India [19.1 (95% UI: 52–4.6)]. Furthermore, Eritrea [52.6% (95% UI: 11.2–120.9)] and Equatorial Guinea [34.2% (95% UI: 1.6–84.7)] had the highest increases in national age-standardized death rates since 1990, while Germany [−18.4% (95% UI: −27.8 to −9.1)] and Guam [−16.8% (95% UI: −28.8 to −1.3)] had the largest decreases (Figure 2)

**Figure 3:**
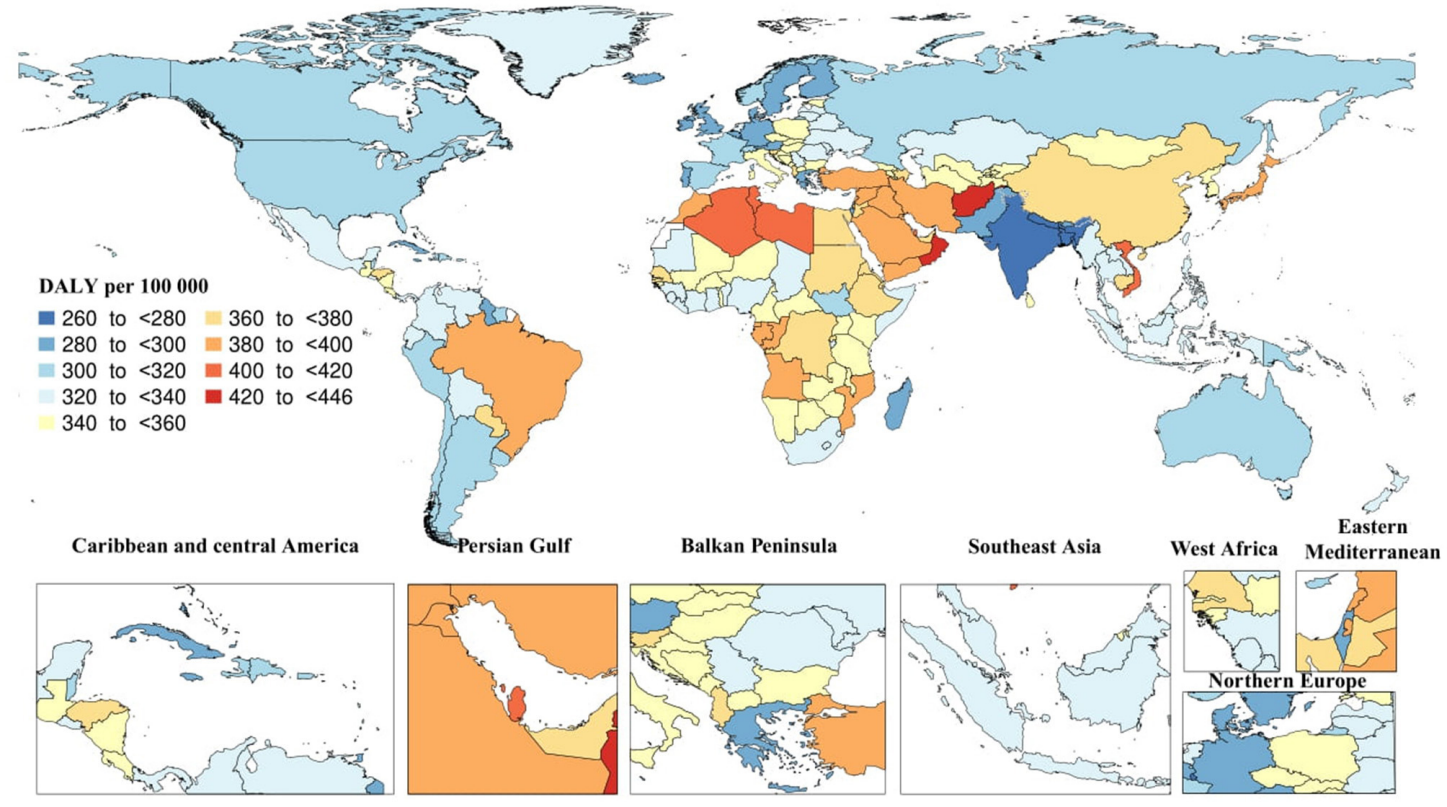
Age-standardized disability-adjusted life years (DALY) rate of Alzheimer's disease per 100,000 population in 2019 by country. (Generated from data available from: http://ghdx.healthdata.org/gbd-results-tool).

In 2019, there were 25.3 million (95% UI: 11.2–54.6) DALYs due to AD and other dementias. The global age-standardized DALY rate was 338.6 (95% UI: 151–731.3), which did not substantially change since 1990. Tropical Latin America [390.9 (95% UI: 172–856)] and North Africa and Middle East [387 (95% UI: 172–848.5)] had the highest regional age-standardized DALY rates, while South Asia [262.1 (95% UI: 105.6–617.4)] and Caribbean [299.5 (95% UI: 126.9–661.7)] had the lowest rates. Moreover, High-income Asia Pacific [19.5% (95% UI: 10.5–26.1)] and Eastern Sub-Saharan Africa [10.1% (95% UI: 2.4–18.6)] had the largest increases in regional age-standardized DALY rates since 1990, while there were no decreases in age-standardized DALY rates. In 2019, the highest national age-standardized DALY rates were seen in Kiribati [445.1 (95% UI: 167.3–1,081.5)] and Afghanistan [432.7 (95% UI: 179–1,002)], while the lowest rates were found in India [260.3 (95% UI: 103.5–616.9)] and Bangladesh [263.1 (95% UI: 114.3–589.7)]. Furthermore, Eritrea [33.4% (95% UI: 5.5–75)] and Japan [23.2% (95% UI: 13.5–31.1)] had the largest increases in national age-standardized DALY rates since 1990, while Spain [−16.2% (95% UI: −20.8 to −10.5)] and Philippines [−12.6% (95% UI: −22.5 to −1.1)] had the largest decreases (Figure 3).

The global prevalent cases and DALYs of dementia increased with age, peaked in the 80–84 age group, and then decreased with aging. Similarly, global death cases of dementia peaked in the 85–89 age group and then declined with aging. The age-specific prevalence, death, and DALY rates increased with advancing age. Furthermore, no substantial differences between males and females were associated with age-specific prevalence, death, or DALY rates.

Between 1990 and 2019, no apparent linear correlation was observed between the age-standardized DALY rate of dementia and the SDI at the regional level. Also, in 2019, a non-linear relationship was observed between the age-standardized DALY rates of dementia and the SDI of various countries and territories.

It should be noted that mild cognitive impairment (MCI) caused by AD can progress to AD dementia, with 40–75% of MCI cases linked to AD, depending on the population and whether biomarkers were used alongside clinical diagnosis (18).

## 4 Genetics of Alzheimer's disease

Despite the overall sporadic nature of the disease, many studies have shown that Genetic mutations and gene variance have been linked to an increased risk of acquiring AD. Most cases of Alzheimer's do not have a single genetic cause, and this gene variance can affect the probability of developing the disease (19). Furthermore, AD is complex and influenced by many factors, including lifestyle and environmental variables (20). Aging is the most significant biological risk factor for acquiring AD later in life. Accordingly, up to 82% of individuals are diagnosed above the age of 65 known as late-onset Alzheimer's disease (LOAD) (21). A genetic etiology of up to 100% is found in 10% of AD patients who are diagnosed before reaching the age of 65, known as early-onset Alzheimer's disease (EOAD) (21, 22). Between 35 and 60% of EOAD individuals have first-degree relatives who have dementia (23, 24). Significant mutations that lead to EOAD are genes coding the amyloid precursor protein (APP) on chromosome 21q, presenilin 1 on 14q, and presenilin 2 on 1q (25).

### 4.1 β-amyloid precursor protein

β-Amyloid Precursor Protein (βAPP), a transmembrane protein, might have three different fates due to three distinct protease activities. α-secretase and β-secretase are cell surface proteases, whereas γ-secretase is an abnormal protease that cleaves membrane proteins inside their transmembrane regions. Ninety percentage of APP is cleaved primarily by the α -secretase, preventing the synthesis of the Aβ peptide. The remaining 10% of APP is cleaved by β and γ secretases to create either the harmless Aβ 40 peptide or the Aβ 42 peptide. Because the Aβ 42 peptide is more likely to aggregate than its A40 form, it is believed to be neurotoxic (26). Regarding previous statements, mutations in the gene encoding APP could cause substantial excess production of either total Aβ or increased Aβ-40/Aβ-42 ratio. This rise causes the accumulation of neurotoxic Aβ 42, which, based on the “amyloid hypothesis”, is the key and initial pathogenic event in all types of AD, monogenic or sporadic (8, 27). This hypothesis is supported by the fact that Down syndrome individuals with three copies of the APP gene (on chromosome 21) generally acquire the neuropathological abnormalities of AD by age 40 (28). Less than one percent of early-onset individuals have pathogenic mutations in APP (21, 29). In addition to harmful mutations, a protective variant called p. A673T was found in APP among Icelandic individuals. This variant is linked to a decrease in the production of the Aβ1-40 and Aβ1-42 peptides by ~40% (30).

### 4.2 The presenilin 1 and 2 genes

PSEN1 and PSEN2 are both required proteins of the γ -secretase complex's catalytic core, which initiates the cleavage of APP (29). Mutant γ -secretase significantly shifts in Aβ1- 42/Aβ1- 40 ratio; due to that, mutations in PSEN1 and PSEN2 genes are related to AD pathogenesis (31). The significant difference between PSEN1 and 2 is the age at the onset, which PSEN1 is distinguished by, on average, 8.4 and 14.2 years younger onset than APP and PSEN2 mutations (32, 33). Mutations in PSEN1 causes the majority of familial EOAD cases. About 6% of EOAD patients have PSEN1 mutations, which cause the most severe type of AD (21). In contrast, PSEN2 mutations are uncommon, accounting for <1% of EOAD patients, and they may exhibit incomplete penetrance (21). Besides the three recognized causative AD genes, other well-replicated genes, including uncommon heterozygous mutations with low penetrance, affect the development of familial AD (34).

### 4.3 Apolipoprotein E

APOE gene on chromosome 19q13, which encodes the protein that makes up numerous plasma lipoproteins, was shown to be the primary susceptible gene in individuals with familial LOAD (21, 35–37). The three frequently encountered isoforms of APOE are ε2, –ε3, and –ε4. The most frequent form is ε3/ε3, which does not affect the development of AD (38). Nearly 40% of AD patients have the APOE 4 allele, demonstrating a significant risk-increasing impact of this isoform, which is also linked to the earlier onset of the disease (38, 39). However, this association is dose-dependent, meaning that if a person has two copies of ε4, they are more prone to experience symptoms at a younger age compared to someone with only one copy (36). So individuals who are homozygote for the ε4/ε4 gene tend to develop AD 10–15 years earlier than the average population (26). The ε2 allele, which is less common, seems to exert protective and beneficial effects when inherited alongside the ε3 allele, as opposed to individuals who carry two copies of the ε3 allele (40, 41). It is intriguing to note that while there is extensive molecular evidence linking APOE protein to AD-specific pathways, associations have been found between mutations in the APOE gene and the risk of various other neuropsychiatric disorders such as multiple sclerosis (42) and Parkinson's disease (43). Additionally, the APOE gene has supposed to be linked to age-related macular degeneration (44), several cardiovascular and cerebrovascular diseases (45, 46), and even longevity (41, 47).

### 4.4 Inheritance patterns of AD

A person with autosomal dominant inheritance has a 50% probability of transferring the disease-causing mutation to each offspring (48). If a person receives two mutant copies of a gene, one from each parent, the condition is said to be autosomal recessive. This would imply that while neither parent exhibits signs of the disorder, both parents contain a copy of a defective gene (22). Due to the identification of highly penetrant dominant AD-causing mutations, it has become widely believed that all instances of EOAD are caused by dominant alleles (49). It has been found through epidemiologic data that this belief is not consistent. Only about 10% of all cases follow this pattern, and the remaining 90% stay unexplained (50). A study carried out in 2012 demonstrated the genetic aspects of EOAD and the probable mechanism of inheritance by analyzing concordance between parents and offspring. Wingo et al. concluded that EOAD is completely linked to genetic mutations, but they declined an entirely dominant inheritance. They reported that EOAD is caused by autosomal recessive in most cases, and just 10% of individuals may be attributed to dominant variants (22). Autosomal inheritance is associated with EOAD, while LOAD inherits through a complex inheritance pattern, which refers to the complex nature of AD, that has been influenced by multiple factors, including genetic, environmental factors, and lifestyle (51).

## 5 Environmental and behavioral factors

Several environmental and behavioral factors can potentially influence AD, including smoking, poor sleep quality, chronic stress, physical inactivity, excessive alcohol consumption, obesity, poor diet, and social isolation, which are detailed further below.

### 5.1 Smoking

Although smoking is primarily considered a risk factor for a variety of cardiovascular and pulmonary diseases, it is now undeniable that the negative health consequences of smoking extend beyond the aforementioned conditions to include some unexpected ones, such as neurocognitive and neurobiological conditions (52, 53). Despite the lack of research specifically investigating the negative effects of smoking on neurocognition and neurobiology, current evidence suggests that chronic smoking is associated with learning and memory deficits, global brain atrophy, and deficiencies in executive and intellectual abilities, all of which can precipitate the development of various neurodegenerative disorders, including AD (52). Accounting for 14% of AD cases worldwide, lifetime smoking is associated with earlier symptom onset and a 70% greater risk of AD development, and the risk increases significantly with higher cumulative smoking exposure (54, 55). Although these estimates are significant enough to consider smoking as an important risk factor for AD, it should be noted that there is even a possibility of an underestimation in the magnitude of the risk due to an unavoidable survival bias, which means that smokers who pass the selection process to enter the studies are probably healthier than those who are overlooked, and competing bias, which implies that the development of AD in studied smokers is precluded by smoking-related mortality (56).

Numerous potential mechanisms may contribute to smoking-related AD neuropathology, either directly or indirectly (52). To begin with, cigarette smoke contains several potentially cytotoxic and oxidizing constituents (e.g., phenolic compounds, nitrosamines, carbon monoxide, and free radicals) (57). Second, it raises the concentrations of proinflammatory cytokines in various parts of the brain, producing more free radicals (58). Third, it lowers the concentrations of glutathione and other antioxidants in the brain due to the increased demand to scavenge the amplified concentrations of free radicals (59). All these mechanisms, along with a few others that play minor roles, subject the brain to high levels of oxidative stress, which increases Aβ production and tau phosphorylation, potentially initiating the AD pathological process (60, 61).

### 5.2 Poor sleep quality

Clinical studies suggest a bidirectional link between sleep and circadian sleep-wake rhythm disruptions and the developing of AD (62, 63). Poor sleep quality can exacerbate cognitive impairment in AD patients, and the risk of sleep disturbances rises dramatically as the disease progresses (64).

There is growing evidence linking sleep disturbances to the pathogenesis of AD (65). Several studies have demonstrated that sleep disturbances can increase Aβ levels in animal disease models and humans (66). A cross-sectional study indicated sleeping for 6 h or less was associated with more Aβ accumulation and decreased cognitive performance, particularly in memory (67).

Although sleep disturbances in AD are assumed to stem from pathological processes arising from Aβ accumulation, there may also be a bidirectional link between sleep-wake disruption and tau pathology (68). Lucey et al. found that the non-rapid eye movement (NREM) slow wave activity was inversely linked with tauopathy, suggesting that changes in NREM slow wave activity could help us detect tau pathology and cognitive impairment before or during the very early stages of symptomatic AD (69).

The pathogenesis of sleep disturbances and AD is not limited to pathological aspects alone; neuroinflammation is also a possible mechanism that connects these two issues (70). Abnormal sleep-wake cycles boost the synthesis of microglia Iba-1 and astrocyte glial fibrillary acidic protein and increase microglia-mediated proinflammatory release, further promoting the aggregation of Aβ and tau (71).

Besides these processes, the glymphatic system contributes significantly to the association between sleep and AD. The glymphatic system is a fast exchange and flow mechanism between Cerebrospinal fluid (CSF) and brain interstitial fluid (ISF) that relies on aquaporin 4 (AQP4) on the end foot of astrocytes to remove metabolites and abnormal proteins in the brain (72). Notably, fluid transport in the glymphatic system modulates lymphatic clearance periodically since it occurs during sleep, prolonged wave sleep, and it is inhibited during waking (73). Sleep disruptions can impair the glymphatic system function, resulting in Aβ and tau accumulation (66).

### 5.3 Chronic stress

Evidence from both animal and human studies has demonstrated that chronic stress exposure detrimentally affects cognitive processes in many ways (74). Animal studies have shown that chronic stress and higher levels of glucocorticoids lead to structural remodeling in brain regions such as the prefrontal cortex and the hippocampus (74). This remodeling includes neuronal atrophy (75), dendritic shortening and spine loss (76, 77), and the suppression of neurogenesis (78). These changes ultimately result in cognitive impairments (79, 80). In humans, chronic stress experienced during early life or adulthood has a significant impact on cognitive skills and the development of distinct psychopathologies. However, individual differences indicate that different factors like sex and genetic composition may also play an essential role in the development of stress-related mental health issues, including MCI and AD (81).

Experimental evidence suggests that chronic stress and glucocorticoids play a dual role in the development of AD (74). Their key function in developing the disease is indicated by their ability to directly induce Tau hyperphosphorylation in rats' prefrontal cortex and hippocampus (82, 83). In addition, external glucocorticoids enhance the capacity of centrally administered Aβ to trigger excessive phosphorylation of Tau epitopes linked to AD, as well as the accumulation of Tau in the cytoplasm, and previous exposure to stress exacerbates the biochemical and behavioral consequences of glucocorticoids in animals infused with Aβ (82, 83). Therefore, long-term exposure to stress and glucocorticoids may have a gradual effect on the development and advancement of AD pathology (82).

### 5.4 Physical inactivity

Physically active individuals have a decreased prevalence and incidence of cognitive deficits and dementia, including AD (84–87). A meta-analysis of 16 prospective studies involving 163,000 non-psychotic participants indicated a 28 and 45% reduction in overall dementia and AD among those who were physically active compared to those who were less active, respectively (88).

Physical activity elicits several anatomic, cellular, and molecular modifications in the brain through the activation of a cascade of cellular and molecular processes that advance various physiologic phenomena, such as neurogenesis, synaptogenesis, angiogenesis, and stimulation of neurotrophic factors, which play a significant role in improving memory, learning, and brain plasticity (89–94). Normally, a dementia-free elderly will experience an annual hippocampal shrinkage of 1–2% (95). It has been demonstrated that moderate intensity exercise training can significantly reverse the shrinkage process of the hippocampal volume (95). A 1-year aerobic exercise intervention resulted in a 2% increase in hippocampal volume and neutralized the normal decline that occurs with aging (95), and older adults with life-long routine exercise have larger brain volume and enhanced executive function than those who are physically inactive (96). Several studies have also discovered an inverse association between the levels of physical activity and the plasma and brain Aβ load in the elderly without cognitive disorders (97, 98). Moreover, a 6-month aerobic exercise intervention in individuals with MCI resulted in a 24% reduction in the plasma Aβ levels when compared to the levels found in the control group (99). These findings, along with those of several preclinical studies on AD models (100–103), suggest that exercise may significantly regulate Aβ turnover and decrease tau phosphorylation (104).

Physical activity has been shown to have a positive effect on improving cognitive symptoms (105). AD patients who participated in a year-long moderate exercise program had a slower decline in their ability to perform activities of daily living and ameliorated their physical impairment (106, 107). Additionally, Aerobic exercise has been shown in some other studies to improve memory performance and cognitive function in aging, MCI, and AD patients (99, 108, 109).

Therefore, recent evidence suggests that physical activity strongly protects AD development, alleviates some AD-related clinical signs and symptoms, and slows disease progression (105). However, it has yet to determine the optimal duration of the activity, the type and intensity of the exercise, and when in a person's life it should transpire in order to optimize the potential protective effects (110).

### 5.5 Excessive alcohol consumption

The epidemiological data concerning the association between alcohol and cognitive decline and AD-type neurodegeneration have generated significant controversy, primarily due to the inconsistent and variable measurement parameters of both alcohol consumption and AD (111). However, a discernible trend has emerged, as indicated by epidemiological investigations, which propose that moderate alcohol intake diminishes the incidence of AD (112–114). In contrast, heavy alcohol consumption increases dementia and impairs cognitive and executive functioning (113, 115). Acetylcholine release in the hippocampus region, which improves learning and memory, is the most likely mechanism implicated in the protective effect of low alcohol consumption on cognitive impairment (116). Another proposed mechanism could be considered as reducing cardiovascular risk factors such as platelet aggregation or serum lipid profile (116). Also, alcoholic beverages, particularly red wine, contain polyphenols such as quercetin, morin, tannins, and resveratrol, which can inhibit amyloid aggregation and reduce oxidative stress, inflammation, and balance of protein homeostasis, all of which play essential roles in AD pathology (117). However, whether low alcohol consumption protects against AD is still debated (111) due to additional confounding factors such as the impact of social contact on alcohol drinking and disparities in alcohol metabolism (118).

In contrast, there is a greater degree of consensus in the literature regarding the impact of heavy alcohol consumption on the risk of developing AD and other types of dementia (118). Several studies have found a positive association between heavy alcohol consumption and risk of AD and dementia (114, 115, 119, 120); however, this has not been universally observed (119). Heavy alcohol consumption promotes a decline in cognitive performance comparable to that observed in AD (121). Loss of cholinergic neurons detected in AD patients, as well as hippocampal atrophy (122), have also been discovered in individuals consuming ethanol (123, 124), establishing a link between heavy alcohol consumption and cognitive decline, which may ultimately lead to the development of AD. A potential mechanism suggested for alcohol to induce AD is through the reduction of glymphatic function (125). The glymphatic system is crucial for the removal of brain waste, including Aβ (105). Since alcohol impairs glymphatic function, heavy drinking may lead to Aβ accumulation by decreasing its clearance, precipitating the cognitive dysfunctions observed in AD (105). In addition to the potential link to trigger dementia, alcohol abstinence after AD diagnosis appears to ameliorate the initial cognitive impairment noted (126). Also, it suggests that heavy alcohol consumption not only raises the risk of developing AD but also exacerbates its progression (115).

Overall, although numerous studies have investigated the effects of alcohol intake on AD, controversies still remain, mainly due to the heterogeneity that exists between the studies regarding the age of participants, pattern, and duration of alcohol consumption, types of alcoholic drinks consumed, and underlying medical conditions and comorbidities (118).

### 5.6 Obesity

Several studies have concluded that midlife obesity, as evaluated by anthropometric measures such as BMI or the waist-to-hip ratio (WHR), is associated with an elevated risk of late-life AD and dementia in general, regardless of other risk factors (127–133). However, most of these studies have also agreed that there is a reverse causality impact, with BMI decreasing in the years before dementia onset (134). These studies demonstrated that the association between BMI and dementia is likely to be due to two distinct processes: a harmful effect of higher BMI, which is visible in long follow-up, and a reverse-causation effect that makes a higher BMI appear protective when the follow-up duration is short (134). In a prospective cohort study, the HR for AD was 0.89 (95% CI 0.81–0.98) among those with high late-life BMI; however, there was 20% increased risk of AD (95% CI 1.09–1.33) among those with greater BMI decrease from midlife to late-life (131). Moreover, another study found that HRs per 5-Kg/m^2^ rise in BMI for dementia were 0.71 (95% CI = 0.66–0.77), 0.94 (0.89–0.99), and 1.16 (1.05–1.27) when BMI was measured 10, 10–20, and >20 years before dementia diagnosis (133). It has been claimed that this “obesity paradox” or weight loss just before and during the clinical phase of dementia is linked to an increase in energy expenditure and hypothalamic dysfunction (134).

### 5.7 Poor diet

Low nutritional quality is strongly associated with cognitive impairment and the development of AD (135). Additionally, Protein-calorie malnutrition increases the risk of mortality in AD patients (136). Consumption of refined carbohydrates or a high glycemic index diet can lead to increased Aβ peptide accumulation in the brain (137). This impact is even significantly worse in APOE-ε4 carriers (137). However, the exact mechanisms of this association are still not being unraveled (138, 139). A western dietary pattern increases the risk of AD by promoting inflammation, making changes in metabolism, and decreasing cerebral perfusion, all of which impair cognition (140). On the contrary, adherence to the Mediterranean diet is associated with a lower risk of developing AD (137). The Mediterranean diet, rich in fruits, vegetables, fish, and whole-grain cereals, has been associated with better cognitive outcomes, increased gray matter volume (141–143), and a lower risk of developing MCI and AD, and of converting from MCI to AD (144, 145).

While it is clear that adherence to the Mediterranean diet improves cardiovascular health and protects against cerebrovascular diseases (146, 147), the protective association between Mediterranean diet and AD does not appear to be mediated by its effects on stroke or vascular risk factors (148). A 3-year serial amyloid and FDG-PET imaging study found that middle-aged cognitively healthy adults who followed a Mediterranean-style diet showed lower amyloid plaque deposition and cerebral hypometabolism, regardless of APOE genotype or vascular risk factors (149). This finding supports the protective effect of the Mediterranean diet against AD (134).

A ketogenic diet may also be effective in preventing AD, as it reduces oxidative stress, inflammation, and the harmful consequences of altered glucose metabolism in the brain. Moreover, it can improve attention, verbal memory, and overall cognitive performance (137).

Furthermore, appropriate omega-3 polyunsaturated fatty acids, particularly EPA and DHA, are related to slower cognitive decline and a lower risk of AD (137). As a result, it is critical to counsel people at risk of developing of AD to consume sources of omega-3 such as seeds, fish, nuts, fish, and vegetable oils (150, 151).

### 5.8 Social isolation

Social isolation, traditionally known for its detrimental effects on both mental and physical wellbeing, has been shown to deteriorate cognitive function, accelerate psychological aspects of aging, and increase the likelihood of developing AD and overall dementia (152–155). However, it should be highlighted that this relationship is bidirectional, as early symptoms of AD may also lead the person to feel socially isolated (152). Social isolation has two aspects: objective, which includes the lack of social networks and reduced engagement in social activities, and perceived, which encompasses feelings of loneliness and inadequate social support (156). Evidence suggests a strong correlation between these aspects and cognitive decline (152).

Social isolation may influence AD development via psychological and neurobiological pathways (152). According to the cognitive reserve hypothesis, the brain uses pre-existing cognitive processes or activates compensating pathways to deal with brain damage (157). Lower education and occupational attainment and decreased participation in leisure activities have been associated with a lower cognitive reserve (157) and an increased risk of developing AD (158, 159). Social isolation reduces cognitive stimulation, potentially leading to lower cognitive reserve, thereby increasing the risk of cognitive decline (160).

## 6 Medical conditions

Several studies have investigated the link between AD and other health conditions. Numerous studies have implied the link between cardiovascular disease (CVD) and dementia (161, 162). Follow-up of individuals with different levels of cognitive impairment, ranging from normal cognitive function to impaired cognition as dementia level, shows that adverse vascular risk, as measured by the Framingham cardiovascular risk profile (FCRP), is associated with the decline of multiple domains of the cognitive function, and progression of AD (161–165). In a prospective cohort of 3,602 patients without dementia, during a follow-up of 5.4 years, participants who had CVD or peripheral artery disease showed a 30 and 140% higher risk, respectively, for progression to AD (166). Diabetes mellitus (DM) is not only a major risk factor for CVD, but also has a significant role in developing AD independently. Previous large-scale cohorts indicate that patients with diabetes mellitus (DM) or even prediabetic individuals are faced with an increased risk of cognitive deterioration, dementia, and AD (167, 168). Consistent with these findings, population-based data has revealed DM is considered to be responsible for about 2.9% of new cases of AD worldwide and a 46% excessive risk for projecting AD (169). A cohort study on patients with type 1 DM concluded that long-term exposure to poorly controlled hyperglycemia (i.e., HbA1C ≥ 8%) is correlated with a higher risk of developing dementia (170). On the other hand, in a prospective cohort of randomly selected 416 patients with a long history of type 1 DM from the Epidemiology of Diabetes Interventions and Complications (EDIC) and 99 sociodemographically matched control participants, pattern analysis of the participants' brain magnetic resonance imaging (MRI) unveiled that type 1 DM is correlated with brain atrophy in the thalamus, putamen, superior and middle frontal gyrus, and superior temporal gyrus, consistent with a nearly 6 years older brain age. However, the association of type 1 DM and patterns consistent with early AD-related neurodegeneration was not confirmed (171).

A recently published meta-analysis of incidence and cohort studies suggested depression is associated with 82% extra risk for the development of dementia (172). The results of prior-published meta-analyses show that late-life depression augments the risk of all-cause dementia (173, 174). Population-based data represents a 65% excessive risk attributed to depression for developing AD, yielding 7.9% cases of the disease (169). Longitudinal follow-ups of different cohorts of patients with Parkinson's disease (PD) signify the variable incidence of newly diagnosed dementia among this population, changing from 30 to 112.5 per every 1,000 person-years (175–183). Previous cohort studies exhibit a multiplied risk, even near to 6-fold greater, in patients with PD compared to the control population without PD (177, 180, 181, 184). A more recent *post-hoc* analysis on patients in PARKreg registry showed an approximate 4-fold increased risk associated with PD for progression to dementia, compared to sex- and age-matched individuals without PD (185). However, a neuropsychological comparison of patients with dementia associated with PD and individuals with AD has revealed comparable memory impairment, while different cognitive profiles regard orientation and attention tests (186).

Prior investigations on the association of hypothyroidism with dementia have inconsistent results. Retrospective assessment of patients with AD illustrates the higher prevalence of hypothyroidism in this population compared to those without AD (187, 188). Similarly, analysis of data from different case-control studies has confirmed the increased risk of progression to AD among patients with hypothyroidism (189, 190). Assessments of patients derived from two prospective cohorts (DNPR and OPENTHYRO) suggested the increased risk for projection to dementia among patients with hypothyroidism. They claimed that the risk of dementia increases by 12% every 6 months of elevated TSH level (191). Longitudinal prospective follow-up of 1,836 persons with intact cognitive profiles derived from the Framingham cohort revealed that during a mean follow-up of 12.7 years, although elevated levels of thyroid stimulating hormone were associated with 115% higher risk for development of AD in the population of women, the risk among men was similar with different levels of thyroid stimulating hormone (192). However, some other investigations claim that hypothyroidism, either as clinical or as subclinical, is not associated with the incidence of cognitive deficit (193). Interestingly, available meta-analyses in this entity have proved controversial results. A meta-analysis of 15 different longitudinal and cross-sectional studies concluded that both clinical and subclinical hypothyroidism are accompanied by an increased risk of dementia (194). On the other hand, another meta-analysis of eight studies with a pooled population of 1,092,025 patients conversely showed that after adjustment for vascular risk factors, hypothyroidism is even associated with reduced risk for cognitive deterioration (195).

The role of chronic kidney disease (CKD) in progression to AD has remained contradictory. Cross-sectional data unveiled that alteration of cognitive function is concordant with the severity of the renal impairment, as patients with end-stage renal disease (ESRD or stage 5 of CKD) have acquired worse scores on different cognitive tests compared to those with stage 3 or 4 of CKD (196). In cognitive evaluation of 825 patients derived from the CRIC study (a prospective cohort for assessment of risk factors and mechanisms of progression of CKD and CVD) with age more than 55 years old and different levels of baseline kidney function, lower estimated glomerular filtration rate (eGFR) at the time of evaluation was accompanied with worse scores in most cognitive domains (197). In a prospective cohort of 7,839 patients, a fast decline in estimated glomerular filtration rate (eGFR) (i.e., > 4 mL/min/1.73 m^2^/year) was associated with more than 5-fold higher risk for developing dementia with vascular component and greater cognitive deterioration compared to those with lower rate of eGFR decline, during the first 4 years of follow-up. However, presence of CKD at the baseline did not correlate with the incidence of dementia or cognitive decline in the 7-year follow-up (198). More recent data has shown debatable results. A longitudinal follow-up study on the Korean National Health Screening Cohort concluded that CKD is associated with an increased risk of AD (199). Unexpectedly, another prospective cohort of 205,622 patients stratified based on the baseline eGFR revealed a remarkably higher risk for AD among patients belonging to strata with baseline eGFR of 30–89 mL/min/m^2^, while patients with baseline eGFR of < 30 mL/min/m^2^ showed a significantly higher risk for developing AD only after further adjustment for medications, and to a lower extent compared with baseline eGFR of 30–89 mL/min/m^2^. However, the risk of all-cause dementia was consistent with the level of renal impairment, as worse baseline eGFR was associated with a higher risk of projecting all-cause dementia (200). In contrast, a 17-year prospective follow-up of a cohort of 6,256 patients unveiled that renal impairment (defined as eGFR < 60 mL/min/m^2^) increases the risk for neither all-cause dementia nor AD, despite its strong association with an increase in the level of dementia-related blood biomarkers (201).

## 7 Symptoms of Alzheimer's disease

AD is a common, invaluable, and sporadic degenerative brain disorder. It is not just a decrease in brain volume and weight but a progressive, extensive atrophy of the cerebrum. This condition involves a significant loss of neural cells, especially in the hippocampus and medial temporal lobes, confirmed through pathological tests (202). Additionally, clinical evaluations must be completed before accepting an AD diagnosis and differentiating it from mild cognitive impairment (MCI) syndrome. These diagnostic criteria consist of detecting dementia in patients older than 40 years using each of the clinical questionnaires of the Mini-Mental Scale (MMSE), Blessed Dementia Rating Scale (BDRS), or Alzheimer Disease Status Assessment Scale (ADAS) which affects at least 2 areas of cognitive functions either memory, language, perception, visuospatial, psychosocial, or motor skills (203). However, MCI is defined as the presence of cognitive abnormalities in at least 1 field that does not interfere with routine daily activities (204). Here we separately discuss each of presenting symptoms in the early and late stages of AD.

It's been mentioned in the literature that the clinical course of the disease could vary from usually more than 5 years up to 20 years after its establishment using CSF analysis and neuroimaging findings (205), but further investigations revealed that the onset of the molecular course of this ominous illness could be much longer as in the familial AD which 15 years or longer has been indicated (206). More often, the beginning of the mental symptoms, prominently memory deficit as the most common and most important one, occurs insidiously. Still, occasionally, a febrile episode, traumatic brain injury, surgery, or commencement of a new drug exploit the underlying process. Discussing the course of AD, lacking a plateau condition or cessation of the disease progression has been declared, and the absence of symptom deterioration for a long period infers other diagnoses such as frontotemporal dementia, hydrocephalus, temporoparietal embolic infarcts, or Binswanger disease (207). Eventually, the debilitated and incapacitated patient dies due to conditions related to worsening cognitive disabilities, severe weakness, and being bedridden, such as cardiovascular or pulmonary dysfunctions.

Memory impairment, as a commonly manifested symptom in ~90% of AD patients, primarily affects short-term and episodic memory, even if it presents as the only symptom in patients for several years. However, immediate or working memory, which is relatively proportionate to the patient's attention, often remains intact (208). On the other side, despite the severe demolition of long-term memory in late-stage AD patients, no significant changes could be detected in early-diagnosed patients. Forgotten appointments, misplacing household items, frequently repeating the same questions, and having inconclusive and imperfect discussions are some of the common symptom (209, 210).

Problems in language usually occur when memory dysfunction reaches a prominent level. As a result of being unable to reach the necessary word, the patient stutters and uses words stereotypically due to decreased word variety (211). The same problem prevents from proper writing, and the spelling precision diminishes. These linguistic abnormalities are first hardly apparent but become more noticeable as the illness worsens, eventually leading to imperceptible discussions. There is a lack of whole sentences after several years of disease, prolonged phases of pauses, and word searches that disrupt ordinary discourse. Their struggle could lead to substituting other words or comprising discontinued sentences. In later stages, echolalia and anomic aphasia would ensue in the clinical course (212). Despite the lack of isolated Wernicke's or Broca's aphasia, which raises a suspicion of cerebral ischemia, deterioration of anomic aphasia in the receptive or executive area could be accepted in the disease course. Dysnomia in AD patients could be assessed using a test in which AD patients are asked to declare as many names as possible related to a specific category in 1 min (213). Less than eight items in each mentioned category strengthen AD disease diagnosis. Seldom, debilitated language function presents as the first manifestation of the disease called the logopenic variant of primary progressive aphasia (lvPPA) (214).

The calculation could also be affected, resulting in dyscalculia, which is usually demonstrated by faulty account management, incorrect money changes, and aggravated form disability in even calculating the most straightforward arithmetic (215). Altered decision-making also could be detected but it has been mentioned that repeatedly established work habits by business experts are not drastically affected at least during the early stages (216).

Disorientation to time and place is an early symptom that is observed in Alzheimer's individuals. It means the inability of memory to update information about time and place (217, 218). It may be experienced while entering new places or relocating to a new house, but it also can be experienced in daily and routine environments (219). Individuals became confused about seasons and dates and even recognized day or night. Visuospatial disorientation is a related condition that could be attributed to parieto-occipital dysfunction and may be detected in a variable proportion of AD patients (220). Its pure occurrence, which may induce complete cortical blindness, is defined as posterior cortical atrophy (221). Defective recognition of similar faces (called prosopagnosia), difficulties in dressing up or parking a car, inability to distinguish the correct way back home or giving directions to other people in despite of a bunch of familiar clues, feeble interpretation of maps, or even inaccurate right or left side detection.

Functional and executive disturbances, which can be considered the most disabling aspect in AD patients, partly resulting from the underlying decline in cognitive and neuropsychological functions. Further, concomitant restrictions in working and learning competencies, including defective task coordination and inability to perform complicated instructions, aggravate self-esteem and result in dissociation from the community, depression, resignment, financial problems, and initiation of a vicious circle (222, 223). Early incapacitation implementing complex orders exacerbates, and doing easier, previously automatic activities like driving get troublesome. Locomotor abnormalities may appear early, with walking speed being the most commonly altered parameter. However, Gras et al. (224) reported that crude unsteady balance, sluggish gait speed, increased stance time, and shorter step length could be detected in AD individuals. Advanced stages could be presented by fine tremors and rigidity resembling Parkinson's disease (225). Later in the disease course, primary reflexes of grasping and sucking reappear, and the patient loses his continence of sphincters which accompanying moving and standing impairments, bed rest, and subsequent vegetative conditions ensue, which necessitates persistent patient care (226).

Most AD patients have behavioral impairment throughout their illnesses, which predominates in later stages, while other cognitive or functional areas are mild to severely affected. Incautious actions or decisions are made without any attention to feedback. The patient's affect becomes harsher as he becomes more self-centered, rejecting to see old friends or family members and uncaring about how others see him. Depressive symptoms, including reduced or heightened food intake, irritability or inertia, disinhibition, sleeplessness or hypersomnia, and repetitive or obsessive behaviors, may occur. Although sometimes a ravenous hunger sets in, commonly eating is disregarded, leading to a slow but steady weight loss. Neglected self-care and a wildly disheveled general outlook are also frequently reported (227). Anhedonia and apathy are often encountered owing to the frontal lobe involvement with associated lassitude in starting a novel task, lack of consistency, and deficient motivation, but anxieties and sometimes phobias can occur with an aversion to being alone (228). A patient's sexual misdeeds or attraction to a younger person may shock friends and family yet threaten to destabilize an otherwise successful marriage. Paranoid delusions, sometimes accompanied by hallucinations, could be other manifestations of AD patients. The patient's children may be accused of stealing from him or his old wife may be suspected of having an extramarital infidelity (229).

## 8 Symptoms and signs assessed during a physical examination for Alzheimer's disease

Since AD affects different areas of the central nervous system, signs and symptoms of this disease vary greatly. So, it is essential to do a proper physical examination (230).

### 8.1 Memory loss and cognitive impairment

Memory loss and cognitive impairment are key indicators of AD. The severity of cognitive impairment correlates best with the burden of neocortical neurofibrillary tangles, which are a hallmark of AD (231). Memory loss is among the first symptoms reported by patients suffering from AD. Disease pathology interferes with the formation of memories from the molecular level to the framework of neural networks (232). Prospective studies indicate an early decline in executive functions and episodic and semantic memory processing in the predementia period (233). Low performance in learning and memory functions may be the earliest cognitive manifestations of preclinical AD (234). Also, asymmetry in cognitive abilities may occur in the preclinical phase of AD (235). Memory test scores reflecting the difference between immediate and delayed recall are important in identifying which individuals with recent cognitive changes will progress to where they meet the criteria for Alzheimer's over time (236). Episodic memory decline is the most salient cognitive function that correlates with high levels of amyloid deposition and hypoconnectivity across large-scale brain networks (233).

### 8.2 Changes in behavior or personality

Personality changes occur alongside or sometimes before cognitive symptoms (237, 238). A systematic review showed increased neuroticism and decreased extraversion, openness, agreeableness, and conscientiousness (239, 240). A significant change from positive to negative characteristics in personality is detected after the beginning of AD (241).

### 8.3 Loss of coordination or balance

Balance and mobility impairments are linearly correlated with cognitive function and can be observed at the beginning of the subjective cognitive decline stage (242). For instance, an impaired one-leg balance test indicates a higher cognitive decline (243). Standing balance in people with AD is significantly impaired across a range of static and dynamic balance conditions. Activity level, mobility measures, turning, and dual tasks are also impaired (244).

### 8.4 Abnormal gait

Gait parameters can differentiate between healthy elderly, mild cognitive impairment, and AD patients (245). Gait analysis can potentially contribute to the diagnosis and prognosis of cognitive impairment (246). Dual-tasking frequently results in gait disturbances, which may affect gait stability and increase the risk of falling (247). Overall, gait analysis can be a useful tool for identifying AD and monitoring its progression.

### 8.5 Loss of sensation or reflexes

Loss of sensation may be an indicator of AD. AD patients have a sensory gating deficit, which may result from cholinergic dysfunction and alpha-7 nicotinic receptor loss (248). They also have an impaired sensation of pain, which means their pain tolerance is increased, and their reaction to pain is markedly diminished (249, 250). Loss of reflexes may be a relevant sign in the diagnosis of AD. The palmomental reflex is more frequent in AD patients even 2 years before diagnosing AD (251). Also the prevalence of primitive reflexes is correlated with dementia severity (252).

### 8.6 Abnormal eye movements or vision

Abnormal eye movement or vision can indicate AD. Eye movements, including saccades and smooth pursuit, seem to be slower and less accurate as a result of the disease. Even subtle functions, such as microsaccades and pupillary dilations, are changed by AD (253). A decrease in concentration is one of the symptoms that comes with cognitive decline, which causes frequent eye and facial movements. These differences can be used to screen AD symptoms quickly, even with camera recordings (254).

Among the earliest symptoms seen in AD patients are visual impairments, particularly loss of contrast and color sensitivity, limited visual field, compromised visual attention, reduced stereopsis, and impaired object and face recognition (255–259). Anatomical changes within the eye have been detected before signs of cognitive impairment and memory loss appear (257). All parts of the visual system, including the optic nerve and the retina, may be affected in AD (260). According to a systematic review, the peripapillary retinal nerve fiber layer (RNFL) and macular thickness are significantly decreased in AD patients (261).

### 8.7 Tremors or other involuntary movements

Among motor signs during AD, tremor is the least frequent and appears independent of disease progression (262–266). As AD progresses, the frequency of motor signs like speech/facial expression, rigidity, posture/gait, and bradykinesia also increase (266, 267).

As reviewed above, the physical examination results can help us diagnose AD, and in some cases, even better and sooner. Other diagnostic assessments are used with physical examination and can either confirm our diagnosis or rule out other diseases (268–270).

AD is a slow and progressive disorder, and it's been difficult for clinicians to identify the transitions from the asymptomatic phase to the symptomatic predementia and dementia phase (270). In cases of mild cognitive impairment (MCI), physical examination alone can't indicate the progression to AD, and here, the biomarkers are a great help in identifying this matter (271, 272). Additionally, all cognitive tests are influenced by some unchangeable variables like age, education, and cultural variations between individuals (270).

## 9 Laboratory tests for Alzheimer's disease diagnosis

### 9.1 Blood tests

#### 9.1.1 Amyloid beta and tau protein levels

Since the discovery of APOE ε4 around 25 years ago, researchers have shown much enthusiasm toward the prospective development of a blood test for AD. Yet, the use of blood tests for AD has not been implemented in clinical settings (273).

Recent research has shown that the Aβ42/40 ratio, as determined in plasma, can distinguish between individuals without cognitive impairment and those with AD. However, it is worth noting that this particular technology is not widely available in most healthcare facilities (274, 275). The blood plasma levels of Aβ42 exhibit changes similar to those previously documented in cerebrospinal fluid (CSF), suggesting a potential pathway to transit Aβ between the blood and the brain through the blood-brain barrier (BBB). The stability and sensitivity of plasma Aβ values indicate that this screening test may be helpful in detecting central nervous system (CNS) amyloidosis (275).

Another non-invasive diagnostic and predictive biomarker for AD is plasma P-tau181, which may be helpful in clinical practice and studies (276). Plasma P-tau181 was shown to be elevated in preclinical AD and further elevated in the MCI and dementia phases in research by Janelidze et al. They demonstrated that Plasma P-tau181 had accuracy comparable to Tau PET and CSF P-tau181, distinguished AD dementia from non-AD neurodegenerative illnesses, and identified AD neuropathology in a cohort with autopsy confirmation (276). When taken as a whole, plasma P-tau181 may be a practical tool for identifying people who need further diagnostic testing (277).

### 9.2 Inflammatory markers

Intracellular alterations in individuals with AD may be detected early by using inflammatory markers (278). Interleukin-6, 12, and 18, as well as tumor necrosis factor (TNF) and transforming growth factor (TGF), have been shown to be present in increased concentrations in the blood of AD patients (279).

Additionally, glial cells may become pro-inflammatory if stimulated for an extended period, as in AD (280). Microglia and astrocytes express the inflammatory marker chitinase-3-like protein 1 (also known as YKL-40). Astrocytes close to Aβ plaques express YKL-40 in AD, and tau pathology positively correlates with this expression (281, 282). Another biomarker connected to AD's progression is glial fibrillary acidic protein (GFAP). Prins et al.'s cross-sectional investigation showed that preclinical AD patients had higher plasma levels of GFAP than healthy controls (283).

### 9.3 Cerebrospinal fluid analysis

#### 9.3.1 Aβ and tau protein levels

It was shown in 1992 that Aβ is secreted into the CSF (284). Numerous articles have confirmed the reduction in CSF A42 in AD dementia; a meta-analysis of 131 research revealed extremely consistent results, with a mean fold change for CSF A42 of 0.56 compared with older individuals with normal cognitive function (285).

After a long-standing mystery, a 2003 autopsy investigation linked low postmortem ventricular CSF Aβ42 levels to high plaque numbers (286), which is consistent with the pathophysiological theory that the hydrophobic peptide aggregates and becomes sequestered in plaques in AD, leaving less of it to be secreted into the extracellular space and the CSF, leading to lower CSF levels of A-42 (287). Other Aβ species than Aβ42 are discovered in human CSF, with Aβ1-40 being the most prevalent and having around ten times the amount of Aβ42 (288, 289). Numerous investigations have shown that while CSF Aβ40 levels remain relatively unchanged in Alzheimer's disease (AD), the CSF Aβ42/Aβ40 ratio is more effective than CSF Aβ42 alone in detecting AD (285, 290–292). Therefore, in a clinical environment, the CSF Aβ42/Aβ40 ratio could have diagnostic importance (293).

Another biomarker for AD is tau, a microtubule-associated protein found in neuronal axons with six distinct human isoforms (294, 295). Tau is hyperphosphorylated and is transported from axons to the somatodendritic compartment in many AD neurons, where it is misfolded and clumps.

According to research, axonal loss and neuronal death cause the intracellular protein tau to be released, increasing the tau level in the CSF in AD (296, 297). CSF T-tau levels are dynamic after acute brain trauma, rising within days after the lesion and remaining high for weeks until returning to normal (298, 299). However, in some pure tauopathies, CSF tau is not raised despite severe neurodegeneration and tau pathology (300, 301).

#### 9.3.2 Phosphorylated tau levels

Through the use of antibodies that target distinct phosphorylated patterns within the amino acid sequence of tau (referred to as p-tau), some isoforms of p-tau (namely p-tau181,−199, and−231) have been identified as exhibiting a greater association with AD. P-tau231 and P-tau181 may be utilized to differentiate AD from control groups and even from FTD, dementia with Lewy bodies (DLB), vascular dementia (VaD), and major depressive disorder (287, 300, 302–305). There aren't many studies directly evaluating various CSF Ptau tests as AD biomarkers, although one study found that P-Tau181, P-Tau199, and Pau231 levels in CSF closely correlate and perform similarly to distinguish AD from other neurodegenerative diseases and non-demented individuals (304). Moreover, p-tau217 exhibits significant potential as a biomarker in Alzheimer's disease, notably due to its marked increase in levels ~21 years prior to the onset of symptoms, which closely correlates with the accumulation of amyloid-β. As the disease progresses, both p-tau217 and p-tau181 levels decline significantly near the onset of symptoms, while total tau levels and unphosphorylated isoforms (such as Tau217 and Tau181) continue to rise (306). This dynamic suggests that the reduction in phosphorylation ratios is not solely attributable to a rise in unphosphorylated peptides or a decline in overall tau protein levels (306). Instead, it signifies a complex interaction of tau phosphorylation that corresponds with the advancement of Alzheimer's pathology. Recent research highlights the necessity for additional validation of these tests to secure regulatory approval, which entails standardizing measurement protocols and conducting longitudinal studies across varied populations. The potential for early detection and monitoring of Tau217 ultimately underscores its significance in understanding Alzheimer's disease and guiding future treatment approaches (306).

In individuals with moderate cognitive impairment (MCI) (307) and with neocortical NFT pathology in AD (308), CSF p-tau levels are correlated with cognitive deterioration. Additionally, t-tau and p-tau both predict the rate of cognitive decline in various stages of AD (309–311). The concentration of p-tau231 decreases over time from mild to moderate AD (312). It significantly correlates at baseline with the rate of hippocampal atrophy in mild to moderate AD (313), indicating structural disease progression. In a European multi-center trial, CSF p-tau consistently predicted AD in people with MCI with high accuracy (80%) over 1.5 years, a period that was both brief and clinically meaningful (314).

#### 9.3.3 Neurofilament light chain levels

Intermediate filaments, known as neurofilaments, are found in neurons and are especially prevalent in axons (315). One of the four components of neurofilaments in the CNS is called neurofilament light (Nf-L). Neurofilaments have a crucial role in facilitating the radial expansion of axons throughout the developmental process, providing structural stability, and facilitating the conduction of electrical signals (315). Several neurological disorders, including AD, exhibit abnormal neurofilament aggregation and other changes (315–317).

The NF-L subunit's involvement as a marker of axonal damage is supported by the knowledge that CSF levels of the subunit are elevated in a number of neurodegenerative disorders (318–320). As compared to controls, AD and MCI patients have been demonstrated to have increased CSF NF-L levels (320–323). According to Olsson et al.'s meta-analysis (285), which included data from nine AD cohorts and eight control cohorts, CSF NF-L had a significant effect size for discriminating between AD patients and controls. CSF NF-L is correlated with brain atrophy (323, 324), but it doesn't seem to be particular to AD, since levels are raised in other neurodegenerative disorders, possibly reflecting non-specific axonal damage (324–326).

### 9.4 Novel recently developed probable diagnostic biomarkers

To identify reliable biomarkers in AD, researchers have focused on measuring Aβ and tau in CSF and blood, as well as using neuroimaging techniques such as FDG-PET, Pittsburgh compound B-PET, and MRI to measure hippocampal volume (327, 328). It is now advised to use a multi-marker strategy since none of these instruments can aid in diagnosing independently. As a result, the availability of non-invasive, readily accessible biomarkers is very valuable. Researchers have suggested various new biomarkers, such as inflammatory markers, fluorescent nanoparticles, and microRNAs.

According to a recent systematic review, a total of 10 microRNAs (miRNAs) have a substantially altered expression in individuals with AD compared to normal subjects, as evidenced by at least two independent investigations. Furthermore, the research highlights that over thirty miRNAs can differentiate between two distinct neurodegenerative disorders (329). AD patients ' extracellular fluid (ECF) and CSF were reported to have higher levels of some miRNAs, including pro-inflammatory miRNA-9, miRNA-125b, miRNA-146a, and miRNA-155 (330).

One further potential biomarker for the diagnosis of AD is fluorescent nanoparticle kits. The outcomes of efforts to develop these kits include the AD Diagnostic Kit WO2002/088706, which uses glutamine synthetase as an indicator, and WO2010/144634, which detects DNA methylation as an epigenetic marker to diagnose AD (331). Furthermore, Park et al. confirmed that the fluorescent nanoparticles they created and the WO2002/088706 kit would potentially give an early diagnosis of AD using plasma from AD patients (332).

Inflammatory biomarkers, including IL-6, CRP, and TNF-α, may be additional cutting-edge biomarkers for identifying AD due to the inflammatory character of the disease. However, a recent meta-analysis by Ng et al. found no difference in IL-6, CRP, and TNF levels between elderly with AD and controls (333).

## 10 Imaging tests for diagnosis of Alzheimer's disease

AD is a complex ailment mainly affecting memory and cognitive abilities. Diagnosing it early and accurately ensures timely treatment and patient care (334). Advanced imaging techniques assist physicians in confirming clinical suspicions, identifying those at risk, monitoring the effectiveness of potential treatments, differentiating Alzheimer's from other conditions, and evaluating the severity of the disease (335).

### 10.1 Magnetic resonance imaging

Magnetic resonance imaging (MRI) is a non-invasive imaging technique that uses strong magnetic fields to generate detailed brain images. In AD diagnosis, MRI primarily assesses brain structure, detects atrophy, and rules out other potential causes of cognitive impairment, such as tumors or vascular abnormalities (336, 337). MRI scans reveal brain atrophy, particularly in regions critical for memory formation (336, 337). Advanced MRI techniques can quantify microstructural changes and accurately measure brain volume loss, aiding early AD detection and monitoring disease progression (336, 337). However, it should not be relied upon alone in identifying evidence of AD due to its low accuracy (338). The sensitivity of MRI in detecting AD in patients with mild cognitive impairment is 73%, and the specificity is 71% (338).

### 10.2 Computed tomography

Computed tomography (CT) is another imaging modality used in Alzheimer's diagnosis, but less frequently than MRI (339). CT scans provide detailed cross-sectional brain images and can help rule out other pathologies that may mimic AD symptoms. CT scans can reveal structural abnormalities in the brain, such as ventricular enlargement or cortical atrophy, associated with AD (339). CT is often more readily available and quicker to perform than MRI, making it useful in specific clinical situations when MRI is contraindicated (336). In AD, microscopic histological changes are associated with focal atrophy in the medial temporal lobe region, including the hippocampus, reflecting the typical progression of neuropathology, which can be assessed *in vivo* using MRI (339, 340). Many studies have evaluated the diagnostic value of hippocampal atrophy for AD using MRI, with overall sensitivity and specificity estimated to be 85 and 88%, respectively (341).

### 10.3 Positron emission tomography

Positron emission tomography (PET) is a powerful imaging technique that provides functional information about the brain's metabolic and molecular processes. PET scans detect specific hallmarks of AD pathology, such as the accumulation of amyloid plaque and intracellular hyperphosphorylated tau neurofibrillary tangles, as well as changes in glucose metabolism (342, 343). Amyloid plaques are deposits of beta-amyloid protein that accumulate between nerve cells, while neurofibrillary tangles are twisted tau protein fibers that build up inside nerve cells (344). Although imaging techniques cannot directly visualize neurofibrillary tangles, they can infer their presence by detecting associated brain atrophy and functional abnormalities (345). As amyloid-targeting therapeutics emerge in the treatment of AD, amyloid PET scans are being used to determine patients' eligibility for clinical trials and to assess treatment outcomes. Three Amyloid-β radiotracers, namely ^18^F-florbetaben, ^18^F-florbetapir, and ^18^F-flutemetamol, are currently approved by the FDA for use in clinical practice (346). Moreover, the development of numerous second-generation tau PET tracers with enhanced binding properties has been achieved. These tracers include [18F]MK6240, [18F]PM-PBB3, [18F]RO948, [18F]PI-2620, [18F]JNJ311, and [18F]Genentech Tau Probe 1 (GTP1) (347, 348). Each of these radiotracers has its own set of procedural guidelines and acceptable interpretation techniques, but they all share the fundamental property of binding to white matter, not gray matter. Accordingly, abnormal scans will demonstrate evidence of diminished gray-white differentiation, outward radiotracer extension from the white matter to the cortical surface, or increased radiotracer uptake by gray matter compared to white matter (349). In addition, the FDA has authorized the use of the ^18^F-flortaucipir radiotracer to visualize the distribution and density of NFTs in patients with cognitive impairment who are being assessed for AD. Since changes in tau PET binding are correlated with cognitive symptoms, this radiotracer provides value not only for diagnosing AD but also for tracking the spatiotemporal progression of tau pathology in longitudinal investigations (350).

Additionally, PET measures glucose metabolism, allowing clinicians to assess brain function and detect regions with reduced metabolic activity, which is indicative of Alzheimer's-related neuronal dysfunction (342, 351). PET can be used to study brain metabolism and visualize glucose metabolism using the metabolic tracer ^18^F-fluorodeoxyglucose (FDG) (352, 353). PET scans with FDG or other radiotracers provide insights into regional changes in cerebral blood flow and glucose metabolism, highlighting areas of reduced neuronal activity (352, 353). In AD, hypometabolism in the temporal, parietal, and posterior cingular regions distinguishes AD patients from controls with good discriminatory power (354, 355). Recent studies show that amyloid PET brain imaging is highly sensitive and specific for neuropathologically confirmed AD, while FDG PET is highly sensitive and moderately specific (356).

In conclusion, MRI and CT scans reveal progressive brain atrophy, especially in regions associated with memory and cognition. PET imaging, using multiradiopharmaceutical agents, identifies the presence and distribution of amyloid plaques and intracellular hyperphosphorylated tau neurofibrillary tangles in the brain. As our understanding of Alzheimer's disease advances, imaging techniques are expected to become increasingly refined, providing more accurate and informative insights into this devastating condition.

## 11 Current treatments for Alzheimer's disease

### 11.1 Cholinesterase inhibitors

#### 11.1.1 Cholinesterase inhibitors mechanism of actions

In patients with advanced AD, specific neuronal pathways exhibit decreased excitability, leading to delayed neurotransmission. Cholinesterase inhibitors function to augment neurotransmission between neuronal synapses to ameliorate Alzheimer's symptomatology. These pharmacological agents are indicated for individuals with mild to moderate Alzheimer's manifestations (357). Previous research indicates that cholinesterase inhibitors engage with cholinergic receptors, influence sodium and potassium ion channels, and impact neurotransmitters' uptake, synthesis, and release. In essence, cholinesterase inhibitors exhibit multiple mechanisms of action, which could be beneficial when addressing intricate conditions like AD (358).

#### 11.1.2 Types of cholinesterase inhibitors

##### 11.1.2.1 Donepezil (Aricept)

Donepezil is the first-line medication for AD. There are several RCT studies on the effectiveness of Donepezil on AD. A meta-analysis of randomized controlled trials in which five RCTs with 2,974 patients were conducted showed that high-dose donepezil significantly improved cognitive function compared to standard-dose donepezil. The overall incidence of adverse events between the various doses was not observed; However, high-dose donepezil increased the risk of heart-related issues. In conclusion, while high-dose donepezil enhances cognitive function in moderate-to-severe AD patients, caution is advised for those with heart conditions (359).

A study by Xuemei Zhang assessed the effectiveness and safety of donepezil in treating patients with mild cognitive impairment. Analyzing 12 randomized controlled trials (RCTs) and five non-randomized controlled trials (CCTs), encompassing 2,847 patients revealed that donepezil significantly improved MMSE and MoCA scores. However, it didn't notably delay disease progression; the evidence quality was generally low. Concerning safety, donepezil increases the risk of side effects like nausea, vomiting, and diarrhea. In summary, while donepezil can somewhat enhance cognitive function in MCI patients, it doesn't significantly delay the disease's progression and may cause adverse reactions (360).

A meta-analysis study by Kazufumi Yoshida was conducted to develop a personalized prediction model for AD based on individual characteristics. A comprehensive study of 8 randomized trials with 3,156 participants trials compared the oral donepezil vs. placebo. It was revealed that donepezil is rather efficacious for cognitive function for most patients in contracts with placebo; however, using antipsychotic drugs at the study's onset might reduce donepezil's effectiveness. Despite the drug's potential benefits, larger studies with more variables and extended durations are essential (361).

##### 11.1.2.2 Rivastigmine (Exelon)

New drugs were developed during the late 20th century to control the progression of AD. Rivastigmine, introduced in 1985 and approved by the FDA in 1997, is used to treat mild to moderate dementia in Alzheimer's and Parkinson's patients (362). Recent research suggests rivastigmine also enhances gait stability, reducing fall risks for those with Parkinson's (363).

In a study by Jacqueline S Birks, 13 trials, ranging from 12 to 52 weeks, studied the efficacy of either orally (6–12 mg/day) or transdermally (9.5 mg/day) administered medication with a placebo. This analysis used data from seven trials encompassing 3,450 patients. After 26 weeks, patients on rivastigmine demonstrated improved cognitive function, daily living activities, and a better clinician's global impression, with three trials showing no difference from the placebo. In conclusion; Rivastigmine benefits individuals with mild to moderate AD. Compared to a placebo, it showed a slower decline in cognitive function and daily activities, although the improvements were minimal, and their clinical significance is uncertain. Rivastigmine also positively impacted clinician's global assessments. However, there was no noticeable difference in behavior changes. The studies' quality of evidence is moderate, with potential biases due to participant dropouts, and all research was industry-sponsored. Economic implications weren't covered in this review (364).

##### 11.1.2.3 Galantamine (Razadyne)

Galantamine, a natural tertiary alkaloid found in plants like *Galanthus nivalis*, was identified in the 1950s. Initially explored for neuropathic and paralytic conditions, its potential shifted after discovering its ability to inhibit acetylcholine esterase. While it was considered for various psychiatric treatments, its development for AD began only in the 1990s because of extraction and synthesis challenges. By 2001, the FDA approved it for treating mild to moderate AD (365–367).

In a Meta-analysis study by Deqi Jiang, the effectiveness and safety of galantamine in treating AD are evaluated By analyzing randomized controlled trials (RCTs) of galantamine for AD published until April 30, 2014, it found that 8–28 weeks of galantamine treatment (16–40 mg daily) led to significant improvements in several cognitive and behavioral assessment scales. A significant downside was that more adverse events and treatment dropouts were reported in the galantamine group compared to the placebo group. Overall, while galantamine improved cognitive, behavioral, and global functions in AD patients, its use in clinical treatment should be approached with caution due to potential side effects (368).

##### 11.1.2.4 Tacrine (Cognex)

Tacrine (Cognex) was the first drug approved by the US Food and Drug Administration (FDA) to treat AD and it received approval in 1993 (369). Tacrine's function as a drug-enhancing cognition is multifaceted and not fully comprehended. It acts on various pathways, including cholinergic, gabaergic, nitrinergic, and glutamatergic. Tacrine's effect on NMDA receptors (NMDAR) is more related to AD treatment, and its impact on glutamatergic neurons is direct (as an NMDAR antagonist) and indirect patterns and inhibits Ca2+-activated potassium channels. This prevents membrane repolarization, extends NMDAR activation, and leads to long-term potentiation. Therefore, tacrine-derivatives modulate both cholinesterase and NMDARs (370).

Tacrine can help improve cognition and function in some people with Alzheimer's, but its effect is modest. Like other treatments for Alzheimer's, it does not cure the disease but can help manage some of its symptoms. In 1998, in a meta-analysis study which is published in JAMA In from 12 trials with 1984 Alzheimer's patients, the use of Tacrine (a cholinesterase inhibitor) for 12 weeks improved cognitive performance, and there was a minor positive difference in the behavioral non-cognitive aspects (371).

They concluded that using Tacrine may slow cognitive decline in Alzheimer's patients in the initial 3 months and heighten the chances of overall clinical improvement. Still, unfortunately, the use of Tacrine was limited due to its side effects, particularly liver toxicity (372). Because of the liver toxicity concerns and the subsequent approval of other cholinesterase inhibitors that had more favorable side effect profiles, the use of Tacrine diminished over time. In summary, while Tacrine played a pivotal role in the history of AD treatments, it is not widely used today due to its side effect profile.

The clinical effectiveness of combining an acetylcholinesterase inhibitor (AChEI) with Memantine for Alzheimer's patients remains unclear when compared to the use of either treatment alone. To address this, a systematic review and meta-analysis of randomized controlled trials (RCTs) was conducted, focusing on patients with moderate to severe Alzheimer's disease. From the nine RCTs (involving 2,604 patients) reviewed, results indicated that short-term combination therapy had a more significant positive effect on cognition than AChEI monotherapy. However, there is a lack of evidence regarding the long-term effects and comparison with memantine monotherapy (62).

### 11.2 NMDA receptor antagonists in Alzheimer's disease

#### 11.2.1 Mechanism of action of NMDA Receptor Antagonists

Synapse dysfunction caused by amyloid beta (Aβ) is an initial occurrence in the development of AD. Prior research indicates that abnormalities in the N-methyl-D-aspartate (NMDA) receptor may play a role in this pathology. Notably, Aβ peptides have been shown to affect NMDAR expression and function negatively (373). NMDA receptors play a significant role in AD because they mediate calcium influx and maintain calcium homeostasis in the brain. Calcium dysregulation is considered a common mechanism in AD, and hyperactivity of NMDA receptors has been reported in the disease (374).

NMDA receptor activation is linked to synaptic dysfunction in AD. While synaptic NMDARs are protective, overactivation of NMDARs outside the synapse can lead to cell death. NMDA receptor dysfunction, involving pre- and postsynaptic neurons and glial cells, directly affects AD (375). Memantine, the most popular NMDA receptor antagonist, blocks excessive NMDA receptor activity without disrupting regular activity, providing neuroprotection and alleviating symptoms in AD patients (376). Memantine performs this by blocking the NMDA glutamate receptors, helping to regulate the glutamatergic system and improve cognitive and memory issues. Memantine's effectiveness relies on its unique binding to the NMDAR, where its low affinity and quick dissociation from the receptor channel ensures the receptor's normal function, making the drug well-tolerated with a minimal side effect profile (377).

#### 11.2.2 NMDA receptor antagonists types

##### 11.2.2.1 Memantine

Memantine is the most well-known NMDA receptor antagonist approved for treating moderate to severe AD. The European Federation of Neurological Societies- European Neurological Society/European Academy of Neurology (EFNS-ENS/EAN) conducted a study aimed to determine whether a combination of cholinesterase inhibitors (ChEI) and Memantine should be recommended over ChEI alone for patients with moderate to severe AD to improve cognition, behavior, and daily activities. This determination, conducted based on a systematic review and meta-analysis of randomized controlled trials sourced from the Cochrane Dementia and Cognitive Improvement Group's register enrolled 1,549 AD patients, indicated that the combination therapy had significant benefits compared to ChEI monotherapy. Also, it was mentioned that the strength of evidence varied for each parameter, with high quality for behavior, moderate for cognitive function, and low for activities of daily living (378). So, although the combination of ChEI and Memantine is suggested over the use of ChEI alone for patients with moderate to severe AD, this recommendation is given with a weak endorsement (378).

The use of Memantine as an NMDA receptor antagonist in combination with one of the three common cholinesterase inhibitors—donepezil, rivastigmine, and galantamine—has been studied for the treatment of AD for several years (379). A meta-analysis of 54 trials on the combination therapy of Memantine and Donepezil revealed that the combination of Memantine and donepezil demonstrated improved outcomes in cognitive function, daily function, and neuropsychiatric manifestations compared to monotherapy and placebo. But it has less acceptability among the patients. Combination therapy could be more economically viable, as Memantine slows the progression of AD.

In a study of Japanese patients in 2018, In a meta-analysis of multicenter, randomized, double-blind studies involving 633 Japanese patients with moderate to severe AD, the efficacy of Memantine (20 mg/day) proved. Statistically, a significantly reduced likelihood of clinical worsening across multiple rating scales compared to a placebo was concluded. There was a notably diminished risk of severe deterioration in the memantine group. It was also concluded that Memantine offers a promising treatment solution for AD patients, addressing not just cognitive challenges but also encompassing a wide array of symptoms, particularly the behavioral and psychological manifestations of dementia (380)

An updated systematic review and meta-analysis study evaluated Memantine's potential as an antidepressant for major mental disorders. Eleven double-blind RCTs containing 899 patients showed that Memantine significantly decreased depressive symptoms, especially in patients with mood disorders. Memantine appears to be an effective, well-tolerated treatment for depressive symptoms, particularly in mood disorder patients (381).

As mentioned, Memantine is approved by FDA for moderate to severe AD and its does not have FDA approval for mild cognitive impairment. Observational data from nearly 10,000 participants across 44 trials evaluated the benefits of Memantine for different types of dementia. The majority of trials focused on AD. For those with moderate-to-severe AD, high-certainty evidence demonstrated that Memantine consistently offered clinical benefits in areas such as clinical global rating (CGR), cognitive function (CF), daily living activities (ADL), and behavior and mood (BM). However, in mild AD cases, evidence indicated that Memantine might not be more beneficial than a placebo in most categories (382).

For vascular dementia, there is a probable small clinical benefit in cognitive function and behavior, but not in other metrics. While limited data exists for different dementia types, Memantine does not substantially differ from placebos in causing adverse events. Yet, it's more likely than a placebo to cause dizziness and possibly headaches but shows no difference in fall risks (382). Memantine has a small clinical benefit to individuals with moderate-to-severe AD, regardless of their concurrent use of a ChEI. However, it doesn't provide any notable advantage to those with mild AD. Due to the varied clinical manifestations of AD, it's improbable that a single drug will produce significant effects. Current evidence doesn't support the early use of Memantine in mild AD, even though it's a prevalent approach and extended trials are required to determine Memantine's long-term efficacy (382).

In the study by Taro Kishi on randomized trial studies, including 11 memantine vs. placebo and 17 memantine and cholinesterase inhibitors combination (M+ChEIs) vs. ChEIs, consisting of 7,567 patients evaluated, it was revealed Memantine alone showed significant improvements in Cognitive function and behavioral disturbances compared to a placebo. In an analysis of patients with moderate-severe AD, Memantine improved better in reducing behavioral disturbances than placebo. Also, it was shown that Memantine was combined with ChEIs, with more reduction in behavioral disorders and improved Cognitive function compared to ChEIs alone. Also, they demonstrated no notable differences in discontinuation rates among the groups (383).

In the safety and effectiveness analysis of cholinesterase inhibitors and Memantine for AD, which was conducted on 41 randomized controlled trials, it was demonstrated that for mild to moderate AD, Galantamine 32 mg/day with mean difference of −0.51 (CI: −0.67 to −0.35), Galantamine 24 mg/day: with −0.50 (CI: −0.61 to −0.40) and Donepezil 10 mg/day with −0.40 (CI: −0.51 to −0.29) had the best outcomes in improvement of cognition compared to placebo. Among the patients with moderate to severe AD, a combination of memantine 20 mg and donepezil 10 mg is recommended, with a value of 0.76 (95% CI: 0.39–1.11). It was also demonstrated that none of these medications improved neuropsychiatric symptoms (384).

Despite the extensive use of Memantine, there are also some adverse findings about its effects on AD. A meta-analysis was conducted in 2018 and published in the JAMA network to demonstrate the association of concomitant use of cholinesterase inhibitors or Memantine with cognitive decline in AD. They used a Meta-database of the Alzheimer Disease Cooperative Study and Alzheimer Disease Neuroimaging Initiative and 10 clinical trial studies with a pooled population of 2,714 enrolled. It was demonstrated that numerous participants who are administered ChEIs or Memantine often exhibit increased cognitive deterioration. This variance is almost equivalent to the anticipated effect sizes of the treatments examined in these studies. When designing clinical trials for potential AD treatments, the simultaneous use of ChEIs or Memantine, which may interfere with ADAS-cog results, should be considered. Any subsequent analyses that categorize based on ChEIs or memantine usage should be cautiously approached due to possible confounding factors (385).

This medication received FDA approval for the treatment of moderate to severe Alzheimer's dementia (386); however, it also has non-FDA and label uses for Mild to moderate Alzheimer's dementia and mild cognitive impairment (387). It is also used for neuropathic pain (388) and has subscriptions for neuropsychiatric disorders other than dementia-like bipolar disorder, major depressive disorder, obsessive compulsive disorder (OCD) (389, 390) and autism spectrum disorder (391). Recent studies revealed the potential therapeutic benefits of N-methyl-D-aspartate (NMDA) receptor antagonists, including the antidementia drug memantine. In a self-controlled cohort study using US administrative claims databases of the 312,336 patients exposed to Memantine, 60 outcomes were identified other than dementia and memory loss, which were categorized into five groups, including mental disorders, substance use disorders, gastrointestinal and colon disorders, pain and demyelinating disease. These findings indicate that NMDA receptor antagonists might possess a wider range of therapeutic applications than previously understood (392).

In the study by Shinji Matsunaga, the effect of Memantine on Lewy body dementia is investigated. It was revealed that Memantine wasn't beneficial in treating cognitive and motor symptoms of Lewy body disorders. However, when assessing the overall impression of the disorders, Memantine might be more effective than a placebo. Additionally, Memantine is well-accepted by patients (393).

##### 11.2.2.2 Ketamine (Ketalar)

Ketamin is a water- and lipid-soluble agent for anesthesia, and its effects come from interactions with various receptors, including the N-methyl-d-aspartate (NMDA). The liver metabolizes it, which can induce bronchodilation and stimulate the cardiovascular system. Clinically, ketamine is used for various stages of anesthesia, and it's termed “dissociative anesthesia” (394).

The antidepressant properties of ketamine were discovered unintentionally. Additionally, ketamine has fewer side effects in animals and humans. Studies have also shown that ketamine could be beneficial in treating other neurological disorders, such as Parkinson's disease, multiple sclerosis, and stroke. This article discusses the potential of ketamine as a preventive or treatment option for various neurological conditions (395).

Depression is associated with neurodegenerative diseases like AD. Traditional antidepressants, particularly selective serotonin reuptake inhibitors (SSRIs), show varying results in treating depression linked with AD. A recent analysis of 25 studies on fourteen antidepressant drugs found limited efficacy in alleviating AD symptoms. However, ketamine, a broad-spectrum NMDA receptor antagonist, has numerous benefits, including treating depression and its severe manifestations. Esketamine, a nasal spray variant of ketamine, was approved by the FDA in 2019 for persistent depression. Current research indicates ketamine might protect brain functions and reduce AD-associated symptoms (395).

##### 11.2.2.3 Dextromethorphan

The FDA approved Dextromethorphan as a cough suppressant in 1958. For the last half-century, it has been a prevalent ingredient in many over-the-counter cough remedies (396), but recently, new therapeutic indications have been revealed for Dextromethorphan.

Neurodegenerative disorders, such as Alzheimer's, are associated with chronic inflammation in the central nervous system, especially in Microglia and astrocytes. Diminishing this inflammation could reduce neuronal loss, a hypothesis backed by findings that regular use of non-steroidal anti-inflammatory drugs (NSAIDs) correlates with reduced neurodegenerative disease rates. However, NSAIDs might not be strong enough to combat established neuroinflammation. Dextromethorphan and other compounds have shown promise in lowering such inflammation, as they inhibit neurotoxicity caused by human microglia and astrocytes (397, 398). The medication also received approval for pseudobulbar affect (PBA), a condition characterized by unsuitable or heightened emotional reactions that frequently occur in adults with neurological disorders like amyotrophic lateral sclerosis (ALS), multiple sclerosis (MS), stroke, traumatic brain injuries and AD, or Parkinson's disease (399–401). A combination of dextromethorphan hydrobromide and the antidepressant bupropion hydrochloride is used to treat major depressive disorder (MDD), agitation in AD, and smoking cessation. In August 2022, the combination was approved in the USA for adult MDD treatment (402).

### 11.3 Antidepressants in Alzheimer's disease

#### 11.3.1 Mechanism of antidepressant (SSRI)

Although antidepressants are primarily used to treat mood disorders, recent studies suggest they could also help manage neuropsychiatric symptoms in AD patients, such as depression and agitation (403). However, research results on the connection between depression and AD have been mixed, and few studies have been conducted to guide physicians in selecting antidepressant medication for depression in patients with dementia (404–406). Although evidence for the efficacy of antidepressants in dementia is limited, selective serotonin reuptake inhibitors (SSRIs) are the most studied and well-tolerated antidepressants, which are preferred over other antidepressant medications (404–406). SSRIs increase serotonergic activity in the brain by decreasing the activity of the presynaptic serotonin reuptake pump by nearly 90% (407).

APOE ε4 carrier status has been associated with depression symptoms and higher cortisol levels, which can lead to a decrease in hippocampal volume (408). Depression may contribute to AD through the HPA axis, and current investigations are exploring its possible role as a risk factor or prodromal symptom of AD. Antidepressants diminish or counteract the risk of eventual AD outcomes (408). Antidepressants have been found to provide neuroprotective benefits for people with depression, improving cognitive function and long-term memory by increasing 5-HT levels (409). Additionally, antidepressants can delay the onset of AD by affecting neurotransmitter balance and inflammatory pathways (409). They can also improve mood and reduce depression and anxiety symptoms commonly experienced by AD patients, enhancing overall quality of life (410).

Selective Serotonin and Norepinephrine Reuptake Inhibitors (SNRIs), such as duloxetine (Cymbalta) and venlafaxine (Effexor), are antidepressants that influence serotonin and norepinephrine levels in the brain (411). These neurotransmitters play a crucial role in regulating mood and can help alleviate depressive symptoms in AD patients.

The diagnosis of depression in patients with dementia and impaired cognition presents complexities due to the potential for dementia to exhibit signs and symptoms resembling depression, even in the absence of the condition, as well as the ability of depression itself to mimic dementia in the absence of the latter (405, 412). In challenging circumstances, a treatment trial with antidepressant medication may be the only feasible diagnostic technique (405). Despite the limited availability of reliable diagnostic tools to diagnose depression in individuals with dementia (405, 413), the American Association for Geriatric Psychiatry has established diagnostic criteria for this condition (414).

#### 11.3.2 Dosage and administration of antidepressant

Although the choice of an SSRI is typically based on the side effect profile, medication interactions, and cost, the most commonly prescribed medications are citalopram and sertraline (405). Citalopram, with a starting daily dose of 5–10 mg and a maximum daily dose of 20 mg every morning or every evening, is recommended to reduce the risk of QT-prolongation in patients aged over 65 years old (405, 406). Sertraline with a starting daily dose of 12.5–25 mg and a maximum daily dose of 25–200 mg daily is suggested (405, 406). Fluoxetine is less desirable in older adults due to its long half-life and drug interactions, and Paroxetine's effects on cognition due to its anticholinergic impacts (405). Other types of antidepressant medication, such as serotonin-norepinephrine reuptake inhibitors (venlafaxine) and noradrenergic antagonists (mirtazapine), have not been well-studied, and tricyclic antidepressants (TCA) are not recommended due to cardiovascular and nervous system side effects (405, 406).

It should be noted that a physician should closely monitor the use of antidepressant medications in AD because these medications may require dose adjustments, have negative side effects, and interact with other medications the patient may be taking.

#### 11.3.3 Psychotherapy

In addition, clinicians should consider psychotherapy as an effective treatment for patients with mild to moderate dementia, including Multimodal CBT, Psychodynamic, interpersonal therapy, and Multifaceted and semi-tailored intervention (405, 415), which is underutilized in practice.

### 11.4 Anxiolytics in Alzheimer's disease

AD is a condition that causes a gradual loss of cognitive abilities. Research has explored the potential neuroprotective effects of N-methyl-D-aspartate (NMDA) receptor antagonists for AD. These antagonists are critical for synaptic plasticity and transmission (416). Excessive activation of glutamate receptors, particularly the NMDA type, can lead to excitotoxic effects on neurons and is thought to contribute to neurodegeneration. Synaptic dysfunction in AD may be attributed to atypical activation of the NMDA receptor. The NMDA receptor antagonist memantine is especially effective in blocking the receptor and reducing the influx of calcium (Ca2+) ions into neurons, thereby preventing toxic intracellular events from occurring (416). Memantine may slow cognitive decline by reducing excitotoxicity and promoting neuroplasticity through modulation of glutamate transmission (417). In AD, acetylcholine is a neurotransmitter that is significantly depleted. Some antidepressants, like trazodone, have anticholinergic properties, which can be useful for managing agitation and behavioral symptoms in AD patients by modulating the cholinergic system (418).

#### 11.4.1 Types of anxiolytics

##### 11.4.1.1 Benzodiazepines

Lorazepam (Ativan) and alprazolam (Xanax) are types of anxiolytic medications known as benzodiazepines, which act on GABA-A receptors in the brain (419). Although benzodiazepines are frequently prescribed for anxiety and sleep conditions, their usage in AD is a matter of debate owing to the potential risks involved (420, 421). Clinicians typically avoid administering benzodiazepines to older people with dementia as these drugs cause confusion and slow down mental processes. It is recommended to be cautious when using benzodiazepines in AD and consider alternative treatment options. While they are not typically the first choice for managing anxiety in AD due to their potential to cause sedation, confusion, and an increased risk of falls, they may be considered in some cases when other options are ineffective (422).

##### 11.4.1.2 Buspirone (Buspar)

Buspirone is a partial agonist at serotonin receptors (5-HT1A) and is classified as a non-benzodiazepine anxiolytic (423). Earlier evidence indicates that buspirone may be an effective non-sedating option for managing anxiety symptoms in patients with AD (424). Buspirone is a useful treatment for managing behavioral issues in individuals with dementia (425).

##### 11.4.1.3 Hydroxyzine (Vistaril)

Hydroxyzine is an antihistamine that has sedative properties and can be used to manage anxiety in AD patients. It has been found to be just as effective as other anxiolytic agents like benzodiazepines and buspirone, and is well-tolerated (426). However, it may cause sleepiness or drowsiness more frequently than the other medications (426). It's important to be careful when taking hydroxyzine because it can cause drowsiness and anticholinergic side effects, which could worsen cognitive decline (427).

##### 11.4.1.4 Gabapentin (Neurontin)

Gabapentin is not a first-line treatment for anxiety in AD, but it may be considered in cases where other options have failed (428). The mechanism of action of gabapentin to alleviate anxiety is not clear, but it is believed to regulate the release of neurotransmitters. By binding to the α2-δ subunit of voltage-gated calcium channels, gabapentin may hinder neuronal signaling in the central nervous system, leading to a reduction in the release of excitatory neurotransmitters and a decrease in anxiety (429). Additionally, gabapentin may reduce the formation of excitatory neuronal synapses, ultimately resulting in a decrease in overall neuronal activity and anxiety (430). Gabapentin may also raise the levels of gamma-aminobutyric acid (GABA), an inhibitory neurotransmitter that can help reduce anxiety (429).

### 11.5 Monoclonal antibodies

#### 11.5.1 Aducanumab

Aducanumab, a monoclonal antibody that targets amyloid β (Aβ) aggregates, was the first drug approved by the FDA in June 2021 for address the underlying biology of Alzheimer's disease. It is approved for treating patients with mild cognitive impairment (MCI-AD) or those in the mild dementia stage. This approval, granted through the accelerated pathway, was based on aducanumab's ability to clear Aβ plaques, despite no direct evidence linking plaque removal to reduced cognitive or functional decline. Aducanumab is a IgG1 antibody with high affinity for a specific conformational epitope on both soluble oligomers and insoluble fibrils of β-amyloid, a key protein involved in Alzheimer's disease (431–434).

The decision to approve aducanumab sparked significant controversy, as results from the Phase 3 trials (EMERGE and ENGAGE) were conflicting, and *post-hoc* analyses failed to definitively prove efficacy. Concerns were also raised about the drug's high cost, safety profile, and potential to slow further progress in Alzheimer's research. The European Medicines Agency's rejection of aducanumab in December 2021 added to the controversy. Biogen is currently conducting a confirmatory study, ENVISION, which is expected to be completed by 2026 (432–434).

#### 11.5.2 Lecanemab

Lecanemab, an FDA-approved antibody targeting amyloid beta (Aβ), is used to treat early Alzheimer's disease by binding to soluble Aβ protofibrils, which are more toxic to neurons than monomers or insoluble fibrils. The Clarity AD study, an 18-month multicenter, double-blind, placebo-controlled trial with an open-label extension, evaluated lecanemab's safety and efficacy (435, 436). Lecanemab received approval based on the Clarity phase 3 trial, which showed reductions in amyloid plaque burden and cognitive decline. However, three significant concerns warrant caution before the widespread adoption of the medication: the limited magnitude of its beneficial effects, the considerable risks associated with its use, and its potentially unprecedented costs. Although lecanemab demonstrated a statistically significant impact on cognition, the effect size was minimal and may lack clinical significance, falling below independent estimates of the minimally important clinical difference. This suggests that the cognitive improvements may be imperceptible to most patients and caregivers (437).

#### 11.5.3 Donanemab

Donanemab is a humanized antibody targeting the reduction of amyloid β (Aβ) plaques in Alzheimer's disease (AD) patients. A randomized clinical trial assessed the efficacy and adverse events associated with donanemab, a monoclonal antibody designed to clear brain amyloid plaques, in early symptomatic Alzheimer's disease. Conducted across 277 medical research centers in eight countries, this multicenter, double-blind, placebo-controlled, 18-month phase 3 trial enrolled 1,736 participants diagnosed with mild cognitive impairment or mild dementia who exhibited amyloid and low/medium or high tau pathology based on positron emission tomography imaging. The results indicated a significant difference in the integrated Alzheimer Disease Rating Scale score at 76 weeks, with the donanemab group showing a mean change of−6.02 compared to−9.27 in the placebo group for the low/medium tau population, and −10.19 vs. −13.11 in the combined study population. These findings demonstrate that donanemab treatment significantly slowed clinical progression in these patients (438).

A systematic review analyzes four studies involving 396 adult patients treated with Donanemab compared to a placebo or standard care (228 vs. 168 participants). The results indicated that Aβ-plaque reduction was influenced by baseline levels, with complete amyloid clearance observed in those with lower baseline levels (<24.1 Centiloids). The treatment also slowed tau accumulation and led to a 32% reduction in cognitive and functional decline. However, safety concerns were raised due to Amyloid-Related Imaging Abnormalities (ARIA), which occurred in 26.1–30.5% of participants across trials. While preliminary evidence suggesting that Donanemab may delay cognitive and functional decline in patients with mild-to-moderate AD, it remains uncertain whether it provides significant therapeutic benefits that alter the clinical status of these patients (439). Further research is needed to investigate the relationship between Aβ-plaque reduction and tau toxicity in achieving meaningful clinical outcomes for AD patients experiencing cognitive impairment.

Finally, evidence suggests that the effectiveness of both Donanemab and Lecanemab diminishes as disease severity increases. In Phase 3 trials, patients with lower amyloid burdens at baseline experienced greater overall treatment benefits, while those with higher amyloid burdens showed less improvement. Ongoing studies, such as the AHEAD study (NCT04468659) and TRAILBLAZER-3 (NCT05026866), are now investigating these treatments in pre-symptomatic Alzheimer's populations, further supporting the rationale that earlier intervention may yield more effective outcomes.

## 12 Non-pharmacological management strategies

Beyond pharmaceutical interventions, a variety of non-pharmacological strategies, including cognitive stimulation therapy, reality orientation therapy, music therapy, physical exercise, and occupational therapy, have demonstrated comparable efficacy and may be used as complementary to them.

Cognitive stimulation encompasses activities and discussions such as reminiscence therapy and reality orientation therapy, which are as effective as cholinesterase inhibitors in mitigating cognitive decline and have become standard practice for patients with mild-to-moderate dementia (440, 441). The primary purpose of cognitive stimulation therapy is to retain and develop the cognitive capacities of those with dementia, therefore reducing the disease's progression. It promotes and maintains mental capacities, social interactions, personal autonomy and self-esteem, control of stress and abnormal psychological reactions, cognitive and functional performance, daily living activities, and the overall quality of life for patients and caregivers (442). Studies on the efficacy of cognition stimulation therapy for AD have yielded contradictory results. Still, a systematic review of these studies found that this intervention model preserved the cognitive performance of patients with mild-moderate AD regarding reasoning, constructive praxis, and word list recognition. In contrast, the control group deteriorated in these functions over 6 months (442).

Reality orientation therapy is an intervention for individuals with amnesic impairments, confusion episodes, and spatial-temporal disorientation, which are common among patients with dementia, especially AD patients. The primary goal of reality orientation therapy is to reorient patients with their history, surroundings, and time through repeated multimodal stimulations. It employs both formal and informal modes of operation, with formal reality orientation therapy carried out in small groups of cognitively impaired individuals, giving scheduled interactions for around 45 min every day, with promising outcomes in the treatment of dementia (443). Reality orientation therapy has been demonstrated to supplement pharmaceutical therapies for AD. For example, an RCT on patients with Alzheimer's showed that 6-month reality orientation therapy combined with acetylcholinesterase inhibitors could significantly improve cognitive outcomes compared to administering acetylcholinesterase inhibitors alone (444).

Music therapy is another non-pharmacologic treatment option for AD that can be used to distract patients, assist them in coping with emotional and affective issues, and encourage them to live longer while mitigating behavioral and psychological symptoms of dementia by making it easier for them to recall episodic memories, even if the music is unrelated to the memories they are recalling (445, 446). MEAMs (music-evoked autobiographical memories) can be sparked by a musical cue. They can reveal knowledge about one's past, which may be exceptionally vivid, incredibly thorough, and spontaneously remembered, as well as evoke robust emotional responses (447). Music-evoked emotions have been discovered to colocalize in the anterior hippocampus with MEAMs, underlying the facilitation of memory recall by music (448). In this way, several RCTs were launched to evaluate the efficacy of music therapy for AD. According to a trial, music, particularly active musical therapy through singing, could effectively enhance both immediate and delayed memory and language abilities in mild patients and psychiatric symptoms in moderate or severe patients; however, the effects did not last longer than 3 months (449). Although mechanisms behind the therapeutic benefits of music therapy in AD have remained elusive, several studies have established that these mechanisms involve stimulation of neurogenesis and neuroplasticity, enhancement of dopamine release, and immune system modulation (446).

Physical exercise has also been shown in multiple RCT and non-RCT trials to improve cognition function, activities of daily living, neuropsychiatric symptoms, and physical function in AD patients. In addition, when compared to pharmaceutical therapies such as cholinesterase inhibitors, physical exercise has demonstrated higher efficacy and adherence (450).

Adding occupational therapy to routine clinical care can significantly enhance patient care by providing caregivers with guidance on compensatory and environmental strategies for managing patients' cognitive decline (451). This approach reduces caregiving stress and empowers patients by improving their coping techniques, ultimately minimizing their dependency. Specifically for individuals with dementia, this therapeutic process involves assisting them in identifying personally meaningful activities, streamlining their implementation to maximize engagement, and creating an environment free from stressors and distractions (452). Several home-based occupational interventions have been developed, including a tailored activity program (TAP), the environmental skill-building program (ESP), and advancing caregiver training (ACT). Briefly, ESP offers patients tailored activities to their capabilities while educating caregivers to generalize these activities to new settings, which are advantageous in lowering caregiver strain and being cost-effective (453, 454). On the other hand, ESP incorporates strategies to modify the home environment's physical, social, and task aspects (455). Additionally, the ACT approach involves creating an action plan for patients that includes particular behaviors, goal-setting triggers, and management strategies. At the same time, caregivers receive training in stress management, self-care strategies, and hands-on training for daily living activities (456). Dance and yoga, gardening, mealtime activities, montessori programs, and animal-assisted therapy are among occupation-based therapies that have shown positive effects. These therapies target different sensory systems while providing meaningful and social engagement (457). Occupational therapists can use these interventions to boost patients' engagement and benefits, but further research on their efficacy and delivery by skilled professionals will be required.

## 13 Lifestyle changes to improve Alzheimer's disease management

Lifestyle medicine exploits evidence-based lifestyle interventions in which healthcare practitioners collaborate with patients to evaluate and change various aspects of their lifestyle to avoid, manage, and reverse chronic disease (458). Several lifestyle changes have been proven advantageous in AD management, including a healthy diet, regular exercise, good sleep hygiene, socialization and engagement with hobbies, and stress management.

Multiple healthy diet patterns have been identified to affect the disease course in patients with AD. Some studies have shown the beneficial effects of the Mediterranean diet, unsaturated fats, vitamins, polyphenols, trehalose, and cocoa and cocoa-derived products on AD prevention and management (458, 459). Although many nutrients have been shown to be implicated in neuroprotection at the cellular and molecular levels and even to significantly lower the risk of AD development in populations, it can be challenging to recommend a precise diet for patients with AD as the metabolism of many nutrients is disrupted by the disease (459). For example, epidemiological and clinical studies have shown that adequate intake of omega-3 fatty acids can be advantageous for the prevention of AD, but when the neuropathological state progresses, it is unable to maintain or ameliorate the condition (460, 461). Multiple clinical studies, on the other hand, have shown that a ketogenic diet for patients with AD can significantly improve activities of daily living, quality of life, and cognitive measures (462–464).

Multiple studies have also found that regular physical activity has a considerably favorable effect on symptom relief in patients with AD by inhibiting multiple neuroinflammatory pathways implicated in disease pathogenesis (465, 466). Similarly, a recent systematic review and meta-analysis of 18 RCTs on patients with AD or associated dementia of varied forms and severity found that physical activity might considerably enhance and prevent the decline in executive function (467). Another systematic review and meta-analysis of 15 RCTs on AD patients demonstrated that aerobic exercise of 30 min per session, <150 min per week, and up to three times per week can significantly improve cognitive function, as measured by the MMSE score, with the most significant impact in patients with poorer basal cognitive status (468).

Circadian dysfunction is closely linked to AD pathology, with sleep disturbances playing a significant role in impaired neuroplasticity and enhanced neurodegeneration. Aβ and tau pathology interact with circadian rhythms, contributing to sleep system dysfunction (469, 470). Thus, good sleep hygiene, which may be improved by encouraging regular physical activity and adhering to a regular wake-sleep cycle, limiting naps during the day, and not being exposed to light and noise at night, is an essential step forward in the management of AD (470).

Given the well-established benefits of socializing in neuroplasticity, investigations on patients with AD have yielded promising results. This approach revealed that mild-to-moderate AD patients who participated in socialization procedures for more than two semesters exhibited no year-to-year loss in several indicators of cognitive performance (471). Moreover, socialization strategies can potentially improve quality of life, cognitive stimulation, and emotional wellbeing. Social interaction and participation in hobbies aid in the maintenance of language abilities, the reduction of behavioral problems, and the postponement of cognitive decline (472). Improved quality of life is aided by family support, peer engagement, and social activities. Furthermore, spirituality and religion function as coping techniques and can improve general wellbeing in patients with AD (472).

The motor and cognitive malfunctions seen in AD patients not only cause stress but also have the potential to disrupt the brain circuits responsible for managing stress responses. Chronic stress in AD patients stimulates the hypothalamic-pituitary-adrenal axis and increases corticotropin release, resulting in oxidative stress and toxicity of β-amyloid peptide, which has neurotoxic effects on the hippocampus. These effects ultimately impact cognition and contribute to the progression of AD, highlighting the significance of stress management in these patients (473). Stress management entails coping methods that incorporate both physical and emotional changes. Coping comes in two different forms: problem-focused coping and emotion-focused coping. Individuals employing emotion-focused coping strive to alleviate the negative emotions triggered by stress without necessarily altering the challenging environment (474). Engaging in preferred hobbies, exercising, resting, seeking social support, self-indulgent escapism, seeking distance, negative avoidance, and adapting to stress can all fall under this category (475). On the other hand, problem-focused coping involves actively handling stressful situations through problem-solving, daily activity planning, convincing oneself of the patient's impairment, and distracting oneself (475, 476).

## 14 Future directions in Alzheimer's disease treatment and management

Regardless of the numerous studies, at this moment, there is no definite treatment option for AD (477, 478). Non-pharmacological therapies like exercise therapy, music therapy, light therapy, massage therapy, and cognitive training have been investigated and none of them makes a great difference in AD signs and symptoms (479–485).

In spite of all that, scientists are still looking for a new treatment strategy hoping to find an efficient one. Current research aims the therapeutic approaches to slow or stop the progression of AD. They consider every aspect of the disease, like biology and diagnostic markers, to help them design clinical trials and find the effective treatment (486, 487).

### 14.1 Immunotherapy

Active and passive immunization are the two immunotherapeutic approaches which promote amyloid beta and tau clearance.

Some anti-Aβ antibodies have shown promising effects in trails, including aducanumab (488), lecanemab (435), and donanemab (438). The first two were approved by the US FDA in 2021 and 2023, respectively. Solanezumab is a monoclonal antibody (mAb) that targets monomeric Aβ peptide. It has been investigated in phase 3 clinical trials, with three unsuccessful trials reported in 2014, 2021, and 2023 (489–491). Bapineuzumab (492), crenezumab (493), and gantenerumab (494) are also anti-Aβ antibodies which have failed to show beneficial effects in improving cognitive functions in phase 3 clinical trials in AD patients. To date, three anti-tau antibodies—semorinemab (495), gosuranemab (496), and tilavonemab (497)—have failed to slow the progression of AD in phase 2 trials.

New emerging therapies are being investigated to identify new therapeutic agents for AD. This includes BIIB080, an investigational therapy and an antisense oligonucleotide (ASO) targeting tau expression. In a phase 1 randomized clinical trial, BIIB080 was evaluated in AD patients and was found to reduce tau biomarkers. The effects of this agent are being further evaluated in phase 2 trials (498). Another new agent under study is trontinemab, a bispecific modular fusion protein composed of gantenerumab and a human transferrin receptor, which aids in crossing the blood-brain barrier. This agent is still being studied for potential use in clinical trials (499).

Intravenous immunoglobulin (IVIg) and plasma exchange are two other treatment options which have been studied but not shown as promising results as mAbs (500, 501).

### 14.2 Gene therapy

Gene therapy uses different strategies and targets specific regions of the brain in order to treat AD, for example, acting directly on amyloid precursor protein, increase neuroprotection, boosting autophagy-mediated pathways, targeting inflammatory pathways, and modulating genes related to lipid metabolism (502). Most of these strategies exhibit positive results in improving cognition in animal models. However clinical studies have shown inconclusive results mostly related to delivery methods used in gene therapy (503). Gene therapy has shown a great potential for treating AD, but for now clinical results are not ready to be relied on and it needs more evaluations in order to be used in AD treatment.

### 14.3 Stem cell therapy

The majority of stem cells used in AD therapy include neural stem cell (NSC), mesenchymal stem cell (MSC), embryonic stem cell (ESC), and induced pluripotent stem cell (iPSC) (504). NSC has been studied on cellular level and animal models (505–512). Generally, NSC therapy appears to be more effective in early stage of AD. MSC therapy is studied the most and has entered clinical levels (513). It uses different mechanisms in the brain and ultimately can improve cognition (514). ESC is studied in animal models and higher memory ability was achieved as the result (515). In the past few years iPSC is the most studied stem cell in clinical levels and has shown some promising results, but there is a risk for infection and tumorigenicity which indicates the need for more investigations (516, 517). Overall, stem cell therapy has shown to be a promising treatment for AD in the future, but for now it needs a lot more investigations specially in clinical levels to become available.

### 14.4 Digital therapeutics

Digital therapeutics (DTx) is an emerging field that provides evidence-based therapeutic interventions via internet and software, employing tools such as mobile devices, computers, videogames, apps, sensors, and virtual reality. DTx is used as an adjunctive therapy in AD for improving memory, cognition, functional abilities, and managing motor symptoms (518).

## 15 Challenges and considerations in Alzheimer's disease treatment and management

Longitudinal population studies have provided evidence of a decline in the incidence and prevalence of Alzheimer's disease and related dementias (ADRD) in high-income countries when adjusted for age. However, the overall number of patients with ADRD continues to rise due to the aging population (519, 520).This reduction has been attributed to improved management of modifiable risk factors like cardiovascular health and higher levels of education. These findings highlight the role of modifiable risk factors in the development of AD and emphasize the need for ongoing efforts in primary prevention within the field of dementia management (521).

Several studies consistently demonstrate an increased risk of dementia and AD associated with vascular and metabolic risk factors such as hypertension, hypercholesterolemia, midlife obesity, diabetes mellitus, and atherosclerosis. Hypertension, in particular, is a treatable risk factor for ADRD (522). However, the relationship between blood pressure and ADRD is complex. Midlife hypertension (defined as BP ≥ 140/90 mm Hg between 40 and 65 years of age) has been consistently linked to an elevated risk of ADRD in longitudinal studies. The Honolulu-Asia Aging Study, which examined a 3,703 Japanese American men cohort, was the first to report this association. High systolic blood pressure (≥160 mm Hg) was associated with an increased risk of dementia in the untreated group (OR, 4.8; 95% CI, 2.0–11.8). Subsequent population studies have confirmed this finding (523). The relationship between hypercholesterolemia and AD risk is less clear and likely multifaceted (524). Similar to other vascular risk factors, this association may be partially explained by survival bias and competing mortality associated with elevated cholesterol due to premature cardiovascular death. However, there have also been reports indicating that unintentional decreases in cholesterol levels in late life may signify an elevated risk of AD and related dementias, rather than serving as a protective factor (525).

Diabetes is considered a lifelong risk factor for ADRD, including both juvenile type 1 and adult-onset type 2 diabetes, and is associated with a 1.5- to 2.5-fold increased risk of ADRD [41]. AD and diabetes mellitus share common features such as increased prevalence after the age of 65, significant impact on quality of life, and increased healthcare costs. The Rotterdam Study, which followed a population-based cohort of 6,370 elderly individuals (≥55 years of age), found that the presence of diabetes nearly doubled the risk of AD (526).

Midlife obesity, defined as a body mass index (BMI) > 30 kg/m^2^ between the ages of 35 and 64 years, affects over 10% of adults in the Western world. The relationship between midlife obesity and AD risk is well-documented, with studies consistently reporting an increased risk of ADRD associated with obesity during this period (527). Numerous epidemiologic studies have demonstrated a link between midlife body mass index (BMI) and the risk of developing Alzheimer's disease and related dementias (ADRD) later in life, independent of other vascular or socioeconomic risk factors. These findings highlight the importance of maintaining a healthy BMI throughout adulthood. Additionally, epidemiological studies have confirmed that smoking is a risk factor for ADRD (528). The Rotterdam Study, a population-based follow-up study involving 6,870 individuals aged 55 years and older, investigated the impact of smoking on ADRD risk. After an average follow-up of 2.1 years, the study concluded that smokers had a higher risk of developing AD (relative risk, 2.3; 95% CI, 1.3–4.1) (529).

Diagnosing AD promptly allows for earlier intervention, but the stigma associated with the diagnosis, coupled with the lack of effective therapeutic options, can discourage individuals from seeking help. Social stigma surrounding an AD diagnosis is prevalent in certain societies and cultures, further discouraging patients from reporting early symptoms to their general practitioners and delaying early intervention or support. Psychological complications, particularly in the years following a dementia diagnosis, may contribute to an increased risk of suicide (530). Another significant issue is the lack of awareness among patients, their families, and general practitioners regarding dementia and its symptoms. Patients often dismiss dementia symptoms as a normal part of aging and therefore do not disclose them to their doctors. Some general practitioners also struggle to differentiate between cognitive impairment and normal aging. This normalization of symptoms leads to delays in seeking help. Patients and general practitioners often prioritize physical health over mental health, which may result in the dismissal of dementia symptoms. In addition to directly impacting the patient, a diagnosis of dementia has a negative impact on families and caregivers (531).

When AD is suspected, it is crucial to conduct thorough testing to rule out other conditions that may have similar signs and symptoms, especially since some of these conditions may be reversible. More extensive testing is necessary to minimize false positive and false negative results. In the middle and later stages of AD, ethical dilemmas arise regarding balancing patient autonomy with the need to protect them. Caregivers may face challenges regarding whether they should influence the patient without disclosing their intentions, withhold information to prevent distress, or even resort to lying to avoid causing psychological stress, particularly during the advanced stages of the disease. As AD patients approach the end of life, questions arise regarding the extent to which extraordinary measures should be taken to prolong their lives. As the patient's condition deteriorates and their existence becomes highly burdensome, some argue that it may be more humane to allow natural death, even in the absence of available beneficial treatments. Psychiatrists should provide patients who desire access to information about their condition, even when effective treatments are unavailable, as long as the information has meaningful prognostic implications (532). A meta-analysis by Martyr et al. identified several factors associated with better quality of life (QoL) in AD patients. These factors include a positive relationship with the caregiver, religious beliefs or spirituality, and active social engagement. Other important factors included functional ability, self-rated health and awareness, receiving care in a specialized dementia unit or living with a spouse, and being of white race. Demographic factors were found to have minimal impact on QoL. On the other hand, neuropsychiatric symptoms such as depression were strongly associated with poorer QoL. Additionally, poorer QoL was linked to more severe dementia, other medical conditions, pain and anxiety, unmet needs, and living alone (533). Studies examining different severity stages of dementia consistently show that patients with moderate-to-severe or severe dementia incur higher societal costs compared to those with mild or moderate dementia. The difference in costs between severity groups was statistically significant in all studies where significance was reported (533).

## 16 Caregiving for Alzheimer's patients

### 16.1 Caregiving for Alzheimer's patients

As previously mentioned, caregivers play a crucial role in detecting early changes in a patient's cognitive abilities and can significantly contribute to the early diagnosis of individuals with AD. Therefore, it is propose that mHealth applications designed to support caregivers should incorporate a category of AD diagnosis tests. This functionality should encompass various cognitive dimensions and track the progression of cognitive impairment over time. This feature allows caregivers to administer cognitive tests to identify early signs of cognitive decline and share the results with physicians to facilitate personalized treatment options (534).

Spouses and adult children who care for family members with AD often experience five distinct phases of caregiving throughout the course of the disease. These phases involve monitoring initial symptoms, navigating the diagnosis process, assisting with instrumental and basic activities of daily living, and preparing for the future. Although it is evident that involving AD caregivers in the patient's care management is beneficial, they are frequently excluded from the care team or undervalued in their role. Many caregivers desire active involvement in decision-making processes related to AD care, ranging from diagnosis to treatment (535, 536). Considering that AD is characterized by progressive cognitive decline, the information that caregivers can provide is notable. They can advocate for the patients and offer insights that no other healthcare professional within the care team can provide. Additionally, caregivers often neglect their own needs while providing care, leading to a higher likelihood of having multiple chronic conditions. For instance, studies have shown that 35% of caregivers aged 45–64 and 53% of those over 65 have two or more chronic conditions. Individuals with chronic conditions may face limitations due to their own illness, making it increasingly challenging to provide care as the disease progresses and requiring more extensive self-care (537, 538).

Incorporating caregivers as valuable assets within the healthcare team model can significantly enhance the quality of care provided. Previous reviews of dementia knowledge training for caregivers have demonstrated that such education generally leads to improved caregiver outcomes. However, most of these reviews have focused on training content, such as attitudes, beliefs, confidence, perceived competence, and self-efficacy. Additionally, these reviews have primarily assessed the effectiveness and outcomes of interventions rather than focusing on the evaluation instruments utilized in the programs. To address this, Burgio and colleagues recommended using standardized and recognized tools to prevent biased results and enhance the methodological rigor of studies. A previous systematic review of five validated instruments for evaluating dementia knowledge identified several limitations, including weak psychometric properties, outdatedness, and limited scope. Therefore, there is a clear need to understand further dementia knowledge instruments developed by the authors and previously published to improve their effectiveness and applicability. When individuals with dementia experience communication difficulties, it becomes increasingly challenging for them to participate socially and engage in interactions. This can lead to social isolation and hinder their ability to maintain relationships and contribute to society (539). Recognizing the importance of addressing both the cognitive and social consequences of dementia, the INTERDEM Social Health Taskforce and the European Working Group of People with Dementia (EWGPWD) emphasize the need to prioritize social health for this population (540).

One approach to improving communication between caregivers and individuals with dementia is through caregiver communication enhancement education and training techniques. One example of such a program is “FOCUSED” (541). FOCUSED is a structured program that aims to provide caregivers with information about AD and communication, correct any misconceptions about communicating with individuals diagnosed with the disease, and offer techniques to maximize communication potential (542). Various strategies are recommended, including using closed-ended or choice-based questions instead of open-ended ones, using direct and straightforward phrases, repeating key words and ideas, noting changes in conversation topics, employing direct contacts such as touch and eye contact, and utilizing comments and non-verbal cues to maintain the quality and flow of conversation (543, 544).

Other similar programs incorporate additional strategies, such as providing positive feedback when individuals with dementia follow through on requests, allowing sufficient time to respond to questions or requests, and engaging them in conversations about their lives and interests. Caregivers are also advised to provide specific instructions rather than general ones, use one-step instructions, and offer positive comments to the person with dementia (545). Furthermore, some programs teach caregivers personalized strategies to enhance communication with specific individuals with AD. These approaches rely on “conversation analysis”, which helps caregivers identify effective and ineffective conversation techniques and utilize the participants' strengths to improve their interactions. Additionally, activity-based interventions to increase communication can be conducted individually or in groups, incorporating specific activities (e.g., meal preparation) or various activities to stimulate communication. These groups typically focus on improving or maintaining functional skills, with communication being a central aspect (545, 546).

### 16.2 Self-care for caregivers

As discussed in the previous part, caregivers of Alzheimer's patients face many challenges. For maintaining physical and mental health, engaging in self-care strategies is crucial. It has been shown that self-care practices for professional caregivers have reduced occupational stress and increased resilience, a critical skill one acquires to bounce back from difficulties and stress at work (547). In addition to lowering burnout, distress, and diminished competence, self-care provides a positive and supportive work environment (548).

Also, there are many informal caregivers (ICGS) for Alzheimer's disease and related dementias (ADRD). In contrast to non-caregiving populations, ADRD ICGs practice less self-care (549), and stress and burden have been associated with less effective self-care (550, 551). Also, as a recent study showed, ICGs demand not only personal needs like maintaining their physical and mental health; providing enough information, awareness, and insight are also critical components that should be provided (552). In this article, we mention some important points of self-caring for both ICGs and formal caregivers in individualized institutions.

For caretakers, self-care must include both exercise and a balanced diet. Physical exercise could enhance mood, lessen stress, and increase general wellbeing (553). In addition, a balanced diet full of fresh produce, including vegetables and fruits, lean meats, and whole grains, may offer careers the nutrients they need to promote their wellbeing (554).

Caregivers may benefit from mindfulness and relaxation practices to reduce stress and improve their psychological wellbeing. According to studies, mindfulness techniques like deep breathing exercises and meditation aid in reducing symptoms of stress, depression, and anxiety. Regularly practicing mindfulness may assist caregivers in developing a feeling of peace and enhance their capacity to deal with the difficulties of caring (555, 556).

Caregivers should take time for themselves and partake in activities they like. Maintaining personal interests and hobbies is imperative. According to a study, satisfying activities may lower stress levels and enhance general wellbeing (557). Caregivers should recharge and keep a sense of identity apart from their caring position by taking breaks from their duties and making time for personal interests (558).

Social engagement and receiving social support are other examples of self-care demands. Studies of mostly female ADRD ICGs showed that support networks are crucial for stress management, sharing responsibilities and caring tasks, advocating for self-care, validating ICGs' needs for self-care, and offering self-care advocacy (559–563). Another study on ADRD ICGs showed that engaging in spiritual and religious activities like attending churches and praying provides them courage and emotional support (561, 564, 565).

In summary, self-care practices are essential to minimize adverse health consequences from caring for Alzheimer's patients and to achieve optimum health and quality of life outcomes. Caregivers must remember that not only prioritizing self-care and self-management is not selfish, but it is also a demand for patients' health.

#### 16.2.1 Legal considerations

Alzheimer's is an ongoing and disabling disease, so its financial cost is incredible. Due to that, the legal and financial issues involved in providing care for a person with AD should be understood by caregivers. Caregivers may aid patients' families with ensuring their loved ones get the care they need by planning and consulting experts when required (566–569).

A power of attorney document enables a person with dementia (referred to as the principal) to designate another person (referred to as an agent or an attorney-in-fact), typically a domestic partner, spouse, entrusted family member, or friend to handle financial and other decisions on their behalf when they are no longer able. The agent should be carefully selected, and it is advised that they have a lengthy discussion with the principal regarding the duties involved. If the principal is unable or unwilling to serve, a replacement agent or agents should be decided on. The agent is tasked with carrying out the principal's instructions and acting in the person's best interests. Unlike the principal, a power of attorney does not give the agent the power to make decisions until the person with dementia loses mental competence (570).

A guardian is chosen by the court to manage a person's assets and care. Guardianship is often considered when a dementia patient can no longer manage their own care, and either the family cannot agree on the required care, or there is no family. A guardian ensures that the person's daily requirements for safety, food, housing, and care are satisfied, in addition to making financial and healthcare choices. The court is ultimately accountable for and in charge of guardians (571).

#### 16.2.2 Insurances

Medicare and Medicaid, the two largest public insurance programs, provide healthcare services to their beneficiaries. Medicare offers services that include hospital insurance (Part A), outpatient medical insurance (Part B), prescription drug coverage (Part D), and additional benefits such as vision, hearing, or dental care (Part C). Medicaid, a joint federal and state program, provides free health insurance coverage to low-income children and their caregivers, pregnant individuals, the elderly, individuals who are blind, and those with other diabilities who incur high medical expenses (572, 573).

US Public Health Insurance revenue, including the Medicare Trust Fund, is sourced from payroll taxes, federally allocated budgets, monthly premiums paid by beneficiaries, and interest earning. These funds are subsequently divided and allocated to cover plans from Part A to Part D. Reimbursement fees paid to healthcare providers constitute a significant portion of total fund disbursement, necessitating careful assessment and allocation of resources. Medicare provides reimbursement to healthcare professionals and encourages them to treat patients under insurance coverage with high quality care. This is achieved by setting a “Fee Schedule” and negotiating with healthcare providers to prevent excess charges on patients and the healthcare system, while also supporting the adoption of innovative advancements (573, 574). In contrast, inappropriate reimbursement can lead to devastating consequences, including financial losses, time-consuming investigations conducted by the CMS (Centers for Medicare & Medicaid Services) to identify overbilling and fraud, inequities, and loss of participation in medicare (575).

Reimbursement criteria are updated frequently, and insurance companies adjust their policies based on market trends, changes in healthcare legislations, advancements in patient therapy guidelines, and the development of new technologies. This necessitates that beneficiaries regularly consult with their insurance providers to stay informed about their coverage (573, 576).

## 17 Conclusion

The present review provides an extensive overview of Alzheimer's disease, shedding light on the multifaceted aspects of this neurodegenerative disorder. The review underscores the growing prevalence of Alzheimer's, emphasizing the critical need for enhanced awareness and research to address the burgeoning public health challenge. It delineates the array of risk factors, both genetic and environmental, that contribute to the disease's onset and progression. Symptomatically, the article details the cognitive and behavioral manifestations of Alzheimer's, highlighting the importance of early and accurate diagnosis through advancements in neuroimaging and biomarkers. The management and caregiving sections stress the necessity of a holistic approach, combining pharmacological and non-pharmacological strategies to improve patient outcomes and support caregivers. The comprehensive nature of the article provides a valuable resource for clinicians, researchers, and policymakers, advocating for a concerted effort to advance understanding, treatment, and care for individuals affected by Alzheimer's disease.

## References

[1] Corey-Bloom J. The ABC of Alzheimer's disease: cognitive changes and their management in Alzheimer's disease and related dementias. Int Psychogeriatr. (2002) 14(Suppl. 1):51–75. 10.1017/S1041610203008664 12636180

[2] Kumar A Sidhu J Goyal A Tsao JW. Alzheimer Disease. Treasure Island, FL: StatPearls Publishing (2022).

[3] De-Paula VJ Radanovic M Diniz BS Forlenza OV. Alzheimer's disease. Subcell Biochem. (2012) 65:329–52. 10.1007/978-94-007-5416-4_14 23225010

[4] Billings LM Oddo S Green KN McGaugh JL LaFerla FM. Intraneuronal Aβ causes the onset of early Alzheimer's disease-related cognitive deficits in transgenic mice. Neuron. (2005) 45:675–88. 10.1016/j.neuron.2005.01.040 15748844

[5] Bondi MW Edmonds EC Salmon DP. Alzheimer's disease: past, present, and future. J Int Neuropsychol Soc. (2017) 23:818–31. 10.1017/S135561771700100X 29198280 PMC5830188

[6] Möller HJ Graeber MB. The case described by Alois Alzheimer in 1911. Historical and conceptual perspectives based on the clinical record and neurohistological sections. Eur Arch Psychiatry Clin Neurosci. (1998) 248:111–22. 10.1007/s004060050027 9728729

[7] Barnett R. Alzheimer's disease. Lancet. (2019) 393:1589. 10.1016/S0140-6736(19)30851-7 31007191

[8] Glenner GG Wong CW. Alzheimer's disease: initial report of the purification and characterization of a novel cerebrovascular amyloid protein. Biochem Biophys Res Commun. (1984) 120:885–90. 10.1016/S0006-291X(84)80190-4 6375662

[9] KoSIK KS Joachim CL Selkoe DJ. Microtubule-associated protein tau (tau) is a major antigenic component of paired helical filaments in Alzheimer disease. Proc Nat Acad Sci USA. (1986) 83:4044–8. 10.1073/pnas.83.11.4044 2424016 PMC323662

[10] Grundke-Iqbal I Iqbal K Quinlan M Tung Y-C Zaidi MS Wisniewski HM. Microtubule-associated protein tau. A component of Alzheimer paired helical filaments. J Biol Chem. (1986) 261:6084–9. 10.1016/S0021-9258(17)38495-8 3084478

[11] Gracon SI Knapp MJ Berghoff WG Pierce M DeJong R Lobbestael SJ. Safety of Tacrine: clinical trials, treatment IND, and postmarketing experience. Alzheimer Dis Assoc Disord. (1998) 12:93–101. 10.1097/00002093-199806000-00007 9651138

[12] McKhann GM Knopman DS Chertkow H Hyman BT Jack Jr CR Kawas CH. The diagnosis of dementia due to Alzheimer's disease: recommendations from the National Institute on Aging-Alzheimer's Association workgroups on diagnostic guidelines for Alzheimer's disease. Alzheimers Dement. (2011) 7:263–9. 10.1016/j.jalz.2011.03.005 21514250 PMC3312024

[13] McKhann G Drachman D Folstein M Katzman R Price D Stadlan EM. Clinical diagnosis of Alzheimer's disease: report of the NINCDS-ADRDA Work Group under the auspices of Department of Health and Human Services Task Force on Alzheimer's Disease. Neurology. (1984) 34:939–44. 10.1212/WNL.34.7.939 6610841

[14] Jack Jr CR Bennett DA Blennow K Carrillo MC Dunn B Haeberlein SB. NIA-AA research framework: toward a biological definition of Alzheimer's disease. Alzheimers Dement. (2018) 14:535–62. 10.1016/j.jalz.2018.02.018 29653606 PMC5958625

[15] Sperling RA Aisen PS Beckett LA Bennett DA Craft S Fagan AM. Toward defining the preclinical stages of Alzheimer's disease: recommendations from the National Institute on Aging-Alzheimer's Association workgroups on diagnostic guidelines for Alzheimer's disease. Alzheimers Dement. (2011) 7:280–92. 10.1016/j.jalz.2011.03.003 21514248 PMC3220946

[16] Jack CR Jr Andrews JS Beach TG Buracchio T Dunn B Graf A. Revised criteria for diagnosis and staging of Alzheimer's disease: Alzheimer's Association Workgroup. Alzheimers Dement. (2024) 20:5143–69. 10.1002/alz.13859 38934362 PMC11350039

[17] Sloane PD Zimmerman S Suchindran C Reed P Wang L Boustani M. The public health impact of Alzheimer's disease, 2000–2050: potential implication of treatment advances. Annu Rev Public Health. (2002) 23:213–31. 10.1146/annurev.publhealth.23.100901.140525 11910061

[18] Tahami Monfared AA Byrnes MJ White LA Zhang Q. Alzheimer's disease: epidemiology and clinical progression. Neurol Therapy. (2022) 11:553–69. 10.1007/s40120-022-00338-8 35286590 PMC9095793

[19] Cuyvers E Sleegers K. Genetic variations underlying Alzheimer's disease: evidence from genome-wide association studies and beyond. Lancet Neurol. (2016) 15:857–68. 10.1016/S1474-4422(16)00127-7 27302364

[20] Munoz DG Feldman H. Causes of Alzheimer's disease. CMAJ. (2000) 162:65–72. 11216203 PMC1232234

[21] Cacace R Sleegers K Van Broeckhoven C. Molecular genetics of early-onset Alzheimer's disease revisited. Alzheimers Dement. (2016) 12:733–48. 10.1016/j.jalz.2016.01.012 27016693

[22] Wingo TS Lah JJ Levey AI Cutler DJ. Autosomal recessive causes likely in early-onset Alzheimer disease. Arch Neurol. (2012) 69:59–64. 10.1001/archneurol.2011.221 21911656 PMC3332307

[23] Alonso Vilatela ME López-López M Yescas-Gómez P. Genetics of Alzheimer's disease. Arch Med Res. (2012) 43:622–31. 10.1016/j.arcmed.2012.10.017 23142261

[24] Bekris LM Yu CE Bird TD Tsuang DW. Genetics of Alzheimer disease. J Geriatr Psychiatry Neurol. (2010) 23:213–27. 10.1177/0891988710383571 21045163 PMC3044597

[25] Tanzi RE Bertram L. Twenty years of the Alzheimer's disease amyloid hypothesis: a genetic perspective. Cell. (2005) 120:545–55. 10.1016/j.cell.2005.02.008 15734686

[26] Thompson M. Thompson and Thompson Genetics in Medicine (1986).

[27] Hardy JA Higgins GA. Alzheimer's disease: the amyloid cascade hypothesis. Science. (1992) 256:184–5. 10.1126/science.1566067 1566067

[28] Doran E Keator D Head E Phelan MJ Kim R Totoiu M. Down syndrome, partial trisomy 21, and absence of Alzheimer's disease: the role of APP. J Alzheimers Dis. (2017) 56:459–70. 10.3233/JAD-160836 27983553 PMC5662115

[29] Van Cauwenberghe C Van Broeckhoven C Sleegers K. The genetic landscape of Alzheimer disease: clinical implications and perspectives. Genet Med. (2016) 18:421–30. 10.1038/gim.2015.117 26312828 PMC4857183

[30] Jonsson T Atwal JK Steinberg S Snaedal J Jonsson PV Bjornsson S. A mutation in APP protects against Alzheimer's disease and age-related cognitive decline. Nature. (2012) 488:96–9. 10.1038/nature11283 22801501

[31] Giri M Zhang M Lü Y. Genes associated with Alzheimer's disease: an overview and current status. Clin Interv Aging. (2016) 11:665–81. 10.2147/CIA.S105769 27274215 PMC4876682

[32] Cruts M Theuns J Van Broeckhoven C. Locus-specific mutation databases for neurodegenerative brain diseases. Hum Mutat. (2012) 33:1340–4. 10.1002/humu.22117 22581678 PMC3465795

[33] Dai MH Zheng H Zeng LD Zhang Y. The genes associated with early-onset Alzheimer's disease. Oncotarget. (2018) 9:15132–43. 10.18632/oncotarget.23738 29599933 PMC5871104

[34] Hoogmartens J Cacace R Van Broeckhoven C. Insight into the genetic etiology of Alzheimer's disease: a comprehensive review of the role of rare variants. Alzheimers Dement. (2021) 13:e12155. 10.1002/dad2.12155 33665345 PMC7896636

[35] Saunders AM Strittmatter WJ Schmechel D George-Hyslop PH Pericak-Vance MA Joo SH. Association of apolipoprotein E allele epsilon 4 with late-onset familial and sporadic Alzheimer's disease. Neurology. (1993) 43:1467–72. 10.1212/WNL.43.8.1467 8350998

[36] Strittmatter WJ Saunders AM Schmechel D Pericak-Vance M Enghild J Salvesen GS. Apolipoprotein E: high-avidity binding to beta-amyloid and increased frequency of type 4 allele in late-onset familial Alzheimer disease. Proc Natl Acad Sci USA. (1993) 90:1977–81. 10.1073/pnas.90.5.1977 8446617 PMC46003

[37] Pericak-Vance MA Bebout JL Gaskell PC Jr Yamaoka LH Hung WY Alberts MJ. Linkage studies in familial Alzheimer disease: evidence for chromosome 19 linkage. Am J Hum Genet. (1991) 48:1034–50. 2035524 PMC1683100

[38] Arboleda-Velasquez JF Lopera F O'Hare M Delgado-Tirado S Marino C Chmielewska N. Resistance to autosomal dominant Alzheimer's disease in an APOE3 Christchurch homozygote: a case report. Nat Med. (2019) 25:1680–3. 10.1038/s41591-019-0611-3 31686034 PMC6898984

[39] Farrer LA Cupples LA Haines JL Hyman B Kukull WA Mayeux R. Effects of age, sex, and ethnicity on the association between apolipoprotein E genotype and Alzheimer disease. A meta-analysis APOE and Alzheimer Disease Meta Analysis Consortium. JAMA. (1997) 278:1349–56. 10.1001/jama.278.16.1349 9343467

[40] Corder EH Saunders AM Risch NJ Strittmatter WJ Schmechel DE Gaskell PC Jr. Protective effect of apolipoprotein E type 2 allele for late onset Alzheimer disease. Nat Genet. (1994) 7:180–4. 10.1038/ng0694-180 7920638

[41] Gerdes LU Jeune B Ranberg KA Nybo H Vaupel JW. Estimation of apolipoprotein E genotype-specific relative mortality risks from the distribution of genotypes in centenarians and middle-aged men: apolipoprotein E gene is a “frailty gene,” not a “longevity gene”. Genet Epidemiol. (2000) 19:202–10. 10.1002/1098-2272(200010)19:3<202::AID-GEPI2>3.3.CO;2-H 11015124

[42] Schmidt S Barcellos LF DeSombre K Rimmler JB Lincoln RR Bucher P. Association of polymorphisms in the apolipoprotein E region with susceptibility to and progression of multiple sclerosis. Am J Hum Genet. (2002) 70:708–17. 10.1086/339269 11836653 PMC384947

[43] Lill CM Roehr JT McQueen MB Kavvoura FK Bagade S Schjeide BM. Comprehensive research synopsis and systematic meta-analyses in Parkinson's disease genetics: The PDGene database. PLoS Genet. (2012) 8:e1002548. 10.1371/journal.pgen.1002548 22438815 PMC3305333

[44] Bojanowski CM Shen D Chew EY Ning B Csaky KG Green WR. An apolipoprotein E variant may protect against age-related macular degeneration through cytokine regulation. Environ Mol Mutagen. (2006) 47:594–602. 10.1002/em.20233 16823865 PMC1899525

[45] Peck G Smeeth L Whittaker J Casas JP Hingorani A Sharma P. The genetics of primary haemorrhagic stroke, subarachnoid haemorrhage and ruptured intracranial aneurysms in adults. PLoS ONE. (2008) 3:e3691. 10.1371/journal.pone.0003691 19008959 PMC2579487

[46] Willer CJ Sanna S Jackson AU Scuteri A Bonnycastle LL Clarke R. Newly identified loci that influence lipid concentrations and risk of coronary artery disease. Nat Genet. (2008) 40:161–9. 10.1038/ng.76 18193043 PMC5206900

[47] Sebastiani P Solovieff N Dewan AT Walsh KM Puca A Hartley SW. Genetic signatures of exceptional longevity in humans. PLoS ONE. (2012) 7:e29848. 10.1371/journal.pone.0029848 22279548 PMC3261167

[48] Bateman RJ Aisen PS De Strooper B Fox NC Lemere CA Ringman JM. Autosomal-dominant Alzheimer's disease: a review and proposal for the prevention of Alzheimer's disease. Alzheimers Res Therapy. (2011) 3:1–13. 10.1186/alzrt59 21211070 PMC3109410

[49] Roses AD. An inherited variable poly-T repeat genotype in TOMM40 in Alzheimer disease. Arch Neurol. (2010) 67:536–41. 10.1001/archneurol.2010.88 20457951 PMC3140162

[50] Campion D Dumanchin C Hannequin D Dubois B Belliard S Puel M. Early-onset autosomal dominant Alzheimer disease: prevalence, genetic heterogeneity, and mutation spectrum. Am J Hum Genet. (1999) 65:664–70. 10.1086/302553 10441572 PMC1377972

[51] Avramopoulos D. Genetics of Alzheimer's disease: recent advances. Genome Med. (2009) 1:1–7. 10.1186/gm34 19341505 PMC2664945

[52] Durazzo TC Meyerhoff DJ Nixon SJ. Chronic cigarette smoking: implications for neurocognition and brain neurobiology. Int J Environ Res Public Health. (2010) 7:3760–91. 10.3390/ijerph7103760 21139859 PMC2996190

[53] Swan GE Lessov-Schlaggar CN. The effects of tobacco smoke and nicotine on cognition and the brain. Neuropsychol Rev. (2007) 17:259–73. 10.1007/s11065-007-9035-9 17690985

[54] Durazzo TC Mattsson N Weiner MW Initiative AsDN. Smoking and increased Alzheimer's disease risk: a review of potential mechanisms. Alzheimers Dement. (2014) 10:S122–45. 10.1016/j.jalz.2014.04.009 24924665 PMC4098701

[55] Barnes DE Yaffe K. The projected effect of risk factor reduction on Alzheimer's disease prevalence. Lancet Neurol. (2011) 10:819–28. 10.1016/S1474-4422(11)70072-2 21775213 PMC3647614

[56] Chang CC Zhao Y Lee CW Ganguli M. Smoking, death, and Alzheimer disease: a case of competing risks. Alzheimer Dis Assoc Disord. (2012) 26:300–6. 10.1097/WAD.0b013e3182420b6e 22185783 PMC3321062

[57] Bates M Noiton D. The chemical constituents in cigarettes and cigarette smoke: priorities for harm reduction. Priorities Harm Reduct. (2000). Available at: https://citeseerx.ist.psu.edu/document?repid=rep1&type=pdf&doi=d0a86a957bf1773af522a300725c017704bab34b

[58] Khandelwal PJ Herman AM Moussa CE. Inflammation in the early stages of neurodegenerative pathology. J Neuroimmunol. (2011) 238:1–11. 10.1016/j.jneuroim.2011.07.002 21820744 PMC3176984

[59] Northrop-Clewes CA Thurnham DI. Monitoring micronutrients in cigarette smokers. Clin Chim Acta. (2007) 377:14–38. 10.1016/j.cca.2006.08.028 17045981

[60] Sutherland GT Chami B Youssef P Witting PK. Oxidative stress in Alzheimer's disease: primary villain or physiological by-product? Redox Rep. (2013) 18:134–41. 10.1179/1351000213Y.0000000052 23849337 PMC6837641

[61] Giunta B Deng J Jin J Sadic E Rum S Zhou H. Evaluation of how cigarette smoke is a direct risk factor for Alzheimer's disease. Technol Innov. (2012) 14:39–48. 10.3727/194982412X13378627621752 22997546 PMC3445032

[62] Shi L Chen SJ Ma MY Bao YP Han Y Wang YM. Sleep disturbances increase the risk of dementia: a systematic review and meta-analysis. Sleep Med Rev. (2018) 40:4–16. 10.1016/j.smrv.2017.06.010 28890168

[63] Tranah GJ Blackwell T Stone KL Ancoli-Israel S Paudel ML Ensrud KE. Circadian activity rhythms and risk of incident dementia and mild cognitive impairment in older women. Ann Neurol. (2011) 70:722–32. 10.1002/ana.22468 22162057 PMC3244839

[64] Van Egroo M Narbutas J Chylinski D Villar González P Maquet P Salmon E. Sleep-wake regulation and the hallmarks of the pathogenesis of Alzheimer's disease. Sleep. (2019) 42:zsz017. 10.1093/sleep/zsz017 30649520

[65] Mander BA. Local sleep and Alzheimer's disease pathophysiology. Front Neurosci. (2020) 14:525970. 10.3389/fnins.2020.525970 33071726 PMC7538792

[66] Tang M Wu L Shen Z Chen J Yang Y Zhang M. Association between sleep and Alzheimer's disease: a bibliometric analysis from 2003 to 2022. Neuroepidemiology. (2023) 57:377–90. 10.1159/000533700 37699365

[67] Winer JR Deters KD Kennedy G Jin M Goldstein-Piekarski A Poston KL. Association of short and long sleep duration with amyloid-β burden and cognition in aging. JAMA Neurol. (2021) 78:1187–96. 10.1001/jamaneurol.2021.2876 34459862 PMC8406215

[68] Wang C Holtzman DM. Bidirectional relationship between sleep and Alzheimer's disease: role of amyloid, tau, and other factors. Neuropsychopharmacology. (2020) 45:104–20. 10.1038/s41386-019-0478-5 31408876 PMC6879647

[69] Lucey BP McCullough A Landsness EC Toedebusch CD McLeland JS Zaza AM. Reduced non-rapid eye movement sleep is associated with tau pathology in early Alzheimer's disease. Sci Transl Med. (2019) 11:aau6550. 10.1126/scitranslmed.aau6550 30626715 PMC6342564

[70] Irwin MR Vitiello MV. Implications of sleep disturbance and inflammation for Alzheimer's disease dementia. Lancet Neurol. (2019) 18:296–306. 10.1016/S1474-4422(18)30450-2 30661858

[71] Xiao SY Liu YJ Lu W Sha ZW Xu C Yu ZH. Possible neuropathology of sleep disturbance linking to Alzheimer's disease: astrocytic and microglial roles. Front Cell Neurosci. (2022) 16:875138. 10.3389/fncel.2022.875138 35755779 PMC9218054

[72] Hablitz LM Nedergaard M. The glymphatic system. Curr Biol. (2021) 31:R1371–5. 10.1016/j.cub.2021.08.026 34699796

[73] Xie L Kang H Xu Q Chen MJ Liao Y Thiyagarajan M. Sleep drives metabolite clearance from the adult brain. Science. (2013) 342:373–7. 10.1126/science.1241224 24136970 PMC3880190

[74] Saeedi M Rashidy-Pour A. Association between chronic stress and Alzheimer's disease: therapeutic effects of Saffron. Biomed Pharmacother. (2021) 133:110995. 10.1016/j.biopha.2020.110995 33232931

[75] McEwen BS. Effects of adverse experiences for brain structure and function. Biol Psychiatry. (2000) 48:721–31. 10.1016/S0006-3223(00)00964-1 11063969

[76] Magarin A McEwen B. Stress-induced atrophy of apical dendrites of hippocampal CA3c neurons: involvement of glucocorticoid secretion and excitatory amino acid receptors. Neuroscience. (1995) 69:89–98. 10.1016/0306-4522(95)00259-L 8637636

[77] Conrad CD McLaughlin KJ Harman JS Foltz C Wieczorek L Lightner E. Chronic glucocorticoids increase hippocampal vulnerability to neurotoxicity under conditions that produce CA3 dendritic retraction but fail to impair spatial recognition memory. J Neurosci. (2007) 27:8278–85. 10.1523/JNEUROSCI.2121-07.2007 17670974 PMC1989144

[78] Gould E McEwen BS Tanapat P Galea LA Fuchs E. Neurogenesis in the dentate gyrus of the adult tree shrew is regulated by psychosocial stress and NMDA receptor activation. J Neurosci. (1997) 17:2492–8. 10.1523/JNEUROSCI.17-07-02492.1997 9065509 PMC6573503

[79] Conrad CD. A critical review of chronic stress effects on spatial learning and memory. Progr Neuropsychopharmacol Biol Psychiatry. (2010) 34:742–55. 10.1016/j.pnpbp.2009.11.003 19903505

[80] McEwen BS. Plasticity of the hippocampus: adaptation to chronic stress and allostatic load. Ann N Y Acad Sci. (2001) 933:265–77. 10.1111/j.1749-6632.2001.tb05830.x 12000027

[81] Marin M-F Lord C Andrews J Juster R-P Sindi S Arsenault-Lapierre G. Chronic stress, cognitive functioning and mental health. Neurobiol Learn Mem. (2011) 96:583–95. 10.1016/j.nlm.2011.02.016 21376129

[82] Sotiropoulos I Catania C Pinto LG Silva R Pollerberg GE Takashima A. Stress acts cumulatively to precipitate Alzheimer's disease-like tau pathology and cognitive deficits. J Neurosci. (2011) 31:7840–7. 10.1523/JNEUROSCI.0730-11.2011 21613497 PMC6633145

[83] Green KN Billings LM Roozendaal B McGaugh JL LaFerla FM. Glucocorticoids increase amyloid-β and tau pathology in a mouse model of Alzheimer's disease. J Neurosci. (2006) 26:9047–56. 10.1523/JNEUROSCI.2797-06.2006 16943563 PMC6675335

[84] Rolland Y Abellan van Kan G Vellas B. Physical activity and Alzheimer's disease: from prevention to therapeutic perspectives. J Am Med Dir Assoc. (2008) 9:390–405. 10.1016/j.jamda.2008.02.007 18585641

[85] Ahlskog JE Geda YE Graff-Radford NR Petersen RC. Physical exercise as a preventive or disease-modifying treatment of dementia and brain aging. Mayo Clin Proc. (2011) 86:876–84. 10.4065/mcp.2011.0252 21878600 PMC3258000

[86] Vemuri P Knopman DS Lesnick TG Przybelski SA Mielke MM Graff-Radford J. Evaluation of amyloid protective factors and Alzheimer disease neurodegeneration protective factors in elderly individuals. JAMA Neurol. (2017) 74:718–26. 10.1001/jamaneurol.2017.0244 28418521 PMC5649401

[87] Livingston G Sommerlad A Orgeta V Costafreda SG Huntley J Ames D. Dementia prevention, intervention, and care. Lancet. (2017) 390:2673–734. 10.1016/S0140-6736(17)31363-6 28735855

[88] Hamer M Chida Y. Physical activity and risk of neurodegenerative disease: a systematic review of prospective evidence. Psychol Med. (2009) 39:3–11. 10.1017/S0033291708003681 18570697

[89] Zheng G Xia R Zhou W Tao J Chen L. Aerobic exercise ameliorates cognitive function in older adults with mild cognitive impairment: a systematic review and meta-analysis of randomised controlled trials. Br J Sports Med. (2016) 50:1443–50. 10.1136/bjsports-2015-095699 27095745

[90] Heyn P Abreu BC Ottenbacher KJ. The effects of exercise training on elderly persons with cognitive impairment and dementia: a meta-analysis. Arch Phys Med Rehabil. (2004) 85:1694–704. 10.1016/j.apmr.2004.03.019 15468033

[91] Deslandes A Moraes H Ferreira C Veiga H Silveira H Mouta R. Exercise and mental health: many reasons to move. Neuropsychobiology. (2009) 59:191–8. 10.1159/000223730 19521110

[92] Eggermont L Swaab D Luiten P Scherder E. Exercise, cognition and Alzheimer's disease: more is not necessarily better. Neurosci Biobehav Rev. (2006) 30:562–75. 10.1016/j.neubiorev.2005.10.004 16359729

[93] Van Praag H Christie BR Sejnowski TJ Gage FH. Running enhances neurogenesis, learning, and long-term potentiation in mice. Proc Nat Acad Sci USA. (1999) 96:13427–31. 10.1073/pnas.96.23.13427 10557337 PMC23964

[94] Farmer J Zhao X Van Praag H Wodtke K Gage F Christie B. Effects of voluntary exercise on synaptic plasticity and gene expression in the dentate gyrus of adult male Sprague–Dawley rats *in vivo*. Neuroscience. (2004) 124:71–9. 10.1016/j.neuroscience.2003.09.029 14960340

[95] Erickson KI Voss MW Prakash RS Basak C Szabo A Chaddock L. Exercise training increases size of hippocampus and improves memory. Proc Nat Acad Sci USA. (2011) 108:3017–22. 10.1073/pnas.1015950108 21282661 PMC3041121

[96] Tseng BY Uh J Rossetti HC Cullum CM Diaz-Arrastia RF Levine BD. Masters athletes exhibit larger regional brain volume and better cognitive performance than sedentary older adults. J Magn Reson Imaging. (2013) 38:1169–76. 10.1002/jmri.24085 23908143 PMC3812419

[97] Yoon DH Kang D Kim H Kim JS Song HS Song W. Effect of elastic band-based high-speed power training on cognitive function, physical performance and muscle strength in older women with mild cognitive impairment. Geriatr Gerontol Int. (2017) 17:765–72. 10.1111/ggi.12784 27396580

[98] Liang KY Mintun MA Fagan AM Goate AM Bugg JM Holtzman DM. Exercise and Alzheimer's disease biomarkers in cognitively normal older adults. Ann Neurol. (2010) 68:311–8. 10.1002/ana.22096 20818789 PMC2936720

[99] Baker LD Frank LL Foster-Schubert K Green PS Wilkinson CW McTiernan A. Effects of aerobic exercise on mild cognitive impairment: a controlled trial. Arch Neurol. (2010) 67:71–9. 10.1001/archneurol.2009.307 20065132 PMC3056436

[100] Adlard PA Perreau VM Pop V Cotman CW. Voluntary exercise decreases amyloid load in a transgenic model of Alzheimer's disease. J Neurosci. (2005) 25:4217–21. 10.1523/JNEUROSCI.0496-05.2005 15858047 PMC6725122

[101] Tapia-Rojas C Aranguiz F Varela-Nallar L Inestrosa NC. Voluntary running attenuates memory loss, decreases neuropathological changes and induces neurogenesis in a mouse model of a Lzheimer's disease. Brain Pathol. (2016) 26:62–74. 10.1111/bpa.12255 25763997 PMC8029165

[102] Ohia-Nwoko O Montazari S Lau Y-S Eriksen JL. Long-term treadmill exercise attenuates tau pathology in P301S tau transgenic mice. Mol Neurodegener. (2014) 9:1–17. 10.1186/1750-1326-9-54 25432085 PMC4280713

[103] Belarbi K Burnouf S Fernandez-Gomez F-J Laurent C Lestavel S Figeac M. Beneficial effects of exercise in a transgenic mouse model of Alzheimer's disease-like Tau pathology. Neurobiol Dis. (2011) 43:486–94. 10.1016/j.nbd.2011.04.022 21569847

[104] De la Rosa A Olaso-Gonzalez G Arc-Chagnaud C Millan F Salvador-Pascual A García-Lucerga C. Physical exercise in the prevention and treatment of Alzheimer's disease. J Sport Health Sci. (2020) 9:394–404. 10.1016/j.jshs.2020.01.004 32780691 PMC7498620

[105] Edwards GA III Gamez N Escobedo G Jr Calderon O Moreno-Gonzalez I. Modifiable risk factors for Alzheimer's disease. Front Aging Neurosci. (2019) 11:146. 10.3389/fnagi.2019.00146 31293412 PMC6601685

[106] Pitkälä KH Pöysti MM Laakkonen M-L Tilvis RS Savikko N Kautiainen H. Effects of the Finnish Alzheimer disease exercise trial (FINALEX): a randomized controlled trial. JAMA Intern Med. (2013) 173:894–901. 10.1001/jamainternmed.2013.359 23589097

[107] Rolland Y Pillard F Klapouszczak A Reynish E Thomas D Andrieu S. Exercise program for nursing home residents with Alzheimer's disease: a 1-year randomized, controlled trial. J Am Geriatr Soc. (2007) 55:158–65. 10.1111/j.1532-5415.2007.01035.x 17302650

[108] Vidoni ED Van Sciver A Johnson DK He J Honea R Haines B. A community-based approach to trials of aerobic exercise in aging and Alzheimer's disease. Contemp Clin Trials. (2012) 33:1105–16. 10.1016/j.cct.2012.08.002 22903151 PMC3468654

[109] Morris JK Vidoni ED Johnson DK Van Sciver A Mahnken JD Honea RA. Aerobic exercise for Alzheimer's disease: a randomized controlled pilot trial. PLoS ONE. (2017) 12:e0170547. 10.1371/journal.pone.0170547 28187125 PMC5302785

[110] Baumgart M Snyder HM Carrillo MC Fazio S Kim H Johns H. Summary of the evidence on modifiable risk factors for cognitive decline and dementia: a population-based perspective. Alzheimers Dement. (2015) 11:718–26. 10.1016/j.jalz.2015.05.016 26045020

[111] Wiegmann C Mick I Brandl EJ Heinz A Gutwinski S. Alcohol and dementia - what is the link? A systematic review. Neuropsychiatr Dis Treat. (2020) 16:87–99. 10.2147/NDT.S198772 32021202 PMC6957093

[112] Beydoun MA Beydoun HA Gamaldo AA Teel A Zonderman AB Wang Y. Epidemiologic studies of modifiable factors associated with cognition and dementia: systematic review and meta-analysis. BMC Public Health. (2014) 14:643. 10.1186/1471-2458-14-643 24962204 PMC4099157

[113] Chan KK Chiu KC Chu LW. Association between alcohol consumption and cognitive impairment in Southern Chinese older adults. Int J Geriatr Psychiatry. (2010) 25:1272–9. 10.1002/gps.2470 21086537

[114] Jeon KH Han K Jeong S-M Park J Yoo JE Yoo J. Changes in alcohol consumption and risk of dementia in a nationwide cohort in South Korea. JAMA Netw Open. (2023) 6:e2254771. 10.1001/jamanetworkopen.2022.54771 36745453 PMC12549098

[115] Heymann D Stern Y Cosentino S Tatarina-Nulman O Dorrejo JN Gu Y. The association between alcohol use and the progression of Alzheimer's disease. Curr Alzheimer Res. (2016) 13:1356–62. 10.2174/1567205013666160603005035 27628432 PMC5526221

[116] Letenneur L Larrieu S Barberger-Gateau P. Alcohol and tobacco consumption as risk factors of dementia: a review of epidemiological studies. Biomed Pharmacother. (2004) 58:95–9. 10.1016/j.biopha.2003.12.004 14992790

[117] Dhouafli Z Cuanalo-Contreras K Hayouni EA Mays CE Soto C Moreno-Gonzalez I. Inhibition of protein misfolding and aggregation by natural phenolic compounds. Cell Mol Life Sci. (2018) 75:3521–38. 10.1007/s00018-018-2872-2 30030591 PMC11105286

[118] Chandrashekar DV Steinberg RA Han D Sumbria RK. Alcohol as a modifiable risk factor for Alzheimer's disease—evidence from experimental studies. Int J Mol Sci. (2023) 24:9492. 10.3390/ijms24119492 37298443 PMC10253673

[119] Rehm J Hasan OSM Black SE Shield KD Schwarzinger M. Alcohol use and dementia: a systematic scoping review. Alzheimers Res Ther. (2019) 11:1. 10.1186/s13195-018-0453-0 30611304 PMC6320619

[120] Schwarzinger M Pollock BG Hasan OSM Dufouil C Rehm J. Contribution of alcohol use disorders to the burden of dementia in France 2008-13: a nationwide retrospective cohort study. Lancet Public Health. (2018) 3:e124–32. 10.1016/S2468-2667(18)30022-7 29475810

[121] Weissenborn R Duka T. Acute alcohol effects on cognitive function in social drinkers: their relationship to drinking habits. Psychopharmacology. (2003) 165:306–12. 10.1007/s00213-002-1281-1 12439627

[122] Topiwala A Allan CL Valkanova V Zsoldos E Filippini N Sexton C. Moderate alcohol consumption as risk factor for adverse brain outcomes and cognitive decline: longitudinal cohort study. BMJ. (2017) 357:j2353. 10.1136/bmj.j2353 28588063 PMC5460586

[123] Fernandez GM Savage LM. Adolescent binge ethanol exposure alters specific forebrain cholinergic cell populations and leads to selective functional deficits in the prefrontal cortex. Neuroscience. (2017) 361:129–43. 10.1016/j.neuroscience.2017.08.013 28807788 PMC5609857

[124] Vetreno RP Crews FT. Adolescent binge ethanol-induced loss of basal forebrain cholinergic neurons and neuroimmune activation are prevented by exercise and indomethacin. PLoS ONE. (2018) 13:e0204500. 10.1371/journal.pone.0204500 30296276 PMC6175501

[125] Lundgaard I Wang W Eberhardt A Vinitsky HS Reeves BC Peng S. Beneficial effects of low alcohol exposure, but adverse effects of high alcohol intake on glymphatic function. Sci Rep. (2018) 8:2246. 10.1038/s41598-018-20424-y 29396480 PMC5797082

[126] Toda A Tagata Y Nakada T Komatsu M Shibata N Arai H. Changes in Mini-Mental State Examination score in Alzheimer's disease patients after stopping habitual drinking. Psychogeriatrics. (2013) 13:94–8. 10.1111/psyg.12008 23909966

[127] Kivipelto M Ngandu T Fratiglioni L Viitanen M Kåreholt I Winblad B. Obesity and vascular risk factors at midlife and the risk of dementia and Alzheimer disease. Arch Neurol. (2005) 62:1556–60. 10.1001/archneur.62.10.1556 16216938

[128] Wolf PA Beiser A Elias MF Au R Vasan RS Seshadri S. Relation of obesity to cognitive function: importance of central obesity and synergistic influence of concomitant hypertension. The Framingham Heart Study. Curr Alzheimer Res. (2007) 4:111–6. 10.2174/156720507780362263 17430232

[129] Fitzpatrick AL Kuller LH Lopez OL Diehr P O'Meara ES Longstreth WT Jr. Midlife and late-life obesity and the risk of dementia: cardiovascular health study. Arch Neurol. (2009) 66:336–42. 10.1001/archneurol.2008.582 19273752 PMC3513375

[130] Gustafson DR Bäckman K Waern M Ostling S Guo X Zandi P. Adiposity indicators and dementia over 32 years in Sweden. Neurology. (2009) 73:1559–66. 10.1212/WNL.0b013e3181c0d4b6 19901247 PMC2777073

[131] Tolppanen AM Ngandu T Kåreholt I Laatikainen T Rusanen M Soininen H. Midlife and late-life body mass index and late-life dementia: results from a prospective population-based cohort. J Alzheimers Dis. (2014) 38:201–9. 10.3233/JAD-130698 23948937

[132] Singh-Manoux A Dugravot A Shipley M Brunner EJ Elbaz A Sabia S. Obesity trajectories and risk of dementia: 28 years of follow-up in the Whitehall II Study. Alzheimers Dement. (2018) 14:178–86. 10.1016/j.jalz.2017.06.2637 28943197 PMC5805839

[133] Kivimäki M Luukkonen R Batty GD Ferrie JE Pentti J Nyberg ST. Body mass index and risk of dementia: Analysis of individual-level data from 13 million individuals. Alzheimers Dement. (2018) 14:601–9. 10.1016/j.jalz.2017.09.016 29169013 PMC5948099

[134] Serrano-Pozo A Growdon JH. Is Alzheimer's disease risk modifiable? J Alzheimers Dis. (2019) 67:795–819. 10.3233/JAD181028 30776012 PMC6708279

[135] Hossain S Beydoun MA Kuczmarski MF Tajuddin S Evans MK Zonderman AB. The interplay of diet quality and Alzheimer's disease genetic risk score in relation to cognitive performance among Urban African Americans. Nutrients. (2019) 11:2181. 10.3390/nu11092181 31514322 PMC6769979

[136] de Sousa OV Mendes J Amaral T. Nutritional and functional indicators and their association with mortality among older adults with Alzheimer's disease. Am J Alzheimers Dis Other Dement. (2020) 35:1533317520907168. 10.1177/1533317520907168 32088972 PMC10624010

[137] Xu Lou I Ali K Chen Q. Effect of nutrition in Alzheimer's disease: a systematic review. Front Neurosci. (2023) 17:1147177. 10.3389/fnins.2023.1147177 37214392 PMC10194838

[138] Taylor MK Sullivan DK Morris JK Vidoni ED Honea RA Mahnken JD. High glycemic diet is related to brain amyloid accumulation over one year in preclinical Alzheimer's disease. Front Nutr. (2021) 8:741534. 10.3389/fnut.2021.741534 34646853 PMC8502814

[139] Gentreau M Chuy V Féart C Samieri C Ritchie K Raymond M. Refined carbohydrate-rich diet is associated with long-term risk of dementia and Alzheimer's disease in apolipoprotein E ε4 allele carriers. Alzheimers Dement. (2020) 16:1043–53. 10.1002/alz.12114 32506713

[140] Hoscheidt S Sanderlin AH Baker LD Jung Y Lockhart S Kellar D. Mediterranean and Western diet effects on Alzheimer's disease biomarkers, cerebral perfusion, and cognition in mid-life: a randomized trial. Alzheimers Dement. (2022) 18:457–68. 10.1002/alz.12421 34310044 PMC9207984

[141] Andreu-Reinón ME Chirlaque MD Gavrila D Amiano P Mar J Tainta M. Mediterranean diet and risk of dementia and Alzheimer's disease in the EPIC-Spain dementia cohort study. Nutrients. (2021) 13:700. 10.3390/nu13020700 33671575 PMC7927039

[142] Ballarini T. Melo van Lent D, Brunner J, Schröder A, Wolfsgruber S, Altenstein S, et al. Mediterranean diet, Alzheimer disease biomarkers, and brain atrophy in old age. Neurology. (2021) 96:e2920–32. 10.1212/WNL.0000000000012067 33952652 PMC8253566

[143] Nutaitis AC Tharwani SD Serra M Goldstein F Zhao L Sher S. Diet as a risk factor for cognitive decline in African Americans and Caucasians with a parental history of Alzheimer's disease: a cross-sectional pilot study dietary patterns. J Prev Alzheimers Dis. (2019) 6:50–5. 10.14283/jpad.2018.44 30569086 PMC6399991

[144] Anastasiou CA Yannakoulia M Kosmidis MH Dardiotis E Hadjigeorgiou GM Sakka P. Mediterranean diet and cognitive health: Initial results from the Hellenic Longitudinal Investigation of Ageing and Diet. PLoS ONE. (2017) 12:e0182048. 10.1371/journal.pone.0182048 28763509 PMC5538737

[145] Scarmeas N Stern Y Tang MX Mayeux R Luchsinger JA. Mediterranean diet and risk for Alzheimer's disease. Ann Neurol. (2006) 59:912–21. 10.1002/ana.20854 16622828 PMC3024594

[146] Panagiotakos DB Georgousopoulou EN Pitsavos C Chrysohoou C Skoumas I Pitaraki E. Exploring the path of Mediterranean diet on 10-year incidence of cardiovascular disease: the ATTICA study (2002–2012). Nutr Metab Cardiovasc Dis. (2015) 25:327–35. 10.1016/j.numecd.2014.09.006 25445882

[147] Scarmeas N Luchsinger JA Stern Y Gu Y He J DeCarli C. Mediterranean diet and magnetic resonance imaging–assessed cerebrovascular disease. Ann Neurol. (2011) 69:257–68. 10.1002/ana.22317 21387371 PMC3066080

[148] Scarmeas N Stern Y Mayeux R Luchsinger JA. Mediterranean diet, Alzheimer disease, and vascular mediation. Arch Neurol. (2006) 63:1709–17. 10.1001/archneur.63.12.noc60109 17030648 PMC3024906

[149] Berti V Walters M Sterling J Quinn CG Logue M Andrews R. Mediterranean diet and 3-year Alzheimer brain biomarker changes in middle-aged adults. Neurology. (2018) 90:e1789–98. 10.1212/WNL.0000000000005527 29653991 PMC5957301

[150] Gustafson DR Bäckman K Scarmeas N Stern Y Manly JJ Mayeux R. Dietary fatty acids and risk of Alzheimer's disease and related dementias: observations from the Washington Heights-Hamilton Heights-Inwood Columbia Aging Project (WHICAP). Alzheimers Dement. (2020) 16:1638–49. 10.1002/alz.12154 32715635 PMC8409226

[151] Chu C-S Hung C-F Ponnusamy VK Chen K-C Chen N-C. Higher serum DHA and slower cognitive decline in patients with Alzheimer's disease: two-year follow-up. Nutrients. (2022) 14:1159. 10.3390/nu14061159 35334816 PMC8950997

[152] Drinkwater E Davies C Spires-Jones TL. Potential neurobiological links between social isolation and Alzheimer's disease risk. Eur J Neurosci. (2022) 56:5397–412. 10.1111/ejn.15373 34184343

[153] House JS Landis KR Umberson D. Social relationships and health. Science. (1988) 241:540–5. 10.1126/science.3399889 3399889

[154] Read S Comas-Herrera A Grundy E. Social isolation and memory decline in later-life. J Gerontol B Psychol Sci Soc Sci. (2020) 75:367–76. 10.1093/geronb/gbz152 31781769 PMC6963696

[155] Cardona M Andrés P. Are social isolation and loneliness associated with cognitive decline in ageing? Front Aging Neurosci. (2023) 15:1075563. 10.3389/fnagi.2023.1075563 36909946 PMC9995915

[156] Cornwell EY Waite LJ. Social disconnectedness, perceived isolation, and health among older adults. J Health Soc Behav. (2009) 50:31–48. 10.1177/002214650905000103 19413133 PMC2756979

[157] Stern Y. Cognitive reserve in ageing and Alzheimer's disease. Lancet Neurol. (2012) 11:1006–12. 10.1016/S1474-4422(12)70191-6 23079557 PMC3507991

[158] Scarmeas N Levy G Tang MX Manly J Stern Y. Influence of leisure activity on the incidence of Alzheimer's disease. Neurology. (2001) 57:2236–42. 10.1212/WNL.57.12.2236 11756603 PMC3025284

[159] Stern Y Gurland B Tatemichi TK Tang MX Wilder D Mayeux R. Influence of education and occupation on the incidence of Alzheimer's disease. JAMA. (1994) 271:1004–10. 10.1001/jama.1994.03510370056032 8139057

[160] Evans IEM Llewellyn DJ Matthews FE Woods RT Brayne C Clare L. Social isolation, cognitive reserve, and cognition in healthy older people. PLoS ONE. (2018) 13:e0201008. 10.1371/journal.pone.0201008 30118489 PMC6097646

[161] Kaffashian S Dugravot A Elbaz A Shipley MJ Sabia S Kivimäki M. Predicting cognitive decline. Neurology. (2013) 80:1300–6. 10.1212/WNL.0b013e31828ab370 23547265 PMC3656460

[162] Unverzagt FW McClure LA Wadley VG Jenny NS Go RC Cushman M. Vascular risk factors and cognitive impairment in a stroke-free cohort. Neurology. (2011) 77:1729–36. 10.1212/WNL.0b013e318236ef23 22067959 PMC3208949

[163] Jefferson AL Hohman TJ Liu D Haj-Hassan S Gifford KA Benson EM. Adverse vascular risk is related to cognitive decline in older adults. J Alzheimers Dis. (2015) 44:1361–73. 10.3233/JAD-141812 25471188 PMC4336578

[164] Viticchi G Falsetti L Buratti L Boria C Luzzi S Bartolini M. Framingham risk score can predict cognitive decline progression in Alzheimer's disease. Neurobiol Aging. (2015) 36:2940–5. 10.1016/j.neurobiolaging.2015.07.023 26279114

[165] Sabia S Fayosse A Dumurgier J Schnitzler A Empana J-P Ebmeier KP. Association of ideal cardiovascular health at age 50 with incidence of dementia: 25 year follow-up of Whitehall II cohort study. BMJ. (2019) 366:l4414. 10.1136/bmj.l4414 31391187 PMC6664261

[166] Newman AB Fitzpatrick AL Lopez O Jackson S Lyketsos C Jagust W. Dementia and Alzheimer's disease incidence in relationship to cardiovascular disease in the cardiovascular health study cohort. J Am Geriatr Soc. (2005) 53:1101–7. 10.1111/j.1532-5415.2005.53360.x 16108925

[167] Noguchi-Shinohara M Yuki-Nozaki S Abe C Mori A Horimoto M Yokogawa M. Diabetes mellitus, elevated Hemoglobin A 1c, and glycated albumin are associated with the presence of all-cause dementia and Alzheimer's disease: the JPSC-AD Study. J Alzheimers Dis. (2022) 85:235–47. 10.3233/JAD-215153 34806607

[168] Marseglia A Fratiglioni L Kalpouzos G Wang R Bäckman L Xu W. Prediabetes and diabetes accelerate cognitive decline and predict microvascular lesions: a population-based cohort study. Alzheimers Dement. (2019) 15:25–33. 10.1016/j.jalz.2018.06.3060 30114414

[169] Norton S Matthews FE Barnes DE Yaffe K Brayne C. Potential for primary prevention of Alzheimer's disease: an analysis of population-based data. Lancet Neurol. (2014) 13:788–94. 10.1016/S1474-4422(14)70136-X 25030513

[170] Lacy ME Gilsanz P Karter AJ Quesenberry CP Pletcher MJ Whitmer RA. Long-term glycemic control and dementia risk in type 1 diabetes. Diabetes Care. (2018) 41:2339–45. 10.2337/dc18-0073 30181165 PMC6196833

[171] Habes M Jacobson AM Braffett BH Rashid T Ryan CM Shou H. Patterns of regional brain atrophy and brain aging in middle- and older-aged adults with type 1 diabetes. JAMA Netw Open. (2023) 6:e2316182. 10.1001/jamanetworkopen.2023.16182 37261829 PMC10236234

[172] Fernández Fernández R Martín JI Antón MAM. Depression as a risk factor for dementia: a meta-analysis. J Neuropsychiatry Clin Neurosci. (2024) 36:101–9. 10.1176/appi.neuropsych.20230043 38111332

[173] Diniz BS Butters MA Albert SM Dew MA Reynolds CF. Late-life depression and risk of vascular dementia and Alzheimer's disease: systematic review and meta-analysis of community-based cohort studies. Br J Psychiatry. (2018) 202:329–35. 10.1192/bjp.bp.112.118307 23637108 PMC3640214

[174] Ownby RL Crocco E Acevedo A John V Loewenstein D. Depression and risk for Alzheimer disease: systematic review, meta-analysis, and metaregression analysis. Arch Gen Psychiatry. (2006) 63:530–8. 10.1001/archpsyc.63.5.530 16651510 PMC3530614

[175] Mayeux R Chen J Mirabello E Marder K Bell K Dooneief G. An estimate of the incidence of dementia in idiopathic Parkinson's disease. Neurology. (1990) 40:1513. 10.1212/WNL.40.10.1513 2215941

[176] Biggins CA Boyd JL Harrop FM Madeley P Mindham RH Randall JI. A controlled, longitudinal study of dementia in Parkinson's disease. J Neurol Neurosurg Psychiatry. (1992) 55:566–71. 10.1136/jnnp.55.7.566 1640232 PMC489167

[177] Marder K Tang M-X Cote L Stern Y Mayeux R. The frequency and associated risk factors for dementia in patients with Parkinson's disease. Arch Neurol. (1995) 52:695–701. 10.1001/archneur.1995.00540310069018 7619026

[178] Mahieux F Fénelon G Flahault A Manifacier M-J Michelet D Boller F. Neuropsychological prediction of dementia in Parkinson's disease. J Neurol Neurosurg Psychiatry. (1998) 64:178–83. 10.1136/jnnp.64.2.178 9489527 PMC2169963

[179] Hughes TA Ross HF Musa S Bhattacherjee S Nathan RN Mindham RHS. A 10-year study of the incidence of and factors predicting dementia in Parkinson's disease. Neurology. (2000) 54:1596–603. 10.1212/WNL.54.8.1596 10762499

[180] Aarsland D Andersen K Larsen JP Lolk A Nielsen H. Kragh–Sørensen P. Risk of dementia in Parkinson's disease. Neurology. (2001) 56:730–6. 10.1212/WNL.56.6.730 11274306

[181] Hobson P Meara J. Risk and incidence of dementia in a cohort of older subjects with Parkinson's disease in the United Kingdom. Mov Disord. (2004) 19:1043–9. 10.1002/mds.20216 15372593

[182] Williams-Gray CH Foltynie T Brayne CEG Robbins TW Barker RA. Evolution of cognitive dysfunction in an incident Parkinson's disease cohort. Brain. (2007) 130:1787–98. 10.1093/brain/awm111 17535834

[183] Williams-Gray CH Evans JR Goris A Foltynie T Ban M Robbins TW. The distinct cognitive syndromes of Parkinson's disease: 5 year follow-up of the CamPaIGN cohort. Brain. (2009) 132:2958–69. 10.1093/brain/awp245 19812213

[184] Perez F Helmer C Foubert-Samier A Auriacombe S Dartigues J-F Tison F. Risk of dementia in an elderly population of Parkinson's disease patients: a 15-year population-based study. Alzheimers Dement. (2012) 8:463–9. 10.1016/j.jalz.2011.09.230 22651942

[185] Åström DO Simonsen J Raket LL Sgarbi S Hellsten J Hagell P. High risk of developing dementia in Parkinson's disease: a Swedish registry-based study. Sci Rep. (2022) 12:16759. 10.1038/s41598-022-21093-8 36202962 PMC9537530

[186] Bronnick K Emre M Lane R Tekin S Aarsland D. Profile of cognitive impairment in dementia associated with Parkinson's disease compared with Alzheimer's disease. J Neurol Neurosurg Psychiatry. (2007) 78:1064–8. 10.1136/jnnp.2006.108076 17287236 PMC2117535

[187] Kim JH Lee HS Kim YH Kwon MJ Kim J-H Min CY. The Association between thyroid diseases and alzheimer's disease in a National Health Screening Cohort in Korea. Front Endocrinol. (2022) 13:815063. 10.3389/fendo.2022.815063 35321339 PMC8936176

[188] Salehipour A Dolatshahi M Haghshomar M Amin J. The role of thyroid dysfunction in Alzheimer's disease: a systematic review and meta-analysis. J Prev Alzheimers Dis. (2023) 10:276–86. 10.14283/jpad.2023.20 36946455

[189] Breteler MMB Van Duijn CM Chandra V Fratiglioni L Graves AB Heyman A. Medical history and the risk of Alzheimer's disease: a collaborative re-analysis of case-control studies. Int J Epidemiol. (1991) 20(Supplement_2):S36–42. 10.1093/ije/20.Supplement_2.S36 1833352

[190] Wieland DR Wieland JR Wang H Chen Y-H Lin C-H Wang J-J. Thyroid disorders and dementia risk. Neurology. (2022) 99:e679–e87. 10.1212/WNL.0000000000200740 35794019

[191] Thvilum M Brandt F Lillevang-Johansen M Folkestad L Brix TH Hegedüs L. Increased risk of dementia in hypothyroidism: a Danish nationwide register-based study. Clin Endocrinol. (2021) 94:1017–24. 10.1111/cen.14424 33484007

[192] Tan ZS Beiser A Vasan RS Au R Auerbach S Kiel DP. Thyroid function and the risk of Alzheimer disease: The Framingham Study. Arch Intern Med. (2008) 168:1514–20. 10.1001/archinte.168.14.1514 18663163 PMC2694610

[193] Parsaik AK Singh B Roberts RO Pankratz S Edwards KK Geda YE. Hypothyroidism and risk of mild cognitive impairment in elderly persons: a population-based study. JAMA Neurol. (2014) 71:201–7. 10.1001/jamaneurol.2013.5402 24378475 PMC4136444

[194] Ma L-Y Zhao B Ou Y-N Zhang D-D Li Q-Y Tan L. Association of thyroid disease with risks of dementia and cognitive impairment: a meta-analysis and systematic review. Front Aging Neurosci. (2023) 15:1137584. 10.3389/fnagi.2023.1137584 36993905 PMC10040782

[195] Ye Y Wang Y Li S Guo J Ding L Liu M. Association of hypothyroidism and the risk of cognitive dysfunction: a meta-analysis. J Clin Med. (2022) 11:6726. 10.3390/jcm11226726 36431204 PMC9694203

[196] Kurella M Chertow GM Luan J Yaffe K. Cognitive impairment in chronic kidney disease. J Am Geriatr Soc. (2004) 52:1863–9. 10.1111/j.1532-5415.2004.52508.x 15507063

[197] Chandra A Dervenoulas G Politis M for the Alzheimer's Disease Neuroimaging I. Magnetic resonance imaging in Alzheimer's disease and mild cognitive impairment. J Neurol. (2019) 266:1293–302. 10.1007/s00415-018-9016-3 30120563 PMC6517561

[198] Helmer C Stengel B Metzger M Froissart M Massy Z-A Tzourio C. Chronic kidney disease, cognitive decline, and incident dementia. Neurology. (2011) 77:2043–51. 10.1212/WNL.0b013e31823b4765 22116945

[199] Kwon MJ Song YR Kim J-H Kim JH Kang HS Lim H. Exploring the link between chronic kidney disease and Alzheimer's disease: a longitudinal follow-up study using the Korean National Health Screening Cohort. Biomedicines. (2023) 11:1606. 10.3390/biomedicines11061606 37371701 PMC10295691

[200] Xu H Garcia-Ptacek S Trevisan M Evans M Lindholm B Eriksdotter M. Kidney function, kidney function decline, and the risk of dementia in older adults. Neurology. (2021) 96:e2956–e65. 10.1212/WNL.0000000000012113 33952656 PMC8253567

[201] Stocker H Beyer L Trares K Perna L Rujescu D Holleczek B. Association of kidney function with development of Alzheimer disease and other dementias and dementia-related blood biomarkers. JAMA Netw Open. (2023) 6:e2252387. 10.1001/jamanetworkopen.2022.52387 36692879 PMC10408272

[202] Freeman SH Kandel R Cruz L Rozkalne A Newell K Frosch MP. Preservation of neuronal number despite age-related cortical brain atrophy in elderly subjects without Alzheimer disease. J Neuropathol Exp Neurol. (2008) 67:1205–12. 10.1097/NEN.0b013e31818fc72f 19018241 PMC2734185

[203] JA Y. Rate of change of common measures of impairment in senile dementia of the Alzheimer type. Psychopharmacol Bull. (1988) 24:531–4. 3074314

[204] DeCarli C. Mild cognitive impairment: prevalence, prognosis, aetiology, and treatment. Lancet Neurol. (2003) 2:15–21. 10.1016/S1474-4422(03)00262-X 12849297

[205] Laske C Sohrabi HR Frost SM López-de-Ipiña K Garrard P Buscema M. Innovative diagnostic tools for early detection of Alzheimer's disease. Alzheimers Dement. (2015) 11:561–78. 10.1016/j.jalz.2014.06.004 25443858

[206] DeTure MA Dickson DW. The neuropathological diagnosis of Alzheimer's disease. Mol Neurodegener. (2019) 14:1–18. 10.1186/s13024-019-0333-5 31375134 PMC6679484

[207] Aggarwal NT DeCarli C editors. Vascular dementia: emerging trends. Semin Neurol. (2007) 27:66–77. 10.1055/s-2006-956757 17226743

[208] Grober E Hall CB Lipton RB Zonderman AB Resnick SM Kawas C. Memory impairment, executive dysfunction, and intellectual decline in preclinical Alzheimer's disease. J Int Neuropsychol Soc. (2008) 14:266–78. 10.1017/S1355617708080302 18282324 PMC2763488

[209] Tierney M Szalai J Snow W Fisher R Nores A Nadon G. Prediction of probable Alzheimer's disease in memory-impaired patients: a prospective longitudinal study. Neurology. (1996) 46:661–5. 10.1212/WNL.46.3.661 8618663

[210] Tarawneh R Holtzman DM. The clinical problem of symptomatic Alzheimer disease and mild cognitive impairment. Cold Spring Harb Perspect Med. (2012) 2:a006148. 10.1101/cshperspect.a006148 22553492 PMC3331682

[211] Emery VOB. Language impairment in dementia of the Alzheimer type: a hierarchical decline? Int J Psychiatry Med. (2000) 30:145–64. 10.2190/X09P-N7AU-UCHA-VW08 11001278

[212] Appell J Kertesz A Fisman M. A study of language functioning in Alzheimer patients. Brain Lang. (1982) 17:73–91. 10.1016/0093-934X(82)90006-2 7139272

[213] Goldstein FC Green J Presley R Green RC. Dysnomia in Alzheimer's disease: an evaluation of neurobehavioral subtypes. Brain Lang. (1992) 43:308–22. 10.1016/0093-934X(92)90132-X 1393524

[214] Montembeault M Migliaccio R. Atypical forms of Alzheimer's disease: patients not to forget. Curr Opin Neurol. (2023) 36:245–52. 10.1097/WCO.0000000000001182 37365819

[215] Carlomagno S Lavarone A Nolfe G Bourene G Martin C Deloche G. Dyscalculia in the early stages of Alzheimer's disease. Acta Neurol Scand. (1999) 99:166–74. 10.1111/j.1600-0404.1999.tb07339.x 10100960

[216] Delazer M Sinz H Zamarian L Benke T. Decision-making with explicit and stable rules in mild Alzheimer's disease. Neuropsychologia. (2007) 45:1632–41. 10.1016/j.neuropsychologia.2007.01.006 17328931

[217] High Jr WM Levin HS Gary Jr HE. Recovery of orientation following closed-head injury. J Clin Exp Neuropsychol. (1990) 12:703–14. 10.1080/01688639008401013 2258432

[218] Benton A Van Allen M Fogel M. Temporal orientation in cerebral disease. J Nerv Ment Dis. (1964) 139:110–9. 10.1097/00005053-196408000-00003 14206449

[219] Monacelli AM Cushman LA Kavcic V Duffy CJ. Spatial disorientation in Alzheimer's disease: the remembrance of things passed. Neurology. (2003) 61:1491–7. 10.1212/WNL.61.11.1491 14663030

[220] Thiyagesh SN Farrow TF Parks RW Accosta-Mesa H Young C Wilkinson ID. The neural basis of visuospatial perception in Alzheimer's disease and healthy elderly comparison subjects: an fMRI study. Psychiatry Res. (2009) 172:109–16. 10.1016/j.pscychresns.2008.11.002 19324533

[221] Crutch SJ Lehmann M Schott JM Rabinovici GD Rossor MN Fox NC. Posterior cortical atrophy. Lancet Neurol. (2012) 11:170–8. 10.1016/S1474-4422(11)70289-7 22265212 PMC3740271

[222] Förstl H Kurz A. Clinical features of Alzheimer's disease. Eur Arch Psychiatry Clin Neurosci. (1999) 249:288–90. 10.1007/s004060050101 10653284

[223] Engedal K Barca ML Laks J Selbaek G. Depression in Alzheimer's disease: specificity of depressive symptoms using three different clinical criteria. Int J Geriatr Psychiatry. (2011) 26:944–51. 10.1002/gps.2631 21845597

[224] Gras LZ Kanaan SF McDowd JM Colgrove YM Burns J Pohl PS. Balance and gait of adults with very mild Alzheimer's disease. J Geriatr Phys Ther. (2001) 38:1. 10.1519/JPT.0000000000000020 24755691 PMC4632639

[225] Mölsä PK Marttila RJ Rinne UK. Extrapyramidal signs in Alzheimer's disease. Neurology. (1984) 34:1114. 10.1212/WNL.34.8.1114 6540392

[226] Franssen EH Reisberg B. Neurologic markers of the progression of Alzheimer's disease. Int Psychogeriatr. (1997) 9:297–306. 10.1017/S1041610297005036 9447450

[227] Reisberg B Borenstein J Salob SP Ferris SH Franssen E Georgotas A. Behavioral symptoms in Alzheimer's disease: phenomenology and treatment. J Clin Psychiatry. (1987) 48:9–15. 10.1037/t13385-000 3553166

[228] Reisberg B Auer SR Monteiro IM. Behavioral pathology in Alzheimer's disease (BEHAVE-AD) rating scale. Int Psychogeriatr. (1997) 8:301–8. 10.1017/S1041610297003529 9154579

[229] Whitehead D Tunnard C Hurt C Wahlund L Mecocci P Tsolaki M. Frontotemporal atrophy associated with paranoid delusions in women with Alzheimer's disease. Int Psychogeriatr. (2012) 24:99–107. 10.1017/S1041610211000974 21740613

[230] Huff FJ Boller F Lucchelli F Querriera R Beyer J Belle S. The Neurologic examination in patients with probable Alzheimer's disease. Arch Neurol. (1987) 44:929–32. 10.1001/archneur.1987.00520210031015 3619712

[231] Nelson PT Alafuzoff I Bigio EH Bouras C Braak H Cairns NJ. Correlation of Alzheimer disease neuropathologic changes with cognitive status: a review of the literature. J Neuropathol Exp Neurol. (2012) 71:362–81. 10.1097/NEN.0b013e31825018f7 22487856 PMC3560290

[232] Jahn H. Memory loss in Alzheimer's disease. Dialog Clin Neurosci. (2013) 15:445–54. 10.31887/DCNS.2013.15.4/hjahn 24459411 PMC3898682

[233] Mortamais M Ash JA Harrison JE Kaye JA Kramer J Randolph C. Detecting cognitive changes in preclinical Alzheimer's disease: a review of its feasibility. Alzheimers Dement. (2017) 13:468–92. 10.1016/j.jalz.2016.06.2365 27702618

[234] Knopman DS Caselli RJ. Appraisal of cognition in preclinical Alzheimer's disease: a conceptual review. Neurodegener Dis Manag. (2012) 2:183–95. 10.2217/nmt.12.5 22798965 PMC3395065

[235] Salmon DP Bondi MW. Neuropsychological assessment of dementia. Annu Rev Psychol. (2009) 60:257–82. 10.1146/annurev.psych.57.102904.190024 18616392 PMC2864104

[236] Albert MS. Cognitive and neurobiologic markers of early Alzheimer disease. Proc Nat Acad Sci USA. (1996) 93:13547–51. 10.1073/pnas.93.24.13547 8942970 PMC33644

[237] Balsis S Carpenter BD Storandt M. Personality change precedes clinical diagnosis of dementia of the Alzheimer type. J Gerontol B Psychol Sci Soc Sci. (2005) 60:P98–101. 10.1093/geronb/60.2.P98 15746024

[238] Tautvydaite D Antonietti JP Henry H von Gunten A Popp J. Relations between personality changes and cerebrospinal fluid biomarkers of Alzheimer's disease pathology. J Psychiatr Res. (2017) 90:12–20. 10.1016/j.jpsychires.2016.12.024 28213293

[239] Robins Wahlin TB Byrne GJ. Personality changes in Alzheimer's disease: a systematic review. Int J Geriatr Psychiatry. (2011) 26:1019–29. 10.1002/gps.2655 21905097

[240] Pocnet C Rossier J Antonietti J-P von Gunten A. personality changes in patients with beginning Alzheimer disease. Can J Psychiatry. (2011) 56:408–17. 10.1177/070674371105600704 21835104

[241] Talassi E Cipriani G Bianchetti A Trabucchi M. Personality changes in Alzheimer's disease. Aging Ment Health. (2007) 11:526–31. 10.1080/13607860601086603 17882590

[242] Yoon B Choi SH Jeong JH Park KW Kim E-J Hwang J. Balance and mobility performance along the Alzheimer's disease spectrum. J Alzheimers Dis. (2020) 73:633–44. 10.3233/JAD-190601 31815691

[243] Rolland Y van Kan GA Nourhashemi F Andrieu S Cantet C Guyonnet-Gillette S. An abnormal “one-leg balance” test predicts cognitive decline during Alzheimer's disease. J Alzheimers Dis. (2009) 16:525–31. 10.3233/JAD-2009-0987 19276547

[244] Suttanon P Hill KD Said CM LoGiudice D Lautenschlager NT Dodd KJ. Balance and mobility dysfunction and falls risk in older people with mild to moderate Alzheimer disease. Am J Phys Med Rehabil. (2012) 91:12–23. 10.1097/PHM.0b013e31823caeea 22157433

[245] de Oliveira Silva F Ferreira JV Plácido J Chagas D Praxedes J Guimarães C. Gait analysis with videogrammetry can differentiate healthy elderly, mild cognitive impairment, and Alzheimer's disease: a cross-sectional study. Exp Gerontol. (2020) 131:110816. 10.1016/j.exger.2019.110816 31862421

[246] Valkanova V Ebmeier KP. What can gait tell us about dementia? Review of epidemiological and neuropsychological evidence. Gait Post. (2017) 53:215–23. 10.1016/j.gaitpost.2017.01.024 28222369

[247] Cedervall Y Halvorsen K Åberg AC. A longitudinal study of gait function and characteristics of gait disturbance in individuals with Alzheimer's disease. Gait Post. (2014) 39:1022–7. 10.1016/j.gaitpost.2013.12.026 24491520

[248] Jessen F Kucharski C Fries T Papassotiropoulos A Hoenig K Maier W. Sensory gating deficit expressed by a disturbed suppression of the P50 event-related potential in patients with Alzheimer's disease. Am J Psychiatry. (2001) 158:1319–21. 10.1176/appi.ajp.158.8.1319 11481170

[249] Kunz M Lautenbacher S. The impact of Alzheimer's disease on the pain processing. Fortschr Neurol Psychiatr. (2004) 7:375–82. 10.1055/s-2004-818389 15252751

[250] Fisher-Morris M Gellafly A. The experience and expression of pain in Alzheimer patients. Age Ageing. (1997) 26:497–500. 10.1093/ageing/26.6.497 9466303

[251] Gabelle A Gutierrez L-A Dartigues J-F Ritchie K Touchon J Berr C. Palmomental reflex a relevant sign in early Alzheimer's disease diagnosis? J Alzheimers Dis. (2016) 49:1135–41. 10.3233/JAD-150436 26639955 PMC4927824

[252] Vreeling FW Houx PJ Jolles J Verhey FRJ. Primitive reflexes in Alzheimer's disease and vascular dementia. J Geriatr Psychiatry Neurol. (1995) 8:111–7. 10.1177/089198879500800207 7794474

[253] Molitor RJ Ko PC Ally BA. Eye movements in Alzheimer's disease. J Alzheimers Dis. (2015) 44:1–12. 10.3233/JAD-141173 25182738 PMC5332166

[254] Nam U Lee K Ko H Lee J-Y Lee EC. Analyzing facial and eye movements to screen for Alzheimer's disease. Sensors. (2020) 20:5349. 10.3390/s20185349 32961984 PMC7570590

[255] Hart NJ Koronyo Y Black KL Koronyo-Hamaoui M. Ocular indicators of Alzheimer's: exploring disease in the retina. Acta Neuropathol. (2016) 132:767–87. 10.1007/s00401-016-1613-6 27645291 PMC5106496

[256] Armstrong R Kergoat H. Oculo-visual changes and clinical considerations affecting older patients with dementia. Ophthal Physiol Optics. (2015) 35:352–76. 10.1111/opo.12220 26094831

[257] Chang LYL Lowe J Ardiles A Lim J Grey AC Robertson K. Alzheimer's disease in the human eye. Clinical tests that identify ocular and visual information processing deficit as biomarkers. Alzheimers Dement. (2014) 10:251–61. 10.1016/j.jalz.2013.06.004 24011928

[258] Javaid FZ Brenton J Guo L Cordeiro MF. Visual and ocular manifestations of Alzheimer's disease and their use as biomarkers for diagnosis and progression. Front Neurol. (2016) 7:55. 10.3389/fneur.2016.00055 27148157 PMC4836138

[259] Nolan JM Loskutova E Howard AN Moran R Mulcahy R Stack J. Macular pigment, visual function, and macular disease among subjects with Alzheimer's disease: an exploratory study. J Alzheimers Dis. (2014) 42:1191–202. 10.3233/JAD-140507 25024317

[260] Tzekov R Mullan M. Vision function abnormalities in Alzheimer disease. Surv Ophthalmol. (2014) 59:414–33. 10.1016/j.survophthal.2013.10.002 24309127

[261] den Haan J Verbraak FD Visser PJ Bouwman FH. Retinal thickness in Alzheimer's disease: a systematic review and meta-analysis. Alzheimers Dement. (2017) 6:162–70. 10.1016/j.dadm.2016.12.014 28275698 PMC5328759

[262] Soininen H Laulumaa V Helkala EL Hartikainen P Riekkinen PJ. Extrapyramidal signs in Alzheimer's disease: a 3-year follow-up study. J Neural Transm Park Dis Dement Sect. (1992) 4:107–19. 10.1007/BF02251474 1349210

[263] Lopez OL Wisnieski SR Becker JT Boller F DeKosky ST. Extrapyramidal signs in patients with probable Alzheimer disease. Arch Neurol. (1997) 54:969–75. 10.1001/archneur.1997.00550200033007 9267971

[264] Chen JY Stern Y Sano M Mayeux R. Cumulative risks of developing extrapyramidal signs, psychosis, or myoclonus in the course of Alzheimer's disease. Arch Neurol. (1991) 48:1141–3. 10.1001/archneur.1991.00530230049020 1953398

[265] Ellis RJ Caligiuri M Galasko D Thal LJ. Extrapyramidal motor signs in clinically diagnosed Alzheimer disease. Alzheimer Dis Assoc Disord. (1996) 10:103–14. 10.1097/00002093-199601020-00008 8727172

[266] Scarmeas N Hadjigeorgiou GM Papadimitriou A Dubois B Sarazin M Brandt J. Motor signs during the course of Alzheimer disease. Neurology. (2004) 63:975. 10.1212/01.WNL.0000138440.39918.0C 15452286 PMC3028531

[267] Wilson RS Bennett DA Gilley DW Beckett LA Schneider JA Evans DA. Progression of parkinsonian signs in Alzheimer's disease. Neurology. (2000) 54:1284–9. 10.1212/WNL.54.6.1284 10746599

[268] Minati L Edginton T Grazia Bruzzone M Giaccone G. Reviews: current concepts in Alzheimer's disease: a multidisciplinary review. Am J Alzheimers Dis Other Dement. (2009) 24:95–121. 10.1177/1533317508328602 19116299 PMC10846154

[269] Meijs AP Claassen JAHR Olde Rikkert MGM Schalk BWM Meulenbroek O Kessels RPC. How does additional diagnostic testing influence the initial diagnosis in patients with cognitive complaints in a memory clinic setting? Age Ageing. (2015) 44:72–7. 10.1093/ageing/afu053 24847028

[270] Albert MS DeKosky ST Dickson D Dubois B Feldman HH Fox NC. The diagnosis of mild cognitive impairment due to Alzheimer's disease: recommendations from the National Institute on Aging-Alzheimer's Association workgroups on diagnostic guidelines for Alzheimer's disease. Alzheimers Dement. (2011) 7:270–9. 10.1016/j.jalz.2011.03.008 21514249 PMC3312027

[271] Dickerson B Wolk D. Biomarker-based prediction of progression in MCI: comparison of AD-signature and hippocampal volume with spinal fluid amyloid-β and tau. Front Aging Neurosci. (2013) 5:55. 10.3389/fnagi.2013.00055 24130528 PMC3795312

[272] Steffen W Frank J Alexander K Luca K Klaus S Lutz F. Subjective cognitive decline is related to CSF biomarkers of AD in patients with MCI. Neurology. (2015) 84:1261. 10.1212/WNL.0000000000001399 25716354

[273] Kiddle SJ Voyle N Dobson RJB. A blood test for Alzheimer's disease: progress, challenges, and recommendations. J Alzheimers Dis. (2018) 64:S289–s97. 10.3233/JAD-179904 29614671 PMC6010156

[274] Nakamura A Kaneko N Villemagne VL Kato T Doecke J Doré V. High performance plasma amyloid-β biomarkers for Alzheimer's disease. Nature. (2018) 554:249–54. 10.1038/nature25456 29420472

[275] Ovod V Ramsey KN Mawuenyega KG Bollinger JG Hicks T Schneider T. Amyloid β concentrations and stable isotope labeling kinetics of human plasma specific to central nervous system amyloidosis. Alzheimers Dement. (2017) 13:841–9. 10.1016/j.jalz.2017.06.2266 28734653 PMC5567785

[276] Janelidze S Mattsson N Palmqvist S Smith R Beach TG Serrano GE. Plasma P-tau181 in Alzheimer's disease: relationship to other biomarkers, differential diagnosis, neuropathology and longitudinal progression to Alzheimer's dementia. Nat Med. (2020) 26:379–86. 10.1038/s41591-020-0755-1 32123385

[277] Thijssen EH La Joie R Wolf A Strom A Wang P Iaccarino L. Diagnostic value of plasma phosphorylated tau181 in Alzheimer's disease and frontotemporal lobar degeneration. Nat Med. (2020) 26:387–97. 10.1038/s41591-020-0762-2 32123386 PMC7101073

[278] Menzies FM Fleming A Rubinsztein DC. Compromised autophagy and neurodegenerative diseases. Nat Rev Neurosci. (2015) 16:345–57. 10.1038/nrn3961 25991442

[279] Swardfager W Lanctôt K Rothenburg L Wong A Cappell J Herrmann N. meta-analysis of cytokines in Alzheimer's disease. Biol Psychiatry. (2010) 68:930–41. 10.1016/j.biopsych.2010.06.012 20692646

[280] Bronzuoli MR Iacomino A Steardo L Scuderi C. Targeting neuroinflammation in Alzheimer's disease. J Inflamm Res. (2016) 9:199–208. 10.2147/JIR.S86958 27843334 PMC5098782

[281] Janelidze S Mattsson N Stomrud E Lindberg O Palmqvist S Zetterberg H. CSF biomarkers of neuroinflammation and cerebrovascular dysfunction in early Alzheimer disease. Neurology. (2018) 91:e867–e77. 10.1212/WNL.0000000000006082 30054439 PMC6133624

[282] Querol-Vilaseca M Colom-Cadena M Pegueroles J San Martín-Paniello C Clarimon J Belbin O. YKL-40 (Chitinase 3-like I) is expressed in a subset of astrocytes in Alzheimer's disease and other tauopathies. J Neuroinflamm. (2017) 14:118. 10.1186/s12974-017-0893-7 28599675 PMC5466718

[283] Prins S de Kam ML Teunissen CE Groeneveld GJ. Inflammatory plasma biomarkers in subjects with preclinical Alzheimer's disease. Alzheimers Res Ther. (2022) 14:106. 10.1186/s13195-022-01051-2 35922871 PMC9347121

[284] Seubert P Vigo-Pelfrey C Esch F Lee M Dovey H Davis D. Isolation and quantification of soluble Alzheimer's beta-peptide from biological fluids. Nature. (1992) 359:325–7. 10.1038/359325a0 1406936

[285] Olsson B Lautner R Andreasson U Öhrfelt A Portelius E Bjerke M. CSF and blood biomarkers for the diagnosis of Alzheimer's disease: a systematic review and meta-analysis. Lancet Neurol. (2016) 15:673–84. 10.1016/S1474-4422(16)00070-3 27068280

[286] Strozyk D Blennow K White LR Launer LJ CSF. Abeta 42 levels correlate with amyloid-neuropathology in a population-based autopsy study. Neurology. (2003) 60:652–6. 10.1212/01.WNL.0000046581.81650.D0 12601108

[287] Andreasen N Hesse C Davidsson P Minthon L Wallin A Winblad B. Cerebrospinal fluid beta-amyloid(1-42) in Alzheimer disease: differences between early- and late-onset Alzheimer disease and stability during the course of disease. Arch Neurol. (1999) 56:673–80. 10.1001/archneur.56.6.673 10369305

[288] Portelius E Tran AJ Andreasson U Persson R Brinkmalm G Zetterberg H. Characterization of amyloid beta peptides in cerebrospinal fluid by an automated immunoprecipitation procedure followed by mass spectrometry. J Proteome Res. (2007) 6:4433–9. 10.1021/pr0703627 17927230

[289] Portelius E Westman-Brinkmalm A Zetterberg H Blennow K. Determination of beta-amyloid peptide signatures in cerebrospinal fluid using immunoprecipitation-mass spectrometry. J Proteome Res. (2006) 5:1010–6. 10.1021/pr050475v 16602710

[290] Hansson O Zetterberg H Buchhave P Andreasson U Londos E Minthon L. Prediction of Alzheimer's disease using the CSF Abeta42/Abeta40 ratio in patients with mild cognitive impairment. Dement Geriatr Cogn Disord. (2007) 23:316–20. 10.1159/000100926 17374949

[291] Lewczuk P Esselmann H Otto M Maler JM Henkel AW Henkel MK. Neurochemical diagnosis of Alzheimer's dementia by CSF Abeta42, Abeta42/Abeta40 ratio and total tau. Neurobiol Aging. (2004) 25:273–81. 10.1016/S0197-4580(03)00086-1 15123331

[292] Wiltfang J Esselmann H Bibl M Hüll M Hampel H Kessler H. Amyloid beta peptide ratio 42/40 but not A beta 42 correlates with phospho-Tau in patients with low- and high-CSF A beta 40 load. J Neurochem. (2007) 101:1053–9. 10.1111/j.1471-4159.2006.04404.x 17254013

[293] Dumurgier J Schraen S Gabelle A Vercruysse O Bombois S Laplanche JL. Cerebrospinal fluid amyloid-β 42/40 ratio in clinical setting of memory centers: a multicentric study. Alzheimers Res Ther. (2015) 7:30. 10.1186/s13195-015-0114-5 26034513 PMC4450486

[294] Barten DM Cadelina GW Hoque N DeCarr LB Guss VL Yang L. Tau transgenic mice as models for cerebrospinal fluid tau biomarkers. J Alzheimers Dis. (2011) 24 Suppl 2:127–41. 10.3233/JAD-2011-110161 21422517

[295] Khan SS Bloom GS. Tau: the center of a signaling nexus in Alzheimer's disease. Front Neurosci. (2016) 10:31. 10.3389/fnins.2016.00031 26903798 PMC4746348

[296] Hampel H Blennow K Shaw LM Hoessler YC Zetterberg H Trojanowski JQ. Total and phosphorylated tau protein as biological markers of Alzheimer's disease. Exp Gerontol. (2010) 45:30–40. 10.1016/j.exger.2009.10.010 19853650 PMC2815003

[297] Blennow K Hampel H. CSF markers for incipient Alzheimer's disease. Lancet Neurol. (2003) 2:605–13. 10.1016/S1474-4422(03)00530-1 14505582

[298] Zetterberg H Hietala MA Jonsson M Andreasen N Styrud E Karlsson I. Neurochemical aftermath of amateur boxing. Arch Neurol. (2006) 63:1277–80. 10.1001/archneur.63.9.1277 16966505

[299] Hesse C Rosengren L Andreasen N Davidsson P Vanderstichele H Vanmechelen E. Transient increase in total tau but not phospho-tau in human cerebrospinal fluid after acute stroke. Neurosci Lett. (2001) 297:187–90. 10.1016/S0304-3940(00)01697-9 11137759

[300] Grossman M Farmer J Leight S Work M Moore P Van Deerlin V. Cerebrospinal fluid profile in frontotemporal dementia and Alzheimer's disease. Ann Neurol. (2005) 57:721–9. 10.1002/ana.20477 15852395

[301] Bian H Van Swieten JC Leight S Massimo L Wood E Forman M. CSF biomarkers in frontotemporal lobar degeneration with known pathology. Neurology. (2008) 70:1827–35. 10.1212/01.wnl.0000311445.21321.fc 18458217 PMC2707002

[302] Vanmechelen E Vanderstichele H Davidsson P Van Kerschaver E Van Der Perre B Sjögren M. Quantification of tau phosphorylated at threonine 181 in human cerebrospinal fluid: a sandwich ELISA with a synthetic phosphopeptide for standardization. Neurosci Lett. (2000) 285:49–52. 10.1016/S0304-3940(00)01036-3 10788705

[303] Hampel H Teipel SJ. Total and phosphorylated tau proteins: evaluation as core biomarker candidates in frontotemporal dementia. Dement Geriatr Cogn Disord. (2004) 17:350–4. 10.1159/000077170 15178952

[304] Hampel H Buerger K Zinkowski R Teipel SJ Goernitz A Andreasen N. Measurement of phosphorylated tau epitopes in the differential diagnosis of Alzheimer disease: a comparative cerebrospinal fluid study. Arch Gen Psychiatry. (2004) 61:95–102. 10.1001/archpsyc.61.1.95 14706948

[305] Buerger K Zinkowski R Teipel SJ Arai H DeBernardis J Kerkman D. Differentiation of geriatric major depression from Alzheimer's disease with CSF tau protein phosphorylated at threonine 231. Am J Psychiatry. (2003) 160:376–9. 10.1176/appi.ajp.160.2.376 12562590

[306] Barthélemy NR Li Y Joseph-Mathurin N Gordon BA Hassenstab J Benzinger TLS. A soluble phosphorylated tau signature links tau, amyloid and the evolution of stages of dominantly inherited Alzheimer's disease. Nat Med. (2020) 26:398–407. 10.1038/s41591-020-0781-z 32161412 PMC7309367

[307] Buerger K Teipel SJ Zinkowski R Blennow K Arai H Engel R. CSF tau protein phosphorylated at threonine 231 correlates with cognitive decline in MCI subjects. Neurology. (2002) 59:627–9. 10.1212/WNL.59.4.627 12196665

[308] Buerger K Ewers M Pirttilä T Zinkowski R Alafuzoff I Teipel SJ. CSF phosphorylated tau protein correlates with neocortical neurofibrillary pathology in Alzheimer's disease. Brain. (2006) 129(Pt 11):3035–41. 10.1093/brain/awl269 17012293

[309] Blom ES Giedraitis V Zetterberg H Fukumoto H Blennow K Hyman BT. Rapid progression from mild cognitive impairment to Alzheimer's disease in subjects with elevated levels of tau in cerebrospinal fluid and the APOE epsilon4/epsilon4 genotype. Dement Geriatr Cogn Disord. (2009) 27:458–64. 10.1159/000216841 19420940 PMC7077080

[310] Buerger K Ewers M Andreasen N Zinkowski R Ishiguro K Vanmechelen E. Phosphorylated tau predicts rate of cognitive decline in MCI subjects: a comparative CSF study. Neurology. (2005) 65:1502–3. 10.1212/01.wnl.0000183284.92920.f2 16275849

[311] Sämgård K Zetterberg H Blennow K Hansson O Minthon L Londos E. Cerebrospinal fluid total tau as a marker of Alzheimer's disease intensity. Int J Geriatr Psychiatry. (2010) 25:403–10. 10.1002/gps.2353 19650161

[312] Hampel H Buerger K Kohnken R Teipel SJ Zinkowski R Moeller HJ. Tracking of Alzheimer's disease progression with cerebrospinal fluid tau protein phosphorylated at threonine 231. Ann Neurol. (2001) 49:545–6. 10.1002/ana.111.abs 11310639

[313] Hampel H Bürger K Pruessner JC Zinkowski R DeBernardis J Kerkman D. Correlation of cerebrospinal fluid levels of tau protein phosphorylated at threonine 231 with rates of hippocampal atrophy in Alzheimer disease. Arch Neurol. (2005) 62:770–3. 10.1001/archneur.62.5.770 15883264

[314] Ewers M Buerger K Teipel SJ Scheltens P Schröder J Zinkowski RP. Multicenter assessment of CSF-phosphorylated tau for the prediction of conversion of MCI. Neurology. (2007) 69:2205–12. 10.1212/01.wnl.0000286944.22262.ff 18071141

[315] Yuan A Rao MV Veeranna Nixon RA. Neurofilaments at a glance. J Cell Sci. (2012) 125(Pt 14):3257-63. 10.1242/jcs.104729 22956720 PMC3516374

[316] Yuan A Rao MV Veeranna Nixon RA. Neurofilaments and neurofilament proteins in health and disease. Cold Spring Harb Perspect Biol. (2017) 9:a018309. 10.1101/cshperspect.a018309 28373358 PMC5378049

[317] Vickers JC Riederer BM Marugg RA Buée-Scherrer V Buée L Delacourte A. Alterations in neurofilament protein immunoreactivity in human hippocampal neurons related to normal aging and Alzheimer's disease. Neuroscience. (1994) 62:1–13. 10.1016/0306-4522(94)90310-7 7816192

[318] Lycke JN Karlsson JE Andersen O Rosengren LE. Neurofilament protein in cerebrospinal fluid: a potential marker of activity in multiple sclerosis. J Neurol Neurosurg Psychiatry. (1998) 64:402–4. 10.1136/jnnp.64.3.402 9527161 PMC2170011

[319] Petzold A Keir G Warren J Fox N Rossor MN. A systematic review and meta-analysis of CSF neurofilament protein levels as biomarkers in dementia. Neurodegener Dis. (2007) 4:185–94. 10.1159/000101843 17596713

[320] Sjögren M Blomberg M Jonsson M Wahlund LO Edman A Lind K. Neurofilament protein in cerebrospinal fluid: a marker of white matter changes. J Neurosci Res. (2001) 66:510–6. 10.1002/jnr.1242 11746370

[321] Alcolea D Vilaplana E Suárez-Calvet M Illán-Gala I Blesa R Clarimón J. CSF sAPPβ, YKL-40, and neurofilament light in frontotemporal lobar degeneration. Neurology. (2017) 89:178–88. 10.1212/WNL.0000000000004088 28592456

[322] Lista S Toschi N Baldacci F Zetterberg H Blennow K Kilimann I. Diagnostic accuracy of CSF neurofilament light chain protein in the biomarker-guided classification system for Alzheimer's disease. Neurochem Int. (2017) 108:355–60. 10.1016/j.neuint.2017.05.010 28527630

[323] Zetterberg H Skillbäck T Mattsson N Trojanowski JQ Portelius E Shaw LM. Association of cerebrospinal fluid neurofilament light concentration with Alzheimer disease progression. JAMA Neurol. (2016) 73:60–7. 10.1001/jamaneurol.2015.3037 26524180 PMC5624219

[324] Pereira JB Westman E Hansson O. Association between cerebrospinal fluid and plasma neurodegeneration biomarkers with brain atrophy in Alzheimer's disease. Neurobiol Aging. (2017) 58:14–29. 10.1016/j.neurobiolaging.2017.06.002 28692877

[325] Bergman J Dring A Zetterberg H Blennow K Norgren N Gilthorpe J. Neurofilament light in CSF and serum is a sensitive marker for axonal white matter injury in MS. Neurol Neuroimmunol Neuroinflamm. (2016) 3:e271. 10.1212/NXI.0000000000000271 27536708 PMC4972001

[326] Gendron TF Daughrity LM Heckman MG Diehl NN Wuu J Miller TM. Phosphorylated neurofilament heavy chain: a biomarker of survival for C9ORF72-associated amyotrophic lateral sclerosis. Ann Neurol. (2017) 82:139–46. 10.1002/ana.24980 28628244 PMC5676468

[327] Femminella GD Edison P. Evaluation of neuroprotective effect of glucagon-like peptide 1 analogs using neuroimaging. Alzheimers Dement. (2014) 10:S55–61. 10.1016/j.jalz.2013.12.012 24529526

[328] Jack CR Jr Holtzman DM. Biomarker modeling of Alzheimer's disease. Neuron. (2013) 80:1347–58. 10.1016/j.neuron.2013.12.003 24360540 PMC3928967

[329] Danborg PB Simonsen AH Waldemar G Heegaard NH. The potential of microRNAs as biofluid markers of neurodegenerative diseases–a systematic review. Biomarkers. (2014) 19:259–68. 10.3109/1354750X.2014.904001 24678935

[330] Alexandrov PN Dua P Hill JM Bhattacharjee S Zhao Y Lukiw WJ. microRNA (miRNA) speciation in Alzheimer's disease (AD) cerebrospinal fluid (CSF) and extracellular fluid (ECF). Int J Biochem Mol Biol. (2012) 3:365–73. 23301201 PMC3533883

[331] Chan HN Xu D Ho SL Wong MS Li HW. Ultra-sensitive detection of protein biomarkers for diagnosis of Alzheimer's disease. Chem Sci. (2017) 8:4012–8. 10.1039/C6SC05615F 30155210 PMC6094176

[332] Park JS Kim ST Kim SY Jo MG Choi MJ Kim MO. novel kit for early diagnosis of Alzheimer's disease using a fluorescent nanoparticle imaging. Sci Rep. (2019) 9:13184. 10.1038/s41598-019-49711-y 31515517 PMC6742761

[333] Ng A Tam WW Zhang MW Ho CS Husain SF McIntyre RS. IL-1β, IL-6, TNF- α and CRP in elderly patients with depression or Alzheimer's disease: systematic review and meta-analysis. Sci Rep. (2018) 8:12050. 10.1038/s41598-018-30487-6 30104698 PMC6089986

[334] Rasmussen J Langerman H. Alzheimer's disease - why we need early diagnosis. Degener Neurol Neuromuscul Dis. (2019) 9:123–30. 10.2147/DNND.S228939 31920420 PMC6935598

[335] PorsteinssonAPIsaacsonRSKnoxSSabbaghMNRubinoI. Diagnosis of early Alzheimer's disease: clinical practice in 2021. J Prev Alzheimers Dis. (2021) 8:371–86. 10.14283/jpad.2021.2334101796

[336] Johnson KA Fox NC Sperling RA Klunk WE. Brain imaging in Alzheimer disease. Cold Spring Harb Perspect Med. (2012) 2:a006213. 10.1101/cshperspect.a006213 22474610 PMC3312396

[337] Wu C-L Lin T-J Chiou G-L Lee C-Y Luan H Tsai M-J. A systematic review of MRI neuroimaging for education research. Front Psychol. (2021) 12:617599. 10.3389/fpsyg.2021.617599 34093308 PMC8174785

[338] Butler E Mounsey A. Structural mri for the early diagnosis of Alzheimer disease in patients with MCI. Am Fam Phys. (2021) 103:273–4. 10.1002/14651858.CD009628.pub2 33630555

[339] Scheltens P. Imaging in Alzheimer's disease. Dialogues Clin Neurosci. (2009) 11:191–9. 10.31887/DCNS.2009.11.2/pscheltens 19585954 PMC3181915

[340] Braak H Braak E. Morphological criteria for the recognition of Alzheimer's disease and the distribution pattern of cortical changes related to this disorder. Neurobiol Aging. (1994) 15:355–6. 10.1016/0197-4580(94)90032-9 7936061

[341] Scheltens P Fox N Barkhof F De Carli C. Structural magnetic resonance imaging in the practical assessment of dementia: beyond exclusion. Lancet Neurol. (2002) 1:13–21. 10.1016/S1474-4422(02)00002-9 12849541

[342] Chételat G Arbizu J Barthel H Garibotto V Law I Morbelli S. Amyloid-PET and 18F-FDG-PET in the diagnostic investigation of Alzheimer's disease and other dementias. Lancet Neurol. (2020) 19:951–62. 10.1016/S1474-4422(20)30314-8 33098804

[343] Mu Y Chang KX Chen YF Yan K Wang CX Hua Q. Diagnosis of Alzheimer's disease: Towards accuracy and accessibility. J Biol Methods. (2024) 11:e99010010. 10.14440/jbm.2024.412 38988499 PMC11231050

[344] Zhang M Ganz AB Hulsman M Bank NB Rozemuller AJM Scheltens P. Neuropathological hallmarks of Alzheimer's disease in centenarians, in the context of aging. Alzheimers Dement. (2021) 17:e053600. 10.1002/alz.053600

[345] Pascoal TA Shin M Kang MS Chamoun M Chartrand D Mathotaarachchi S. *In vivo* quantification of neurofibrillary tangles with [18F]MK-6240. Alzheimers Res Ther. (2018) 10:74. 10.1186/s13195-018-0402-y 30064520 PMC6069775

[346] Mantel E Williams J. An introduction to newer PET diagnostic agents and related therapeutic radiopharmaceuticals. J Nucl Med Technol. (2019) 47:203–9. 10.2967/jnmt.118.224022 31019034

[347] Maschio C Ni R. Amyloid and tau positron emission tomography imaging in Alzheimer's disease and other tauopathies. Front Aging Neurosci. (2022) 14:838034. 10.3389/fnagi.2022.838034 35527737 PMC9074832

[348] Leuzy A Chiotis K Lemoine L Gillberg PG Almkvist O Rodriguez-Vieitez E. Tau PET imaging in neurodegenerative tauopathies-still a challenge. Mol Psychiatry. (2019) 24:1112–34. 10.1038/s41380-018-0342-8 30635637 PMC6756230

[349] Burkett BJ Johnson DR Lowe VJ. Evaluation of neurodegenerative disorders with amyloid-β, tau, and dopaminergic PET imaging: interpretation pitfalls. J Nucl Med. (2024) 65:829–37. 10.2967/jnumed.123.266463 38664015 PMC13671385

[350] Tanner JA Rabinovici GD. Relationship between tau and cognition in the evolution of Alzheimer's disease: new insights from tau PET. J Nucl Med. (2021) 62:612–3. 10.2967/jnumed.120.257824 33277390 PMC9364866

[351] Marcus C Mena E Subramaniam RM. Brain PET in the diagnosis of Alzheimer's disease. Clin Nucl Med. (2014) 39:e413. 10.1097/RLU.0000000000000547 25199063 PMC4332800

[352] Mosconi L Mistur R Switalski R Tsui WH Glodzik L Li Y. FDG-PET changes in brain glucose metabolism from normal cognition to pathologically verified Alzheimer's disease. Eur J Nucl Med Mol Imaging. (2009) 36:811–22. 10.1007/s00259-008-1039-z 19142633 PMC2774795

[353] Bouter C Henniges P Franke TN Irwin C Sahlmann CO Sichler ME. 18F-FDG-PET detects drastic changes in brain metabolism in the Tg4–42 model of Alzheimer's disease. Front Aging Neurosci. (2019) 10:425. 10.3389/fnagi.2018.00425 30670962 PMC6333025

[354] Rabinovici G Rosen H Alkalay A Kornak J Furst A Agarwal N. Amyloid vs FDG-PET in the differential diagnosis of AD and FTLD. Neurology. (2011) 77:2034–42. 10.1212/WNL.0b013e31823b9c5e 22131541 PMC3236517

[355] Ou Y-N Xu W Li J-Q Guo Y Cui M Chen K-L. FDG-PET as an independent biomarker for Alzheimer's biological diagnosis: a longitudinal study. Alzheimers Res Therapy. (2019) 11:1–11. 10.1186/s13195-019-0512-1 31253185 PMC6599313

[356] Fink HA Linskens EJ Silverman PC McCarten JR Hemmy LS Ouellette JM. Accuracy of biomarker testing for neuropathologically defined Alzheimer disease in older adults with dementia: a systematic review. Ann Intern Med. (2020) 172:669–77. 10.7326/M19-3888 32340038

[357] Pope C Karanth S Liu J. Pharmacology and toxicology of cholinesterase inhibitors: uses and misuses of a common mechanism of action. Environ Toxicol Pharmacol. (2005) 19:433–46. 10.1016/j.etap.2004.12.048 21783509

[358] Håkansson L. Mechanism of action of cholinesterase inhibitors in Alzheimer's disease. Acta Neurol Scand. (1993) 88:7–9. 10.1111/j.1600-0404.1993.tb04245.x 7907455

[359] Wang H Zong Y Han Y Zhao J Liu H Liu Y. Compared of efficacy and safety of high-dose donepezil vs standard-dose donepezil among elderly patients with Alzheimer's disease: a systematic review and meta-analysis. Expert Opin Drug Saf. (2022) 21:407–15. 10.1080/14740338.2022.2027905 35099343

[360] Zhang X Lian S Zhang Y Zhao Q. Efficacy and safety of donepezil for mild cognitive impairment: a systematic review and meta-analysis. Clin Neurol Neurosurg. (2022) 213:107134. 10.1016/j.clineuro.2022.107134 35078087

[361] Yoshida K Seo M Luo Y Sahker E Cipriani A Leucht S. Personalized prediction of Alzheimer's disease and its treatment effects by donepezil: an individual participant data meta-analysis of eight randomized controlled trials. J Alzheimers Dis. (2022) 89:1143–57. 10.3233/JAD-220263 35988219

[362] Birks JS Chong LY Grimley Evans J. Rivastigmine for Alzheimer's disease. Cochrane Database Syst Rev. (2015) 9:Cd001191. 10.1002/14651858.CD001191.pub4 26393402 PMC7050299

[363] Henderson EJ Lord SR Brodie MA Gaunt DM Lawrence AD Close JC. Rivastigmine for gait stability in patients with Parkinson's disease (ReSPonD): a randomised, double-blind, placebo-controlled, phase 2 trial. Lancet Neurol. (2016) 15:249–58. 10.1016/S1474-4422(15)00389-0 26795874

[364] Birks JS Grimley Evans J. Rivastigmine for Alzheimer's disease. Cochrane Database Syst Rev. (2015) Cd001191. 10.1002/14651858.CD001191.pub3 25858345

[365] Lilienfeld S. Galantamine–a novel cholinergic drug with a unique dual mode of action for the treatment of patients with Alzheimer's disease. CNS Drug Rev. (2002) 8:159–76. 10.1111/j.1527-3458.2002.tb00221.x 12177686 PMC6741688

[366] Olazarán J García G. Galantamine: a novel cholinergic agent for Alzheimer's disease. Neurologia. (2002) 17:429–36. 12396973

[367] Corey-Bloom J. Galantamine: a review of its use in Alzheimer's disease and vascular dementia. Int J Clin Pract. (2003) 57:219–23. 10.1111/j.1742-1241.2003.tb10467.x 12723727

[368] Jiang D Yang X Li M Wang Y Wang Y. Efficacy and safety of galantamine treatment for patients with Alzheimer's disease: a meta-analysis of randomized controlled trials. J Neural Transm. (2015) 122:1157–66. 10.1007/s00702-014-1358-0 25547862

[369] Crismon ML. Tacrine: first drug approved for Alzheimer's disease. Ann Pharmacother. (1994) 28:744–51. 10.1177/106002809402800612 7919566

[370] Horak M Holubova K Nepovimova E Krusek J Kaniakova M Korabecny J. The pharmacology of tacrine at N-methyl-d-aspartate receptors. Prog Neuropsychopharmacol Biol Psychiatry. (2017) 75:54–62. 10.1016/j.pnpbp.2017.01.003 28089695

[371] Qizilbash N Whitehead A Higgins J Wilcock G Schneider L Farlow M. Cholinesterase inhibition for Alzheimer disease: a meta-analysis of the tacrine trials. Dementia Trialists' Collaboration. JAMA. (1998) 280:1777–82. 10.1001/jama.280.20.1777 9842955

[372] Watkins PB Zimmerman HJ Knapp MJ Gracon SI Lewis KW. Hepatotoxic effects of tacrine administration in patients with Alzheimer's disease. JAMA. (1994) 271:992–8. 10.1001/jama.271.13.992 8139084

[373] Ortiz-Sanz C Balantzategi U Quintela-López T Ruiz A Luchena C Zuazo-Ibarra J. Amyloid β / PKC-dependent alterations in NMDA receptor composition are detected in early stages of Alzheimer's disease. Cell Death Dis. (2022) 13:253. 10.1038/s41419-022-04687-y 35306512 PMC8934345

[374] Zhong W Wu A Berglund K Gu X Jiang MQ Talati J. Pathogenesis of sporadic Alzheimer's disease by deficiency of NMDA receptor subunit GluN3A. Alzheimers Dement. (2022) 18:222–39. 10.1002/alz.12398 34151525 PMC8685302

[375] Zhang Y Li P Feng J Wu M. Dysfunction of NMDA receptors in Alzheimer's disease. Neurol Sci. (2016) 37:1039–47. 10.1007/s10072-016-2546-5 26971324 PMC4917574

[376] Lipton SA. Paradigm shift in NMDA receptor antagonist drug development: molecular mechanism of uncompetitive inhibition by memantine in the treatment of Alzheimer's disease and other neurologic disorders. J Alzheimers Dis. (2004) 6:S61–74. 10.3233/JAD-2004-6S610 15665416

[377] Olivares D Deshpande VK Shi Y Lahiri DK Greig NH Rogers JT. N-methyl D-aspartate (NMDA) receptor antagonists and memantine treatment for Alzheimer's disease, vascular dementia and Parkinson's disease. Curr Alzheimer Res. (2012) 9:746–58. 10.2174/156720512801322564 21875407 PMC5002349

[378] Schmidt R Hofer E Bouwman FH Buerger K Cordonnier C Fladby T. EFNS-ENS/EAN Guideline on concomitant use of cholinesterase inhibitors and memantine in moderate to severe Alzheimer's disease. Eur J Neurol. (2015) 22:889–98. 10.1111/ene.12707 25808982

[379] Parsons CG Danysz W Dekundy A Pulte I. Memantine and cholinesterase inhibitors: complementary mechanisms in the treatment of Alzheimer's disease. Neurotox Res. (2013) 24:358–69. 10.1007/s12640-013-9398-z 23657927 PMC3753463

[380] Okuizumi K Kamata T Matsui D Saito K Matsumoto T Fukuchi Y. Memantine in Japanese patients with moderate to severe Alzheimer's disease: meta-analysis of multiple-index responder analyses. Expert Opin Pharmacother. (2018) 19:425–30. 10.1080/14656566.2018.1442440 29448852

[381] Hsu TW Chu CS Ching PY Chen GW Pan CC. The efficacy and tolerability of memantine for depressive symptoms in major mental diseases: a systematic review and updated meta-analysis of double-blind randomized controlled trials. J Affect Disord. (2022) 306:182–9. 10.1016/j.jad.2022.03.047 35331821

[382] McShane R Westby MJ Roberts E Minakaran N Schneider L Farrimond LE. Memantine for dementia. Cochr Database Syst Rev. (2019) 3:Cd003154. 10.1002/14651858.CD003154.pub6 30891742 PMC6425228

[383] Chen R Chan PT Chu H Lin YC Chang PC Chen CY. Treatment effects between monotherapy of donepezil versus combination with memantine for Alzheimer disease: a meta-analysis. PLoS ONE. (2017) 12:e0183586. 10.1371/journal.pone.0183586 28827830 PMC5565113

[384] Dou K-X Tan M-S Tan C-C Cao X-P Hou X-H Guo Q-H. Comparative safety and effectiveness of cholinesterase inhibitors and memantine for Alzheimer's disease: a network meta-analysis of 41 randomized controlled trials. Alzheimers Res Ther. (2018) 10:126. 10.1186/s13195-018-0457-9 30591071 PMC6309083

[385] Kennedy RE Cutter GR Fowler ME Schneider LS. Association of concomitant use of cholinesterase inhibitors or memantine with cognitive decline in Alzheimer clinical trials: a meta-analysis. JAMA Netw Open. (2018) 1:e184080. 10.1001/jamanetworkopen.2018.4080 30646339 PMC6324361

[386] Administration FaD. NAMENDA^®^ *(Memantine Hydrochloride) Tablets, for Oral Use Initial U.S. Approval: 2003*. (2018). Available at: https://www.accessdata.fda.gov/drugsatfda_docs/label/2018/021487s025lbl.pdf

[387] Tampi RR van Dyck CH. Memantine: efficacy and safety in mild-to-severe Alzheimer's disease. Neuropsychiatr Dis Treat. (2007) 3:245–58. 10.2147/nedt.2007.3.2.245 19300557 PMC2654628

[388] Nair AS Sahoo RK. Efficacy of memantine hydrochloride in neuropathic pain. Indian J Palliat Care. (2019) 25:161–2. 10.4103/IJPC.IJPC_189_18 30820121 PMC6388583

[389] Zdanys K Tampi RR. A systematic review of off-label uses of memantine for psychiatric disorders. Prog Neuropsychopharmacol Biol Psychiatry. (2008) 32:1362–74. 10.1016/j.pnpbp.2008.01.008 18262702

[390] Andrade C. Augmentation with memantine in obsessive-compulsive disorder. J Clin Psychiatry. (2019) 80:19f13163. 10.4088/JCP.19f13163 31846244

[391] Elnaiem W Benmelouka AY Elgendy AMN Abdelgalil MS Brimo Alsaman MZ Mogheeth A. Evaluation of memantine's efficacy and safety in the treatment of children with autism spectrum disorder: a systematic review and meta-analysis. Hum Psychopharmacol. (2022) 37:e2841. 10.1002/hup.2841 35315131

[392] Kern DM Cepeda MS Flores CM Wittenberg GM. Application of real-world data and the REWARD framework to detect unknown benefits of memantine and identify potential disease targets for new NMDA receptor antagonists. CNS Drugs. (2021) 35:243–51. 10.1007/s40263-020-00789-3 33537916 PMC7907035

[393] Matsunaga S Kishi T Iwata N. Memantine for Lewy body disorders: systematic review and meta-analysis. Am J Geriatr Psychiatry. (2015) 23:373–83. 10.1016/j.jagp.2013.11.007 24406251

[394] Sinner B Graf BM. Ketamine. Handb Exp Pharmacol. (2008) 182:313–33. 10.1007/978-3-540-74806-9_15 18175098

[395] Wang X Yang J Hashimoto K. (R)-ketamine as prophylactic and therapeutic drug for neurological disorders: Beyond depression. Neurosci Biobehav Rev. (2022) 139:104762. 10.1016/j.neubiorev.2022.104762 35779628

[396] Taylor CP Traynelis SF Siffert J Pope LE Matsumoto RR. Pharmacology of dextromethorphan: relevance to dextromethorphan/quinidine (Nuedexta^®^) clinical use. Pharmacol Ther. (2016) 164:170–82. 10.1016/j.pharmthera.2016.04.010 27139517

[397] Madeira JM Schindler SM Klegeris A. A new look at auranofin, dextromethorphan and rosiglitazone for reduction of glia-mediated inflammation in neurodegenerative diseases. Neural Regen Res. (2015) 10:391–3. 10.4103/1673-5374.153686 25878586 PMC4396100

[398] Liu Y Qin L Li G Zhang W An L Liu B. Dextromethorphan protects dopaminergic neurons against inflammation-mediated degeneration through inhibition of microglial activation. J Pharmacol Exp Ther. (2003) 305:212–8. 10.1124/jpet.102.043166 12649371

[399] Yang LP Deeks ED. Dextromethorphan/quinidine: a review of its use in adults with pseudobulbar affect. Drugs. (2015) 75:83–90. 10.1007/s40265-014-0328-z 25420446

[400] Schoedel KA Morrow SA Sellers EM. Evaluating the safety and efficacy of dextromethorphan/quinidine in the treatment of pseudobulbar affect. Neuropsychiatr Dis Treat. (2014) 10:1161–74. 10.2147/NDT.S30713 25061302 PMC4079824

[401] Patatanian E Casselman J. Dextromethorphan/quinidine for the treatment of pseudobulbar affect. Consult Pharm. (2014) 29:264–9. 10.4140/TCP.n.2014.264 24704895

[402] Keam SJ. Dextromethorphan/bupropion: first approval. CNS Drugs. (2022) 36:1229–38. 10.1007/s40263-022-00968-4 36301443

[403] Gerlach LB Kales HC. pharmacological management of neuropsychiatric symptoms of dementia. Curr Treat Options Psychiatry. (2020) 7:489–507. 10.1007/s40501-020-00233-9 33344107 PMC7742723

[404] Dudas R Malouf R McCleery J Dening T. Antidepressants for treating depression in dementia. Cochrane Database Syst Rev. (2018) 8:Cd003944. 10.1002/14651858.CD003944.pub2 30168578 PMC6513376

[405] Press D. Management of neuropsychiatric symptoms of dementia. In:Post TW, editor. UpToDate. Waltham, MA: UpToDate Inc. (2023). Available at: http://wwwuptodatecom (accessed September 13, 2023).

[406] Leong C. Antidepressants for depression in patients with dementia: a review of the literature. Consult Pharm. (2014) 29:254–63. 10.4140/TCP.n.2014.254 24704894

[407] Alan F. Schatzberg CBN. The American Psychiatric Association Publishing Textbook Of Psychopharmacology, Fifth Edition. American Psychiatric Association Publishing. 2017:385. 10.1176/appi.books.9781615371624

[408] Burke SL Maramaldi P Cadet T Kukull W. Decreasing hazards of Alzheimer's disease with the use of antidepressants: mitigating the risk of depression and apolipoprotein E. Int J Geriatr Psychiatry. (2018) 33:200–11. 10.1002/gps.4709 28560728 PMC5711617

[409] Correia AS Vale N. Antidepressants in Alzheimer's disease: a focus on the role of mirtazapine. Pharmaceuticals. (2021) 14:930. 10.3390/ph14090930 34577630 PMC8467729

[410] Orgeta V Tabet N Nilforooshan R Howard R. Efficacy of antidepressants for depression in Alzheimer's disease: systematic review and meta-analysis. J Alzheimers Dis. (2017) 58:725–33. 10.3233/JAD-161247 28505970 PMC5467718

[411] Sansone RA Sansone LA. Serotonin norepinephrine reuptake inhibitors: a pharmacological comparison. Innov Clin Neurosci. (2014) 11:37–42. PMC4008300 24800132

[412] Tetsuka S. Depression and dementia in older adults: a neuropsychological review. Aging Dis. (2021) 12:1920–34. 10.14336/AD.2021.0526 34881077 PMC8612610

[413] McGivney SA Mulvihill M Taylor B. Validating the GDS depression screen in the nursing home. J Am Geriatr Soc. (1994) 42:490–2. 10.1111/j.1532-5415.1994.tb04969.x 8176142

[414] Olin JT Schneider LS Katz IR Meyers BS Alexopoulos GS Breitner JC. Provisional diagnostic criteria for depression of Alzheimer disease. Am J Geriatr Psychiatry. (2002) 10:125–8. 10.1176/appi.ajgp.10.2.125 11925273

[415] Orgeta V Qazi A Spector AE Orrell M. Psychological treatments for depression and anxiety in dementia and mild cognitive impairment. Cochr Database Syst Rev. (2014) 2014:Cd009125. 10.1002/14651858.CD009125.pub2 24449085 PMC6465082

[416] Kabir MT Sufian MA Uddin MS Begum M Akhter S Islam A. NMDA receptor antagonists: repositioning of memantine as a multitargeting agent for Alzheimer's therapy. Curr Pharm Des. (2019) 25:3506–18. 10.2174/1381612825666191011102444 31604413

[417] Danysz W Parsons CG. Alzheimer's disease, β-amyloid, glutamate, NMDA receptors and memantine–searching for the connections. Br J Pharmacol. (2012) 167:324–52. 10.1111/j.1476-5381.2012.02057.x 22646481 PMC3481041

[418] Ashford JW. Treatment of Alzheimer's disease: trazodone, sleep, serotonin, norepinephrine, and future directions. J Alzheimers Dis. (2019) 67:923–30. 10.3233/JAD-181106 30776014 PMC6398534

[419] Griffin CE 3rd Kaye AM Bueno FR Kaye AD. Benzodiazepine pharmacology and central nervous system-mediated effects. Ochsner J. (2013) 13:214–23. 10.4414/pc-f.2013.00551 23789008 PMC3684331

[420] Rochon PA Vozoris N Gill SS. The harms of benzodiazepines for patients with dementia. CMAJ. (2017) 189:E517–e8. 10.1503/cmaj.170193 28396327 PMC5386844

[421] Ettcheto M Olloquequi J Sánchez-López E Busquets O Cano A Manzine PR. Benzodiazepines and related drugs as a risk factor in Alzheimer's disease dementia. Front Aging Neurosci. (2019) 11:344. 10.3389/fnagi.2019.00344 31969812 PMC6960222

[422] Joyce G Ferido P Thunell J Tysinger B Zissimopoulos J. Benzodiazepine use and the risk of dementia. Alzheimers Dement. (2022) 8:e12309. 10.1002/trc2.12309 35874428 PMC9297381

[423] Cooper JP. Buspirone for anxiety and agitation in dementia. J Psychiatry Neurosci. (2003) 28:469. 14631458 PMC257799

[424] Desai AK Grossberg GT. Buspirone in Alzheimer's disease. Expert Rev Neurother. (2003) 3:19–28. 10.1586/14737175.3.1.19 19810844

[425] Santa Cruz MR Hidalgo PC Lee MS Thomas CW Holroyd S. Buspirone for the treatment of dementia with behavioral disturbance. Int Psychogeriatr. (2017) 29:859–62. 10.1017/S1041610216002441 28124634

[426] Guaiana G Barbui C Cipriani A. Hydroxyzine for generalised anxiety disorder. Cochr Database Syst Rev. (2010) CD006815. 10.1002/14651858.CD006815.pub2 21154375 PMC13139711

[427] Tannenbaum C Paquette A Hilmer S Holroyd-Leduc J Carnahan R. A systematic review of amnestic and non-amnestic mild cognitive impairment induced by anticholinergic, antihistamine, GABAergic and opioid drugs. Drugs Aging. (2012) 29:639–58. 10.2165/11633250-000000000-00000 22812538

[428] Kim Y Wilkins KM Tampi RR. Use of gabapentin in the treatment of behavioural and psychological symptoms of dementia. Drugs Aging. (2008) 25:187–96. 10.2165/00002512-200825030-00002 18331071

[429] Greenblatt HK Greenblatt DJ. Gabapentin and pregabalin for the treatment of anxiety disorders. Clin Pharmacol Drug Dev. (2018) 7:228–32. 10.1002/cpdd.446 29579375

[430] Markota M Morgan RJ. Treatment of generalized anxiety disorder with gabapentin. Case Rep Psychiatry. (2017) 2017:6045017. 10.1155/2017/6045017 29387502 PMC5745655

[431] Sevigny J Chiao P Bussière T Weinreb PH Williams L Maier M. The antibody aducanumab reduces Aβ plaques in Alzheimer's disease. Nature. (2016) 537:50–6. 10.1038/nature19323 27582220

[432] Vaz M Silva V Monteiro C Silvestre S. Role of aducanumab in the treatment of Alzheimer's disease: challenges and opportunities. Clin Interv Aging. (2022) 17:797–810. 10.2147/CIA.S325026 35611326 PMC9124475

[433] Dhillon S. Aducanumab: first approval. Drugs. (2021) 81:1437–43. 10.1007/s40265-021-01569-z 34324167

[434] Aducanumab. LiverTox: Clinical and Research Information on Drug-Induced Liver Injury. Bethesda, MD: National Institute of Diabetes and Digestive and Kidney Diseases (2012). 31643176

[435] van Dyck CH Swanson CJ Aisen P Bateman RJ Chen C Gee M. Lecanemab in early Alzheimer's disease. N Engl J Med. (2023) 388:9–21. 10.1056/NEJMoa2212948 36449413

[436] Volloch V Rits-Volloch S. Effect of lecanemab in early Alzheimer's disease: mechanistic interpretation in the amyloid cascade hypothesis 20 perspective. J Alzheimers Dis. (2023) 93:1277–84. 10.3233/JAD-230164 37212119 PMC10357217

[437] Burke JF Kerber KA Langa KM Albin RL Kotagal V. Lecanemab: looking before we leap. Neurology. (2023) 101:661–5. 10.1212/WNL.0000000000207505 37479527 PMC10585683

[438] Sims JR Zimmer JA Evans CD Lu M Ardayfio P Sparks J. Donanemab in early symptomatic Alzheimer disease: the TRAILBLAZER-ALZ 2 randomized clinical trial. JAMA. (2023) 330:512–27. 10.1001/jama.2023.13239 37459141 PMC10352931

[439] Rashad A Rasool A Shaheryar M Sarfraz A Sarfraz Z Robles-Velasco K. Donanemab for Alzheimer's disease: a systematic review of clinical trials. Healthcare. (2022) 11:32. 10.3390/healthcare11010032 36611492 PMC9818878

[440] Li X Ji M Zhang H Liu Z Chai Y Cheng Q. Non-drug therapies for Alzheimer's disease: a review. Neurol Therapy. (2023) 12:39–72. 10.1007/s40120-022-00416-x 36376734 PMC9837368

[441] Spector A Thorgrimsen L Woods B Royan L Davies S Butterworth M. Efficacy of an evidence-based cognitive stimulation therapy programme for people with dementia: randomised controlled trial. Br J Psychiatry. (2003) 183:248–54. 10.1192/bjp.183.3.248 12948999

[442] López C Sánchez JL Martín J. The effect of cognitive stimulation on the progression of cognitive impairment in subjects with Alzheimer's disease. Appl Neuropsychol Adult. (2022) 29:90–9. 10.1080/23279095.2019.1710510 31906723

[443] Menna LF Santaniello A Gerardi F Di Maggio A Milan G. Evaluation of the efficacy of animal-assisted therapy based on the reality orientation therapy protocol in Alzheimer's disease patients: a pilot study. Psychogeriatrics. (2016) 16:240–6. 10.1111/psyg.12145 26370064

[444] Camargo CH Justus FF Retzlaff G. The effectiveness of reality orientation in the treatment of Alzheimer's disease. Am J Alzheimers Dis Other Dement. (2015) 30:527–32. 10.1177/1533317514568004 25588408 PMC10852646

[445] García-Navarro EB Buzón-Pérez A Cabillas-Romero M. Effect of music therapy as a non-pharmacological measure applied to Alzheimer's disease patients: a systematic review. Nurs Rep. (2022) 12:775–90. 10.3390/nursrep12040076 36278769 PMC9624344

[446] Matziorinis AM Koelsch S. The promise of music therapy for Alzheimer's disease: A review. Ann N Y Acad Sci. (2022) 1516:11–7. 10.1111/nyas.14864 35851957 PMC9796133

[447] Janata P Tomic ST Rakowski SK. Characterization of music-evoked autobiographical memories. Memory. (2007) 15:845–60. 10.1080/09658210701734593 17965981

[448] Koelsch S. A coordinate-based meta-analysis of music-evoked emotions. Neuroimage. (2020) 223:117350. 10.1016/j.neuroimage.2020.117350 32898679

[449] Lyu J Zhang J Mu H Li W Champ M Xiong Q. the effects of music therapy on cognition, psychiatric symptoms, and activities of daily living in patients with Alzheimer's disease. J Alzheimers Dis. (2018) 64:1347–58. 10.3233/JAD-180183 29991131

[450] Ströhle A Schmidt DK Schultz F Fricke N Staden T Hellweg R. Drug and exercise treatment of Alzheimer disease and mild cognitive impairment: a systematic review and meta-analysis of effects on cognition in randomized controlled trials. Am J Geriatr Psychiatry. (2015) 23:1234–49. 10.1016/j.jagp.2015.07.007 26601726

[451] Matilla-Mora R Martínez-Piédrola RM Fernández Huete J. Effectiveness of occupational therapy and other non-pharmacological therapies in cognitive impairment and Alzheimer's disease. Rev Esp Geriatr Gerontol. (2016) 51:349–56. 10.1016/j.regg.2015.10.006 26613656

[452] Ramin-Wright L Pacheco-Barrios N Zhong S Stokvis-Blok M Barrios-Ruiz A Elhadi A. Effect of occupational therapy on cognition in patients with mild Alzheimer's disease: a systematized literature review. Princip Pract Clin Res. (2023) 9. 10.21801/ppcrj.2023.92.836787223

[453] Gitlin LN Hodgson N Jutkowitz E Pizzi L. The cost-effectiveness of a nonpharmacologic intervention for individuals with dementia and family caregivers: the tailored activity program. Am J Geriatr Psychiatry. (2010) 18:510–9. 10.1097/JGP.0b013e3181c37d13 20847903 PMC2938079

[454] Gitlin LN Winter L Vause Earland T Adel Herge E Chernett NL Piersol CV. The Tailored Activity Program to reduce behavioral symptoms in individuals with dementia: feasibility, acceptability, and replication potential. Gerontologist. (2009) 49:428–39. 10.1093/geront/gnp087 19420314 PMC2682173

[455] Hilgeman MM Allen RS DeCoster J Burgio LD. Positive aspects of caregiving as a moderator of treatment outcome over 12 months. Psychol Aging. (2007) 22:361–71. 10.1037/0882-7974.22.2.361 17563191 PMC2579267

[456] Martínez-Campos A Compañ-Gabucio LM Torres-Collado L Garcia-de la Hera M. Occupational therapy interventions for dementia caregivers: scoping review. Healthcare. (2022) 10:1764. 10.3390/healthcare10091764 36141376 PMC9498417

[457] Smith BC D'Amico M. Sensory-based interventions for adults with dementia and Alzheimer's disease: a scoping review. Occup Therapy Health Care. (2020) 34:171–201. 10.1080/07380577.2019.1608488 31066598

[458] Arora S Santiago JA Bernstein M Potashkin JA. Diet and lifestyle impact the development and progression of Alzheimer's dementia. Front Nutr. (2023) 10:1213223. 10.3389/fnut.2023.1213223 37457976 PMC10344607

[459] Fernández-Sanz P Ruiz-Gabarre D García-Escudero V. Modulating effect of diet on Alzheimer's disease. Diseases. (2019) 7:12. 10.3390/diseases7010012 30691140 PMC6473547

[460] Fiala M Halder RC Sagong B Ross O Sayre J Porter V. ω-3 Supplementation increases amyloid-β phagocytosis and resolvin D1 in patients with minor cognitive impairment. FASEB J. (2015) 29:2681–9. 10.1096/fj.14-264218 25805829

[461] Belkouch M Hachem M Elgot A Lo Van A Picq M Guichardant M. The pleiotropic effects of omega-3 docosahexaenoic acid on the hallmarks of Alzheimer's disease. J Nutr Biochem. (2016) 38:1–11. 10.1016/j.jnutbio.2016.03.002 27825512

[462] Taylor MK Sullivan DK Swerdlow RH Vidoni ED Morris JK Mahnken JD. A high-glycemic diet is associated with cerebral amyloid burden in cognitively normal older adults. Am J Clin Nutr. (2017) 106:1463–70. 10.3945/ajcn.117.162263 29070566 PMC5698843

[463] Taylor MK Swerdlow RH Burns JM Sullivan DK. An experimental ketogenic diet for Alzheimer disease was nutritionally dense and rich in vegetables and avocado. Curr Dev Nutr. (2019) 3:nzz003. 10.1093/cdn/nzz003 30931426 PMC6435445

[464] Margolis LM O'Fallon KS. Utility of ketone supplementation to enhance physical performance: a systematic review. Adv Nutr. (2020) 11:412–9. 10.1093/advances/nmz104 31586177 PMC7442417

[465] Wang M Zhang H Liang J Huang J Chen N. Exercise suppresses neuroinflammation for alleviating Alzheimer's disease. J Neuroinflamm. (2023) 20:76. 10.1186/s12974-023-02753-6 36935511 PMC10026496

[466] Brown BM Peiffer JJ Martins RN. Multiple effects of physical activity on molecular and cognitive signs of brain aging: can exercise slow neurodegeneration and delay Alzheimer's disease? Mol Psychiatry. (2013) 18:864–74. 10.1038/mp.2012.162 23164816

[467] Zeng Y Wang J Cai X Zhang X Zhang J Peng M. Effects of physical activity interventions on executive function in older adults with dementia: A meta-analysis of randomized controlled trials. Geriatr Nurs. (2023) 51:369–77. 10.1016/j.gerinurse.2023.04.012 37127013

[468] Zhang S Zhen K Su Q Chen Y Lv Y Yu L. The effect of aerobic exercise on cognitive function in people with Alzheimer's disease: a systematic review and meta-analysis of randomized controlled trials. Int J Environ Res Public Health. (2022) 19:15700. 10.3390/ijerph192315700 36497772 PMC9736612

[469] Havekes R Heckman PRA Wams EJ Stasiukonyte N Meerlo P Eisel ULM. Alzheimer's disease pathogenesis: the role of disturbed sleep in attenuated brain plasticity and neurodegenerative processes. Cell Signal. (2019) 64:109420. 10.1016/j.cellsig.2019.109420 31536750

[470] Uddin MS Tewari D Mamun AA Kabir MT Niaz K Wahed MII. Circadian and sleep dysfunction in Alzheimer's disease. Ageing Res Rev. (2020) 60:101046. 10.1016/j.arr.2020.101046 32171783

[471] Arkin S. Language-enriched exercise plus socialization slows cognitive decline in Alzheimer's disease. Am J Alzheimers Dis Other Dement. (2007) 22:62–77. 10.1177/1533317506295377 17534004 PMC10697205

[472] Govindugari VL Golla S Reddy SDM Chunduri A Nunna LSV Madasu J. Thwarting alzheimer's disease through healthy lifestyle habits: hope for the future. Neurol Int. (2023) 15:162–87. 10.3390/neurolint15010013 36810468 PMC9944470

[473] Mohammadi S Zandi M Dousti Kataj P Karimi Zandi L. Chronic stress and Alzheimer's disease. Biotechnol Appl Biochem. (2022) 69:1451–8. 10.1002/bab.2216 34152660

[474] Picken J. The coping strategies, adjustment and well being of male inmates in the prison environment. Int J Criminol. (2012) 2012:1–29. Available at: https://www.semanticscholar.org/paper/THE-COPING-STRATEGIES%2C-ADJUSTMENT-AND-WELL-BEING-OF-Picken/a8ba4e2b9bf027e969f7702c3d7aebf433834763

[475] Sawang S Oei T Goh Y Wilman M Markhum E Ranawake D. The ways of coping checklist revision-Asian version (WCCL-ASIAN): a new factor structure with confirmatory factor analysis. Appl Psychol. (2011) 59:202–19. 10.1111/j.1464-0597.2009.00378.x

[476] Sharif Nia H Hosseini L Ashghali Farahani M Froelicher ES. Development and validation of care stress management scale in family caregivers for people with Alzheimer: a sequential-exploratory mixed-method study. BMC Geriatr. (2023) 23:82. 10.1186/s12877-023-03785-6 36750799 PMC9903488

[477] Scheltens P Blennow K Breteler MM de Strooper B Frisoni GB Salloway S. Alzheimer's disease. Lancet. (2016) 388:505–17. 10.1016/S0140-6736(15)01124-1 26921134

[478] Cummings J Lee G Ritter A Sabbagh M Zhong K. Alzheimer's disease drug development pipeline: 2019. Alzheimers Dement. (2019) 5:272–93. 10.1016/j.trci.2019.05.008 31334330 PMC6617248

[479] Bürge E Berchtold A Maupetit C Bourquin NM von Gunten A Ducraux D. Does physical exercise improve ADL capacities in people over 65 years with moderate or severe dementia hospitalized in an acute psychiatric setting? A multisite randomized clinical trial. Int Psychogeriatr. (2017) 29:323–32. 10.1017/S1041610216001460 27831462

[480] Rodríguez-Mansilla J González López-Arza MV Varela-Donoso E Montanero-Fernández J González Sánchez B Garrido-Ardila EM. The effects of ear acupressure, massage therapy and no therapy on symptoms of dementia: a randomized controlled trial. Clin Rehabil. (2015) 29:683–93. 10.1177/0269215514554240 25322869

[481] Narme P Clément S Ehrlé N Schiaratura L Vachez S Courtaigne B. Efficacy of musical interventions in dementia: evidence from a randomized controlled trial. J Alzheimers Dis. (2014) 38:359–69. 10.3233/JAD-130893 23969994

[482] Toots A Littbrand H Lindelöf N Wiklund R Holmberg H Nordström P. Effects of a high-intensity functional exercise program on dependence in activities of daily living and balance in older adults with dementia. J Am Geriatr Soc. (2016) 64:55–64. 10.1111/jgs.13880 26782852 PMC4722852

[483] Burns A Allen H Tomenson B Duignan D Byrne J. Bright light therapy for agitation in dementia: a randomized controlled trial. Int Psychogeriatr. (2009) 21:711–21. 10.1017/S1041610209008886 19323872

[484] Bahar-Fuchs A Martyr A Goh AM Sabates J Clare L. Cognitive training for people with mild to moderate dementia. Cochr Database Syst Rev. (2019) 3:Cd013069. 10.1002/14651858.CD013069.pub2 30909318 PMC6433473

[485] Na R Yang JH Yeom Y Kim YJ Byun S Kim K. A systematic review and meta-analysis of nonpharmacological interventions for moderate to severe dementia. Psychiatry Investig. (2019) 16:325–35. 10.30773/pi.2019.02.11.2 31132836 PMC6539264

[486] Dubois B Hampel H Feldman HH Scheltens P Aisen P Andrieu S. Preclinical Alzheimer's disease: definition, natural history, and diagnostic criteria. Alzheimers Dement. (2016) 12:292–323. 10.1016/j.jalz.2016.02.002 27012484 PMC6417794

[487] Yiannopoulou KG Papageorgiou SG. Current and future treatments in Alzheimer disease: an update. J Cent Nerv Syst Dis. (2020) 12:1179573520907397. 10.1177/1179573520907397 32165850 PMC7050025

[488] Budd Haeberlein S Aisen PS Barkhof F Chalkias S Chen T Cohen S. Two randomized phase 3 studies of aducanumab in early Alzheimer's disease. J Prev Alzheimers Dis. (2022) 9:197–210. 10.14283/jpad.2022.30 35542991

[489] Doody RS Thomas RG Farlow M Iwatsubo T Vellas B Joffe S. Phase 3 trials of solanezumab for mild-to-moderate Alzheimer's disease. N Engl J Med. (2014) 370:311–21. 10.1056/NEJMoa1312889 24450890

[490] Salloway S Farlow M McDade E Clifford DB Wang G Llibre-Guerra JJ. A trial of gantenerumab or solanezumab in dominantly inherited Alzheimer's disease. Nat Med. (2021) 27:1187–96. 34155411 10.1038/s41591-021-01369-8 PMC8988051

[491] Sperling RA Donohue MC Raman R Rafii MS Johnson K Masters CL. Trial of solanezumab in preclinical Alzheimer's disease. N Engl J Med. (2023) 389:1096–107. 10.1056/NEJMoa2305032 37458272 PMC10559996

[492] Salloway S Sperling R Fox NC Blennow K Klunk W Raskind M. Two phase 3 trials of bapineuzumab in mild-to-moderate Alzheimer's disease. N Engl J Med. (2014) 370:322–33. 10.1056/NEJMoa1304839 24450891 PMC4159618

[493] Ostrowitzki S Bittner T Sink KM Mackey H Rabe C Honig LS. Evaluating the safety and efficacy of crenezumab vs placebo in adults with early Alzheimer disease: two phase 3 randomized placebo-controlled trials. JAMA Neurol. (2022) 79:1113–21. 10.1001/jamaneurol.2022.2909 36121669 PMC9486635

[494] Smith J Donohue MC Gruendl E Grimmer T Perry RJ Black SE. GRADUATE I AND II: findings of two phase iii randomized placebo-controlled studies assessing the efficacy and safety of subcutaneous gantenerumab in early Alzheimer's disease (AD) (S26.010). Neurology. (2023) 100(17_supplement_2):4285. 10.1212/WNL.0000000000203868

[495] Teng E Manser PT Pickthorn K Brunstein F Blendstrup M Sanabria Bohorquez S. Safety and efficacy of semorinemab in individuals with prodromal to mild Alzheimer disease: a randomized clinical trial. JAMA Neurol. (2022) 79:758–67. 35696185 10.1001/jamaneurol.2022.1375 PMC9194753

[496] Shulman M Kong J O'Gorman J Ratti E Rajagovindan R Viollet L. TANGO: a placebo-controlled randomized phase 2 study of efficacy and safety of the anti-tau monoclonal antibody gosuranemab in early Alzheimer's disease. Nat Aging. (2023) 3:1591–601. 10.1038/s43587-023-00523-w 38012285 PMC10724064

[497] Florian H Wang D Arnold SE Boada M Guo Q Jin Z. Tilavonemab in early Alzheimer's disease: results from a phase 2, randomized, double-blind study. Brain. (2023) 146:2275–84. 10.1093/brain/awad024 36730056 PMC10232284

[498] Edwards AL Collins JA Junge C Kordasiewicz H Mignon L Wu S. Exploratory tau biomarker results from a multiple ascending-dose study of BIIB080 in Alzheimer disease: a randomized clinical trial. JAMA Neurol. (2023) 80:1344–52. 10.1001/jamaneurol.2023.3861 37902726 PMC10616768

[499] Grimm HP Schumacher V Schäfer M Imhof-Jung S Freskgård PO Brady K. Delivery of the Brainshuttle™ amyloid-beta antibody fusion trontinemab to non-human primate brain and projected efficacious dose regimens in humans. MAbs. (2023) 15:2261509. 10.1080/19420862.2023.2261509 37823690 PMC10572082

[500] Boada M López O Núñez L Szczepiorkowski ZM Torres M Grifols C. Plasma exchange for Alzheimer's disease management by albumin replacement (AMBAR) trial: study design and progress. Alzheimers Dement. (2019) 5:61–9. 10.1016/j.trci.2019.01.001 30859122 PMC6395854

[501] Relkin NR Thomas RG Rissman RA Brewer JB Rafii MS van Dyck CH. A phase 3 trial of IV immunoglobulin for Alzheimer disease. Neurology. (2017) 88:1768–75. 10.1212/WNL.0000000000003904 28381506 PMC5409846

[502] Alves S Fol R Cartier N. Gene therapy strategies for Alzheimer's disease: an overview. Hum Gene Ther. (2016) 27:100–7. 10.1089/hum.2016.017 26838997

[503] Tedeschi DV da Cunha AF Cominetti MR Pedroso RV. Efficacy of gene therapy to restore cognition in Alzheimer's disease: a systematic review. Curr Gene Ther. (2021) 21:246–57. 10.2174/1566523221666210120091146 33494678

[504] Hosseini SA Mohammadi R Noruzi S Mohamadi Y Azizian M Mousavy SM. Stem cell- and gene-based therapies as potential candidates in Alzheimer's therapy. J Cell Biochem. (2018) 119:8723–36. 10.1002/jcb.27202 30074262

[505] Coronel R Lachgar M Bernabeu-Zornoza A Palmer C Domínguez-Alvaro M Revilla A. Neuronal and glial differentiation of human neural stem cells is regulated by amyloid precursor protein (APP) levels. Mol Neurobiol. (2019) 56:1248–61. 10.1007/s12035-018-1167-9 29881946

[506] McGinley LM Kashlan ON Bruno ES Chen KS Hayes JM Kashlan SR. Human neural stem cell transplantation improves cognition in a murine model of Alzheimer's disease. Sci Rep. (2018) 8:14776. 10.1038/s41598-018-33017-6 30283042 PMC6170460

[507] Apodaca LA Baddour AAD Garcia C Jr Alikhani L Giedzinski E Ru N. Human neural stem cell-derived extracellular vesicles mitigate hallmarks of Alzheimer's disease. Alzheimers Res Ther. (2021) 13:57. 10.1186/s13195-021-00791-x 33676561 PMC7937214

[508] Liu Y Huber CC Wang H. Disrupted blood-brain barrier in 5× FAD mouse model of Alzheimer's disease can be mimicked and repaired in vitro with neural stem cell-derived exosomes. Biochem Biophys Res Commun. (2020) 525:192–6. 10.1016/j.bbrc.2020.02.074 32081424

[509] Park D Choi E-K Cho T-H Joo SS Kim Y-B. Human neural stem cells encoding ChAT gene restore cognitive function via acetylcholine synthesis, Aβ elimination, and neuroregeneration in APPswe/PS1dE9 mice. Int J Mol Sci. (2020) 21:3958. 10.3390/ijms21113958 32486466 PMC7313059

[510] Zhu Q Zhang N Hu N Jiang R Lu H Xuan A. Neural stem cell transplantation improves learning and memory by protecting cholinergic neurons and restoring synaptic impairment in an amyloid precursor protein/presenilin 1 transgenic mouse model of Alzheimer's disease. Mol Med Rep. (2020) 21:1172–80. 10.3892/mmr.2020.10918 31922229 PMC7002968

[511] Lu M-H Ji W-L Chen H Sun Y-Y Zhao X-Y Wang F. Intranasal transplantation of human neural stem cells ameliorates Alzheimer's disease-like pathology in a mouse model. Front Aging Neurosci. (2021) 13:650103. 10.3389/fnagi.2021.650103 33776747 PMC7987677

[512] Zhang H-A Yuan C-X Liu K-F Yang Q-F Zhao J Li H. Neural stem cell transplantation alleviates functional cognitive deficits in a mouse model of tauopathy. Neur Regener Res. (2022) 17:314324. 10.4103/1673-5374.314324 34100451 PMC8451553

[513] Kim HJ Cho KR Jang H Lee NK Jung YH Kim JP. Intracerebroventricular injection of human umbilical cord blood mesenchymal stem cells in patients with Alzheimer's disease dementia: a phase I clinical trial. Alzheimers Res Therapy. (2021) 13:154. 10.1186/s13195-021-00897-2 34521461 PMC8439008

[514] Kim J Lee Y Lee S Kim K Song M Lee J. Mesenchymal stem cell therapy and Alzheimer's disease: current status and future perspectives. J Alzheimers Dis. (2020) 77:1–14. 10.3233/JAD-200219 32741816

[515] Kim DY Choi SH Lee JS Kim HJ Kim HN Lee JE. Feasibility and efficacy of intra-arterial administration of embryonic stem cell derived-mesenchymal stem cells in animal model of Alzheimer's disease. J Alzheimers Dis. (2020) 76:1281–96. 10.3233/JAD-200026 32597802

[516] Duan Y Lyu L Zhan S. stem cell therapy for Alzheimer;s disease: a scoping review for 2017–2022. Biomedicines. (2023) 11:120. 10.3390/biomedicines11010120 36672626 PMC9855936

[517] Yang J Li S He X-B Cheng C Le W. Induced pluripotent stem cells in Alzheimer's disease: applications for disease modeling and cell-replacement therapy. Mol Neurodegener. (2016) 11:39. 10.1186/s13024-016-0106-3 27184028 PMC4869261

[518] Manchanda N Aggarwal A Setya S Talegaonkar S. Digital intervention for the management of Alzheimer's disease. Curr Alzheimer Res. (2023). 10.2174/1567205020666230206124155 36744687

[519] Haaksma ML Vilela LR Marengoni A Calderón-Larrañaga A Leoutsakos J-MS Olde Rikkert MG. Comorbidity and progression of late onset Alzheimer's disease: a systematic review. PLoS ONE. (2017) 12:e0177044. 10.1371/journal.pone.0177044 28472200 PMC5417646

[520] Sparks DL Martin TA Gross DR Hunsaker III JC. Link between heart disease, cholesterol, and Alzheimer's disease: a review. Microsc Res Tech. (2000) 50:287–90. 10.1002/1097-0029(20000815)50:4<287::AID-JEMT7>3.0.CO;2-L 10936882

[521] Niu H Álvarez-Álvarez I Guillén-Grima F Aguinaga-Ontoso I. Prevalence and incidence of Alzheimer's disease in Europe: a meta-analysis. Neurologí*a*. (2017) 32:523–32. 10.1016/j.nrleng.2016.02.009 27130306

[522] Doraiswamy PM Leon J Cummings JL Marin D Neumann PJ. Prevalence and impact of medical comorbidity in Alzheimer's disease. J Gerontol Ser A. (2002) 57:M173–M7. 10.1093/gerona/57.3.M173 11867654

[523] Bhargava D Weiner MF Hynan LS Diaz-Arrastia R Lipton AM. Vascular disease and risk factors, rate of progression, and survival in Alzheimer's disease. J Geriatr Psychiatry Neurol. (2006) 19:78–82. 10.1177/0891988706286505 16690992

[524] Wood WG Igbavboa U Eckert GP Johnson-Anuna LN Müller WE. Is hypercholesterolemia a risk factor for Alzheimer's disease? Mol Neurobiol. (2005) 31:185–92. 10.1385/MN:31:1-3:185 15953820

[525] Litke R Garcharna LC Jiwani S Neugroschl J. Modifiable risk factors in Alzheimer disease and related dementias: a review. Clin Ther. (2021) 43:953–65. 10.1016/j.clinthera.2021.05.006 34108080 PMC8440362

[526] Zhang J Chen C Hua S Liao H Wang M Xiong Y. An updated meta-analysis of cohort studies: diabetes and risk of Alzheimer's disease. Diabetes Res Clin Pract. (2017) 124:41–7. 10.1016/j.diabres.2016.10.024 28088029

[527] Whitmer RA Gunderson EP Quesenberry CP Zhou J Yaffe K. Body mass index in midlife and risk of Alzheimer disease and vascular dementia. Curr Alzheimer Res. (2007) 4:103–9. 10.2174/156720507780362047 17430231

[528] Donix M Small GW Bookheimer SY. Family history and APOE-4 genetic risk in Alzheimer's disease. Neuropsychol Rev. (2012) 22:298–309. 10.1007/s11065-012-9193-2 22359096 PMC3797601

[529] Michaelson DM. APOE. ε4: the most prevalent yet understudied risk factor for Alzheimer's disease. Alzheimers Dement. (2014) 10:861–8. 10.1016/j.jalz.2014.06.015 25217293

[530] Werner P. Stigma and Alzheimer's Disease: A Systematic Review of Evidence, Theory, and Methods. American Psychological Association (2014).

[531] Mattsson N Brax D Zetterberg H. To know or not to know: ethical issues related to early diagnosis of Alzheimer's disease. Int J Alzheimers Dis. (2010) 2010:841941. 10.4061/2010/841941 20798843 PMC2925376

[532] Zwaanswijk M Peeters JM Van Beek AP Meerveld JH Francke AL. Informal caregivers of people with dementia: problems, needs and support in the initial stage and in subsequent stages of dementia: a questionnaire survey. Open Nurs J. (2013) 7:6. 10.2174/1874434601307010006 23346266 PMC3551235

[533] Martyr A Clare L. Executive Function and Activities of Daily Living in Alzheimer's Disease: A Correlational Meta-analysis. Switzerland: S. Karger AG Basel (2012). p. 189–203. 10.1159/000338233 22572810

[534] Semiatin AM O'Connor MK. The relationship between self-efficacy and positive aspects of caregiving in Alzheimer's disease caregivers. Aging Ment Health. (2012) 16:683–8. 10.1080/13607863.2011.651437 22360626

[535] Williamson GM Schulz R. Coping with specific stressors in Alzheimer's disease caregiving. Gerontologist. (1993) 33:747–55. 10.1093/geront/33.6.747 8314101

[536] Kim E Baskys A Law AV Roosan MR Li Y Roosan D. Scoping review: the empowerment of Alzheimer's Disease caregivers with mHealth applications. NPJ digital medicine. (2021) 4:131. 10.1038/s41746-021-00506-4 34493819 PMC8423781

[537] Stowell E Zhang Y Castaneda-Sceppa C Lachman M Parker AG. Caring for Alzheimer's disease caregivers: a qualitative study investigating opportunities for Exergame innovation. Proc ACM Hum Comp Interact. (2019) 3:1–27. 10.1145/3359232

[538] Pérez-González A Vilajoana-Celaya J Guàrdia-Olmos J. Alzheimer's disease caregiver characteristics and their relationship with anticipatory grief. Int J Environ Res Public Health. (2021) 18:8838. 10.3390/ijerph18168838 34444587 PMC8392352

[539] Burgio LD Wynn MJ. The REACH OUT Caregiver Support Program: A Skills Training Program for Caregivers of Persons with Dementia, Clinician Guide. Oxford: Oxford University Press (2021).

[540] Sikora Kessler A Mock G Hendricks D Robbins L Kaur H Potter JF. Translating the REACH OUT dementia caregiver intervention into a primary care setting: a pilot study. Aging Ment Health. (2021) 25:1483–92. 10.1080/13607863.2020.1850638 33258686

[541] Khanassov V Rojas-Rozo L Sourial R Yang XQ Vedel I. Needs of patients with dementia and their caregivers in primary care: lessons learned from the Alzheimer plan of Quebec. BMC Fam Pract. (2021) 22:1–9. 10.1186/s12875-021-01528-3 34525960 PMC8441033

[542] Sikkes SA Tang Y Jutten RJ Wesselman LM Turkstra LS Brodaty H. Toward a theory-based specification of non-pharmacological treatments in aging and dementia: focused reviews and methodological recommendations. Alzheimers Dement. (2021) 17:255–70. 10.1002/alz.12188 33215876 PMC7970750

[543] Windle G Algar-Skaife K Caulfield M Pickering-Jones L Killick J Zeilig H. Enhancing communication between dementia care staff and their residents: an arts-inspired intervention. Aging Ment Health. (2020) 24:1306–15. 10.1080/13607863.2019.1590310 30884963 PMC7446032

[544] van Maurik IS Visser LN Pel-Littel RE van Buchem MM Zwan MD Kunneman M. Development and usability of ADappt: web-based tool to support clinicians, patients, and caregivers in the diagnosis of mild cognitive impairment and Alzheimer disease. JMIR Form Res. (2019) 3:e13417. 10.2196/13417 31287061 PMC6643768

[545] Nasreen S Rohanian M Hough J Purver M. Alzheimer's dementia recognition from spontaneous speech using disfluency and interactional features. Front Comp Sci. (2021) 3:640669. 10.3389/fcomp.2021.640669

[546] Morales-de-Jesús V Gómez-Adorno H Somodevilla-García M Vilariño D editors. Conversational system as assistant tool in reminiscence therapy for people with early-stage of alzheimer's. Healthcare. (2021) 9:1036. 10.3390/healthcare9081036 34442173 PMC8391369

[547] Wright EM. The ethical imperative of self-care: a call to action. J Midwif Womens Health. (2020) 65:733–6. 10.1111/jmwh.13150 32869946

[548] Barnett JE Baker EK Elman NS Schoener GR. In pursuit of wellness: the self-care imperative. Prof Psychol. (2007) 38:603a. 10.1037/0735-7028.38.6.603

[549] Acton GJ. Health-promoting self-care in family caregivers. West J Nurs Res. (2002) 24:73–86. 10.1177/01939450222045716 11829276

[550] Gallant MP Connell CM. Predictors of decreased self-care among spouse caregivers of older adults with dementing illnesses. J Aging Health. (1997) 9:373–95. 10.1177/089826439700900306 10182399

[551] Hirano A Suzuki Y Kuzuya M Onishi J Hasegawa J Ban N. Association between the caregiver's burden and physical activity in community-dwelling caregivers of dementia patients. Arch Gerontol Geriatr. (2011) 52:295–8. 10.1016/j.archger.2010.04.011 20462643

[552] McCabe M You E Tatangelo G. Hearing their voice: a systematic review of dementia family caregivers' needs. Gerontologist. (2016) 56:e70–88. 10.1093/geront/gnw078 27102056

[553] Fox KR. The influence of physical activity on mental well-being. Public Health Nutr. (1999) 2:411–8. 10.1017/S1368980099000567 10610081

[554] Bernstein M Munoz N. Position of the Academy of Nutrition and Dietetics: food and nutrition for older adults: promoting health and wellness. J Acad Nutr Diet. (2012) 112:1255–77. 10.1016/j.jand.2012.06.015 22818734

[555] Ryan CT. A Mindful Nation: How a Simple Practice Can Help Us Reduce Stress, Improve Performance, and Recapture the American Spirit. Hay House, Inc. (2012).

[556] Brown KW Ryan RM. The benefits of being present: mindfulness and its role in psychological well-being. J Pers Soc Psychol. (2003) 84:822. 10.1037/0022-3514.84.4.822 12703651

[557] Read A Mazzucchelli TG Kane RT. A preliminary evaluation of a single session behavioural activation intervention to improve well-being and prevent depression in carers. Clin Psychol. (2016) 20:36–45. 10.1111/cp.12084

[558] Roland KP Chappell NL. Meaningful activity for persons with dementia: family caregiver perspectives. Am J Alzheimers Dis Other Dement. (2015) 30:559–68. 10.1177/1533317515576389 25788432 PMC10852756

[559] de la Cuesta-Benjumea C. The legitimacy of rest: conditions for the relief of burden in advanced dementia care-giving. J Adv Nurs. (2010) 66:988–98. 10.1111/j.1365-2648.2010.05261.x 20337791

[560] Eriksson H Sandberg J Hellström I. Experiences of long-term home care as an informal caregiver to a spouse: gendered meanings in everyday life for female carers. Int J Older People Nurs. (2013) 8:159–65. 10.1111/j.1748-3743.2012.00340.x 22805660

[561] Bull MJ. Strategies for sustaining self used by family caregivers for older adults with dementia. J Holist Nurs. (2014) 32:127–35. 10.1177/0898010113509724 24227181

[562] Furlong KE Wuest J. Self-care behaviors of spouses caring for significant others with Alzheimer's disease: the emergence of self-care worthiness as a salient condition. Qual Health Res. (2008) 18:1662–72. 10.1177/1049732308327158 19008361

[563] Verkaik R van Meijel B Verkade P-J Werkman W Hertogh C Francke A. Self-management by family caregivers to manage changes in the behavior and mood of their relative with dementia: an online focus group study. BMC Geriatr. (2016) 16:1–8. 10.1186/s12877-016-0268-4 27142664 PMC4855870

[564] Samson ZB Parker M Dye C Hepburn K. Experiences and learning needs of African American family dementia caregivers. Am J Alzheimers Dis Other Dement. (2016) 31:492–501. 10.1177/1533317516628518 26953236 PMC10852808

[565] Epps F Skemp L Specht JK. How do we promote health?: From the words of African American older adults with dementia and their family members. Res Gerontol Nurs. (2016) 9:278–87. 10.3928/19404921-20160928-01 27855241

[566] Jia J Wei C Chen S Li F Tang Y Qin W. The cost of Alzheimer's disease in China and re-estimation of costs worldwide. Alzheimers Dement. (2018) 14:483–91. 10.1016/j.jalz.2017.12.006 29433981

[567] Zissimopoulos J Crimmins E St Clair P. The value of delaying Alzheimer's disease onset. Forum Health Econ Policy. (2014) 18:25–39. 10.1515/fhep-2014-0013 27134606 PMC4851168

[568] Clay E Zhou J Yi ZM Zhai S Toumi M. Economic burden for Alzheimer's disease in China from 2010 to 2050: a modelling study. J Mark Access Health Policy. (2019) 7:1667195. 10.1080/20016689.2019.1667195 31595183 PMC6764338

[569] Cimler R Maresova P Kuhnova J Kuca K. Predictions of Alzheimer's disease treatment and care costs in European countries. PLoS ONE. (2019) 14:e0210958. 10.1371/journal.pone.0210958 30682120 PMC6347248

[570] Alzheimer's Association. Power of Attorney| Legal Documents| Financial and Legal Planning for Caregivers. (2021). Available at: https://www.alz.org/help-support/caregiving/financial-legal-planning/legal-documents#power

[571] Alzheimer's Association. Guardianship/Conservatorship | legal Documents| Financial and Legal Planning for Caregivers. Available at: https://www.alz.org/help-support/caregiving/financial-legal-planning/legal-documents#guardianship

[572] pearlhealth. Traditional Medicare vs Medicare Advantage (and How Direct Contracting is the Next Step). (2021). Available at: https://pearlhealth.com/blog/healthcare-101/traditional-medicare-vs-medicare-advantage-and-how-direct-contracting-is-the-next-step/

[573] Alzheimer's Association. Medicare| Financial and Legal Planning for Caregivers. (2023). Available at: https://www.alz.org/Help-Support/Caregiving/Financial-Legal-Planning/Medicare

[574] Medicare. Available at: https://www.medicare.gov/

[575] Federal Register. Medicare and Medicaid Programs; CY 2024 Payment Policies Under the Physician Fee Schedule and Other Changes to Part B Payment and Coverage Policies; Medicare Shared Savings Program Requirements; Medicare Advantage; Medicare and Medicaid Provider and Supplier Enrollment Policies; and Basic Health Program. (2023).

[576] de Silva Etges APB Liu HH Jones P Polanczyk CA. Value-based reimbursement as a mechanism to achieve social and financial impact in the healthcare system. J Health Econ Outcomes Res. (2023) 10:100–3. 10.36469/jheor.2023.89151 37928822 PMC10621730

